# Transcriptome profiling of non-climacteric ‘yellow’ melon during ripening: insights on sugar metabolism

**DOI:** 10.1186/s12864-020-6667-0

**Published:** 2020-03-30

**Authors:** Michelle Orane Schemberger, Marília Aparecida Stroka, Letícia Reis, Kamila Karoline de Souza Los, Gillize Aparecida Telles de Araujo, Michelle Zibetti Tadra Sfeir, Carolina Weigert Galvão, Rafael Mazer Etto, Amanda Regina Godoy Baptistão, Ricardo Antonio Ayub

**Affiliations:** 1 Laboratório de Biotecnologia Aplicada a Fruticultura, Departamento de Fitotecnia e Fitossanidade, Universidade Estadual de Ponta Grossa, Av. Carlos Cavalcanti, 4748, Ponta Grossa, Paraná 84030-900 Brazil; 2 Laboratório de Biologia Molecular Microbiana, Departamento de Biologia Estrutural, Molecular e Genética, Universidade Estadual de Ponta Grossa, Av. Carlos Cavalcanti, 4748, Ponta Grossa, Paraná 84030-900 Brazil; 3 Departamento de Bioquímica, Centro Politécnico, Universidade Federal do Paraná, Jd. Das Américas, Caixa-Postal 19071, Curitiba, Paraná 81531-990 Brazil

**Keywords:** *Cucumis melo*, RNA-seq, Sucrose, Fruit ripening, Gene expression

## Abstract

**Background:**

The non-climacteric ‘Yellow’ melon (*Cucumis melo*, *inodorus* group) is an economically important crop and its quality is mainly determined by the sugar content. Thus, knowledge of sugar metabolism and its related pathways can contribute to the development of new field management and post-harvest practices, making it possible to deliver better quality fruits to consumers.

**Results:**

The RNA-seq associated with RT-qPCR analyses of four maturation stages were performed to identify important enzymes and pathways that are involved in the ripening profile of non-climacteric ‘Yellow’ melon fruit focusing on sugar metabolism. We identified 895 genes 10 days after pollination (DAP)-biased and 909 genes 40 DAP-biased. The KEGG pathway enrichment analysis of these differentially expressed (DE) genes revealed that ‘hormone signal transduction’, ‘carbon metabolism’, ‘sucrose metabolism’, ‘protein processing in endoplasmic reticulum’ and ‘spliceosome’ were the most differentially regulated processes occurring during melon development. In the sucrose metabolism, five DE genes are up-regulated and 12 are down-regulated during fruit ripening.

**Conclusions:**

The results demonstrated important enzymes in the sugar pathway that are responsible for the sucrose content and maturation profile in non-climacteric ‘Yellow’ melon. New DE genes were first detected for melon in this study such as invertase inhibitor LIKE 3 (*CmINH3*), trehalose phosphate phosphatase (*CmTPP1*) and trehalose phosphate synthases (*CmTPS5*, *CmTPS7*, *CmTPS9*). Furthermore, the results of the protein-protein network interaction demonstrated general characteristics of the transcriptome of young and full-ripe melon and provide new perspectives for the understanding of ripening.

## Background

Melon (*Cucumis melo* L., Cucurbitaceae) is an economically important fruit crop worldwide that has an extensive polymorphism being classified into 19 botanical groups [1, 2]. This high intra-specific genetic variation is reflected in fruit ripening differences. In this regard, melon fruits present both climacteric and non-climacteric phenotypes. Climacteric fruits are characterized by a respiration peak followed by the autocatalytic synthesis of ethylene, strong aroma, orange pulp, ripening abscission and short shelf life with rapid loss of firmness and taste deterioration (e.g. *cantalupensis* and *reticulatus* melon groups). On the other hand, non-climacteric melon (e.g. *inodorus* melon group) has little ethylene synthesis, white pulp, low aroma, no ripening abscission and a longer shelf life [3–7].

During the ripening process, fruits undergo several biochemical and physiological changes that are reflected in their organoleptic profile, of which the alteration in sucrose accumulation is a determining characteristic in melon quality and consumption [6, 8, 9]. This characteristic is a developmentally regulated process that is related to gene regulation, hormonal signalling and environmental factors [6, 9–11]. Sucrose, glucose and fructose are the major soluble sugars, and sucrose is the predominant sugar in melons at maturity being stored in the vacuoles of the pericarp parenchyma cells [9, 12]. Both climacteric and non-climacteric melons accumulate sugar during fruit ripening [6]. However, the sugar content of *C. melo* species differs according to the genetic variety and development stage [9, 13]. For example, the *flexuosus* melon group presents non-sweet and non-aromatic fruits, and the *cantalupensis* melon group has highly sweet and aromatic fruit [14]. Additionally, in fruit development, sugar is necessary for energy supply, it also generates turgor for fruit cell enlargement and accumulates in late stages of fruit (contributing to fruit taste) [15].

**Fig. 1:**
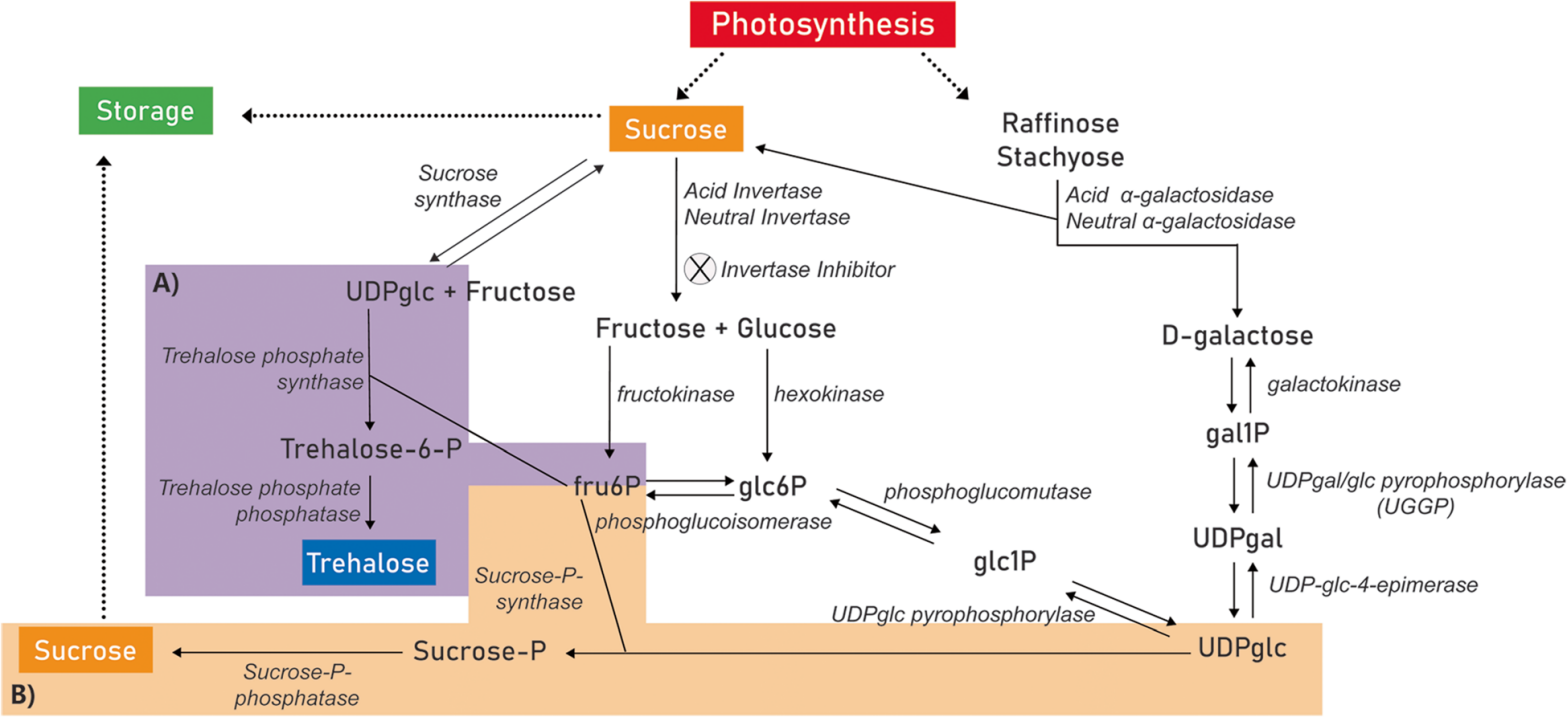
Sugar pathway in *Cucumis melo* demonstrating different routes of sucrose accumulation. UDPglc – Uracil diphosphate glucose; Fruc6P – fructose-6-phosphate; Glc6P – glucose-6-phosphate; Glc1P – glucose-1-phosphate; Gal1P – galactose-1-phosphate; UDP-gal – Uracil diphosphate galactose . Adapted from Chayut et al. (2015) and Dai et al. (2011) [9, 16]. In (A) UDPglc substrate for synthesis of trehalose (B) UDPglc substrate for synthesis of sucrose

Sucrose accumulation in melon fruit is determined by the metabolism of carbohydrates in the fruit sink itself and can be provided from three main sources: (1) photosynthetic product; (2) raffinose family oligosaccharides (RFOs) catabolism; (3) sucrose resynthesis (Fig. 1). In sucrose accumulation, melon plants export sucrose, as well as raffinose family oligosaccharides (RFOs) such as raffinose and stachyose from photosynthetic sources (leaves) to sink tissues (developing melon fruit). RFOs are hydrolyzed by two different families of α-galactosidase (neutral α-galactosidase/NAG or acid α-galactosidase/AAG) producing sucrose and galactose. The synthesized galactose is then phosphorylated by galactokinase (GK) and the resulting galactose 1-phosphate (gal1P) can either participate in the glycolysis pathway through the product glucose-6-phosphate or be used for sucrose synthesis. In sucrose synthesis, galactose 1-phosphate is transformed into glucose 1-phosphate (glc1P) by the actions of UDP-gal/glc pyrophosphorylase (UGGP) and converted to other hexose-phosphates, providing the substrates for the synthesis of sucrose by sucrose-phosphate synthase (SPS) and sucrose-phosphate phosphatase (SPP). Furthermore, sucrose resynthesis is an important pathway and involves many enzymes of sugar metabolism. On the afore-mentioned pathway, sucrose unloaded from the phloem can be hydrolyzed in the apoplast by cell wall invertases (CINs), however, in melon these enzymes may not have a crucial importance once cucurbits have a symplastic phloem loading. Then, the hexose sugar (glucose and fructose) products are imported into the cells by monosaccharide transporters, phosphorylated by hexokinase (HXK) and fructokinase (FK) and used for respiration or sucrose resynthesis. Within the cell, sucrose can be resynthesized in the cytosol by sucrose synthase (SUS) from fructose and UDP-glc. Sucrose can be hydrolyzed to fructose and glucose for energy production also by neutral invertase (NIN), or imported into the vacuole for storage or even hydrolyzed by vacuolar acid invertase (AIN). The invertase activity can be post-translationally regulated by invertase inhibitor proteins (INH) [3, 6, 9, 16–18].

The evidence that shelf life can be related to the sugar accumulation metabolism as well as the relevance of sugar content as a ripeness marker in non-climacteric melon, make sugar metabolism studies important to develop new approaches that can improve its commercial quality. Previous studies have elucidated the peculiarities of carbohydrate metabolism, mainly in climacteric melons [3, 9]. However, understanding of the biochemical aspects that govern the different patterns of sucrose accumulation in the wide genetic variety of melon during the ripening process as well as the identification of new enzymes related to this pathway are limited. Comprehensive molecular studies that could enlighten the complexity of this metabolic pathway are essential. In the last decades, next-generation sequencing (NGS) or high-throughput techniques and metabolomic technologies allowed the generation of a vast amount of information that is essential for the global understanding of metabolic networks. Thus, the aim of our study was to comparatively analyze the transcriptomes of different development and ripening stages of non-climacteric ‘Yellow’ melon (*Inodorus* group) fruits focusing on the sugar pathway. Our analyses provide insights in gene expression ripening profiles of an important Brazilian commercial melon ranked in second position for the total amount of fruits exported by the country (197.60 million metric tons in 2018) [19].

## Results

### Variations in colour, pH and SS (soluble solids) during ripening of melon (*Inodorus* group)

**Fig. 2:**
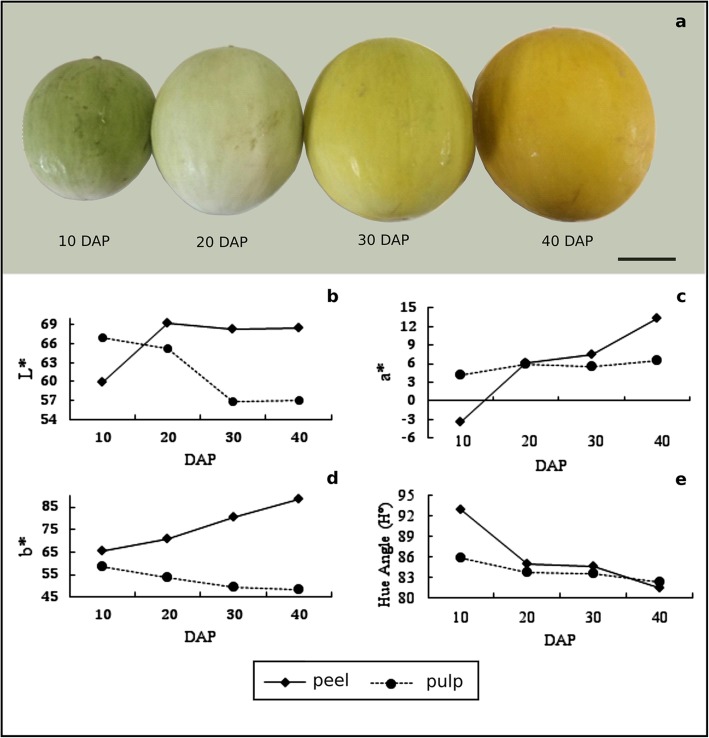
Non-climacteric melon fruit of a ‘Yellow’ commercial genotype of four development stages and its colour characteristics. From left to right there are 10 DAP, 20 DAP, 30 DAP and 40 DAP (**a**). In (**b**) L* (brightness) of peel fruit increases from 10 to 20 DAP and declines in 30 DAP and remains constant until 40 DAP. In (**c**) the coordinates on the a* axis increase during the maturation process in peel and pulp colour, representing the trend change from green to red. In (**d**) coordinates on the b * axis increase during maturation for peel colour, demonstrating the shift from blue to yellow coloration, the opposite profile was found for pulp colour. In (**e**) Hue angle (H°) decreased throughout ripening, corroborating with Kasim and Kasim (2014)

Colour, pH and SS are important characteristics to determine the fruit development stage and changes in its chemical constituents. These parameters were evaluated on non-climacteric melon fruit of the ‘Yellow’ commercial genotype (*Inodorus* group) at 10 days after pollination (DAP), 20 DAP, 30 DAP and 40 DAP (Fig. 2a). Colour measurement was expressed by the CIE (*Commission Internationale de l’Eclairage)* and Hue angle. Colorimeters express colours in numerical terms (see methods) along the L*, a* and b* axes (from white to black, green to red and blue to yellow, respectively) [20]. The results showed that L* (brightness) of peel increases up to 20 DAP, declines up to 30 DAP and remains stable until 40 DAP (Fig. 2b). For pulp colour, there was a decline in brightness until 30 DAPS with later stability (Fig. 2b). Coordinates on the a* axis increased during ripening for peel and pulp, representing the change from green to red (Fig. 2c). Coordinates on the b* axis increase during peel maturation demonstrating a shift from blue to yellow colour, that it is the opposite of the pulp profile (Fig. 2d). Hue angle (H°) is variable as the true colour of the fruit and decreases with maturity, corroborating the findings of Kasim and Kasim (2014) [21] (Fig. 2e). The pH fruit showed a subtle increase during melon maturation (Table 1). Concerning soluble solids (SS) concentration, there is a gradual increase during the ripening process (Table 1).

**Table 1:** pH and Soluble Solids (SS) (° Brix) mean for yellow melon (commercial cultivar) with 10 DAP, 20 DAP, 30 DAP. and 40 DAP

	10 D.A.P.	20 D.A.P.	30 D.A.P.	40 D.A.P.
**pH**	4,15	4,7	4,85	5,1
**SS (°Brix)**	5,0	8,5	10,9	13,3

### Transcriptome sequencing

RNA-seq (RNA sequencing) was carried out on the complementary DNA libraries (cDNA) derived from 10 DAP (two biological replicates) and 40 DAP (three biological replicates) flesh mesocarp. The sequencing data were evaluated for quality, and were subject to data filtering. The results generated ~ 59 million clean single reads of ~ 100 bp in length. A total of ~ 53 million filtered reads were mapped to the *Cucumis melo* reference genome (https://www.melonomics.net) [22, 23] using Bowtie2 [24]. Most sample reads (79.65–97.88%) were successfully aligned and for RNA-seq analysis, only the reads with overlapping in a single gene were considered (Table 2 and Additional file 1: Table S1).

**Table 2:** Number of filtered reads from each sample sequenced and mapped to the *Cucumis melo* (https://www.melonomics.net) reference genome

Sample name	Input reads (filtered)	Mapped reads	% of mapped reads	Detected genes
10DAP_V2	13,444,823	13,159,483	97.88%	16,865
10DAP_V3	11,805,793	10,577,826	89.60%	16,756
40DAP_M1	11,591,063	10,113,177	87.25%	16,161
40DAP_M2	12,635,568	11,246,225	89.00%	15,975
40DAP_M3	10,203,078	8,127,127	79.65%	15,090

### Ripening and development of fruit gene expression profile

**Fig. 3:**
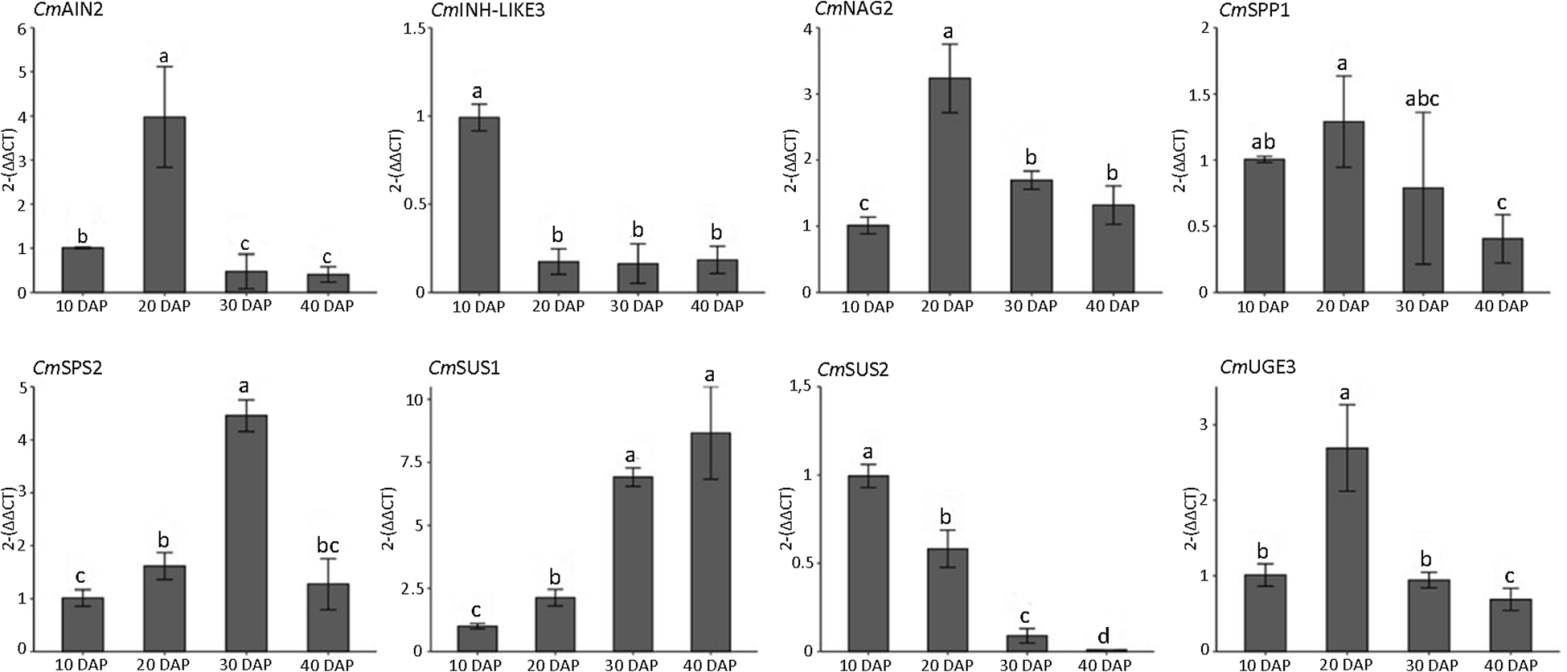
The relative mRNA expression of 9 genes of the sucrose metabolism was determined by 2^-ΔΔCt^ [25]. Results are expressed as mean ± SEM and significance of different developmental stages (10 DAP, 20 DAP, 30 DAP, 40 DAP) comparison is defined as p ≤ 0.05 by Tuckey test after data normalization by Box-Cox method or by Kruskal-Wallis & Wilcoxon (*Cm*SUS1 and *Cm*SUS2). Different letters indicate significant differences

**Fig. 4:**
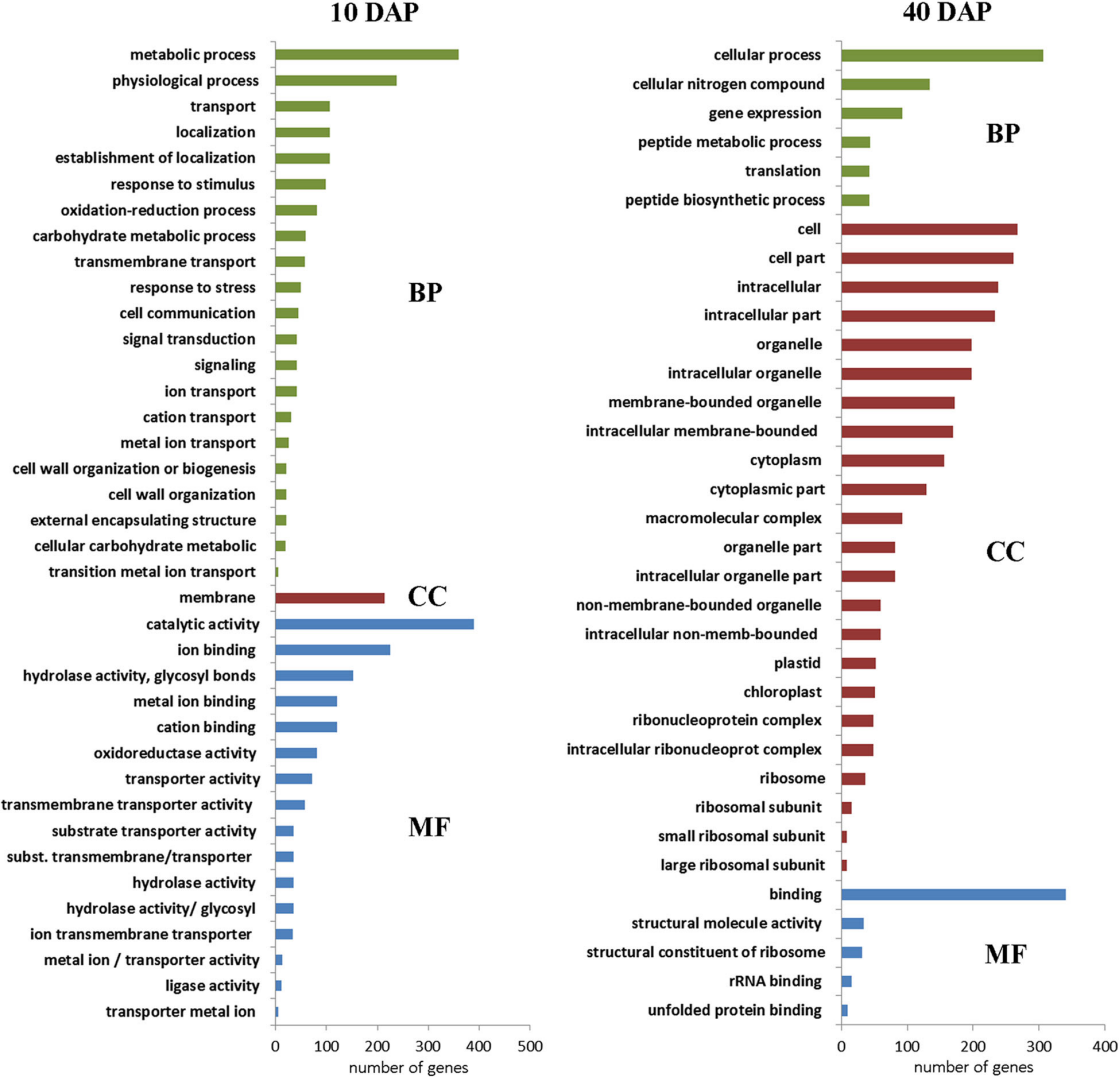
Gene ontology enrichment analysis of the DE genes in the young and mature fruits within category: biological process (BP), cellular component (CC) and molecular function (MF). The analysis was performed using FDR (false discovery rate) adjusted p-value < 0.05 on DE genes (http://cucurbitgenomics.org/goenrich)

RNA-seq is an efficient and powerful tool for studying gene expression. The expression for each gene and differential expression (DE) analyses were calculated by statistical test evaluating the negative binomial distribution, being considered significant padj ≤0.05 (see Methods). In this analysis, over 15,000 expressed genes were detected in each sample (Table 2 and Additional file 1: Table S2) of the 29,980 annotated in the *Cucumis melo* genome [22, 23]. However, a total of 1804 genes showed significant DE between the evaluated stages of fruit maturation (Additional file 1: Table S2). Of these, 895 were 10 DAP-biased and 909 were 40 DAP-biased as demonstrated in MA-plot (Additional file 1: Table S2 and Additional file 2: Figure S1). The RNA-seq data were validated by quantitative reverse transcription PCR analysis (RT-qPCR) of 8 transcripts in the 10 DAP and 40 DAP melons (genes related to the sugar pathway), using *CmRPS15* and *CmRPL* as reference genes (Fig. 3, Additional file 3: Table S3). From pairwise comparison of RNA-seq and RT-qPCR analysis, Pearson’s correlation coefficient was 0.98 (*p* = 0.0014) indicating positive correlation between the two methods (Additional file 3: Figure S2). The sample-to-sample distances that give an overview of similarities and dissimilarities between samples demonstrated clustering of young fruits (10 DAP) separately from the mature fruits (40 DAP) (Additional file 4: Figure S3). Gene ontology (GO) enrichment analysis was performed using FDR (false discovery rate) adjusted *p*-value < 0.05 on DE genes to characterize the differences of ‘Yellow’ melon development and ripening. Figure 4 and Additional File 5: Table S4 show the assigning of GO terms according to the equivalent biological process (BP), molecular function (MF) and cellular component (CC). We found that genes related to BP such as metabolic, physiological, transport and signalling processes were highly enriched in the 10 DAP stage DE genes. On the other hand, DE genes of the 40 DAP fruit were more abundant in the cellular process, cellular nitrogen compound and peptide metabolism BP categories. Under the cellular component classification, the DE genes of the young fruit were only significantly enriched within the ‘membrane’ category, while DE genes of the mature fruit were enriched in several CC terms (e. g. ‘cytoplasm’, ‘chloroplast, ribosomes’). The top 3 groups within the MF classification were ‘catalytic activity’, ‘ion binding’ and ‘hydrolase activity’ for the 10 DAP stage; and ‘binding, structural molecule activity’ and ‘structural constituent of ribosome’ for the 40 DAP stage.

Hierarchical clustering was performed on the 50 most significant DE genes of the 10 DAP and 40 DAP fruits. The clustering of genes was represented in a heatmap (Additional file 6: Table S5 and Figure S4). The results showed 2 genes involved in ‘starch and sucrose metabolism’ (cmo0500) that were more expressed in 10 DAP fruit (beta-glucosidase and sucrose synthase 2); and 2 related to ‘hormone signal transduction’ (cmo04075), being 2 genes more expressed in 10 DAP fruit (xyloglucan endotransglucosylase/hydrolase) and 1 gene more expressed in 40 DAP (pathogenesis-related protein 1-like).

### KEGG enrichment analyses and network construction

The RNA-seq results were subjected to a KEGG pathway enrichment analysis (DAVID software [26]) to elucidate the main pathways involved in fruit ripening and development. A total of 92% (1668/1804) of the DE genes could be converted into UniProtID (available in the DAVID software database). Table 3 shows the top 6 most significantly enriched KEGG pathways for both development stages. The young fruit was enriched with ‘plant hormone signal transduction’ and energetics metabolisms including ‘starch and sucrose metabolism’. The full-ripe melon presented more genes involved with ‘protein processing in endoplasmic reticulum’ and ‘spliceosome’, in addition to energetic metabolisms (Additional File 7: Table S6). Interestingly, the ethylene receptor 1 (MELO3C003906.2) that is a gene of ethylene hormone signal transduction was more expressed in 40 DAP than 10 DAP (Table 4, Additional File 7: Table S6). In this study, we focused on sucrose metabolism (related routes were also considered) because this is an important pathway associated with fruit quality traits. The other pathways will be analyzed in more detail in further studies.

**Table 3:** KEGG pathway analysis of fruit ripening and development candidates genes

10 DAP fruit			
KEGG pathway	Gene count	%	Fisher Exact ***P***-value*
1 Plant hormone signal transduction	21	2.5	4.3E-3
2 Carbon metabolism	16	1.9	6.7E-2
3 Starch and sucrose metabolism	11	1.3	5.7E-3
4 Photosynthesis	7	0.8	2.8E-3
5 Galactose metabolism	7	0.8	1.1E-2
6 Carbon fixation in photosynthetic organisms	6	0.7	7.5E-2
**40 DAP fruit**			
**KEGG pathway**	**Gene count**	**%**	**Fisher Exact** *P*-value*
1 Protein processing in endoplasmic reticulum	24	2.8	5.5E-7
2 Spliceosome	17	2	4.0E-4
3 Carbon metabolism	16	1.9	5.0E-2
4 Ribosome biogenesis in eukaryotes	11	1.3	4.8E-4
5 Carbon fixation in photosynthetic organisms	7	0.8	2.3E-2
6 Pyruvate metabolism	7	0.8	4.9E-2

* Significant P-value ≤0.05

**Table 4:** The differentially expressed genes (RNA-seq analysis) of the sugar pathway (related routes were also considered) in two melon development stages. Statistical test evaluating the negative binomial distribution was applied using R package DeSeq2 (padj ≤0.05)

Melonomics ID (v4.0)	Refseq ID	Short Name	Gene Name	Pathway (KEEG)^a^	Log2 FoldChange^b^	padj
MELO3C010698.2	XP_008444380.1	*CmAAG-LIKE1*	Alpha-galactosidase (Melibiase) Like 1	–	2.1853	3.723E-06
MELO3C004346.2	XP_008448578.1	*CmAGL2*	Alpha-glucosidase 2	–	−2.2809	3.160E-03
MELO3C005109.2	XP_008465523.1	*CmAMN*	Alpha-mannosidase	–	2.4039	4.064E-06
MELO3C035167.2	XP_008463923.1	*CmAUXRF2*	Auxin response factor 2	–	−2.1428	2.295E-02
MELO3C021281.2	XP_008458374.2	*CmBDXY*	Beta-D-xylosidase 1-like	–	2.7858	1.523E-19
MELO3C020906.2	XP_008438779.1	*CmCSREM*	Chromatin structure-remodeling complex protein SYD isoform X1	–	−0.8568	3.099E-02
MELO3C034613.2	XP_008459496.2	*CmCLPP*	CLP protease regulatory subunit CLPX3, mitochondrial isoform X2	–	−1.5961	6.544E-05
MELO3C026854.2	XP_008465290.2	*CmRNApol1*	DNA-directed RNA polymerase subunit	–	−1.1522	2.372E-02
MELO3C016960.2	XP_008452849.1	*CmRNApol2*	DNA-directed RNA polymerase subunit beta	–	−0.9456	4.024E-02
MELO3C010495.2	XP_008446732.1	*CmDNAJ1*	DnaJ protein homolog1	–	−3.5189	1.593E-06
MELO3C012052.2	XP_008446732.1	*CmDNAJ2*	DnaJ protein homolog2	–	−1.6851	4.110E-13
MELO3C006726.2	NP_001284475.1/XP_008438969.1	*CmGK*	Galactokinase	–	0.7983	9.738E-03
MELO3C002363.2	XP_008437427.1	*CmGLMT*	Glucuronoxylan 4-O-methyltransferase 1	–	0.9286	4.704E-02
MELO3C003459.2	XP_008440310.1	*CmGLYT*	Glycosyltransferases	–	2.8172	2.076E-02
MELO3C021249.2	XP_008454693.1	*CmHEXT2*	Hexosyltransferase 2	–	2.0767	1.605E-07
MELO3C015949.2	XP_008447733.1	*CmHEXT1*	Hexosyltransferase 1	–	−2.2774	1.159E-03
MELO3C009735.2	XP_008443230.1	*CmNFKB*	NF-kappa-B-activating protein	–	−1.0229	3.657E-03
MELO3C003497.2	XP_008466126.1	*CmPGLMT1*	Phosphoglycerate mutase-like protein 1	–	2.0468	2.103E-02
MELO3C022069.2	XP_008459427.1	*CmEBGLUC*	Probable endo-1,3(4)-beta-glucanase	–	1.1135	1.504E-02
MELO3C023253.2	XP_008460901.1	*CmPCE1*	Probable pectinesterase1/pectinesterase inhibitor 51	–	5.7293	3.178E-03
MELO3C023254.2	XP_008460902.1	*CmPCE2*	Probable pectinesterase2/pectinesterase inhibitor 51	–	4.8748	4.918E-03
MELO3C023627.2	XP_008438007.1	*CmPGLC1*	Probable polygalacturonase1	–	6.3658	4.436E-02
MELO3C011986.2	XP_008446196.1	*CmPGLC2*	Probable polygalacturonase2	–	2.1382	1.028E-14
MELO3C022542.2	XP_016903497.1/XP_008466011.2	*CmKAN2*	Probable transcription factor KAN2	–	−4.4866	1.861E-02
MELO3C012479.2	XP_008438929.1	*CmPARG1*	Protein argonaute 1	–	−3.0169	1.142E-24
MELO3C021378.2	XP_008460254.1	*CmRIK*	Protein RIK isoform X1	–	−1.3787	1.360E-03
MELO3C006266.2	–	*CmINH-LIKE3*	Putative invertase inhibitor LIKE3	–	2.3543	1.750E-14
MELO3C008049.2	–	*CmINH2*	Invertase inhibitor	–	−1.2754	5.661E-03
MELO3C014613.2	XP_008449737.1	*CmUP1*	uncharacterized protein LOC103491528	–	1.0875	3.548E-02
MELO3C004012.2	XP_008451613.1	*CmUP2*	uncharacterized protein LOC103492844	–	−1.0783	6.416E-03
MELO3C027277.2	XP_008462107.1	*CmEXPGLC*	Exopolygalacturonase clone	cmo00040	2.4207	1.198E-03
MELO3C008202.2	XP_008441351.1	*CmRPE*	Ribulose-phosphate 3-epimerase	cmo00040	1.0046	1.448E-02
MELO3C004075.2	XP_008452100.1	*CmXISM*	Xylose isomerase	cmo00040	0.8185	3.558E-02
MELO3C008467.2	XP_008441609.2	*CmUGGP*	UDP-sugar pyrophosphorylase	cmo00040/ cmo00052/ cmo00520	−1.0021	3.830E-03
MELO3C017213.2	XP_008453254.1	*CmUG6D*	UDP-glucose 6-dehydrogenase	cmo00040/ cmo00520	1.0501	4.919E-02
MELO3C023110.2	–	*CmNAG2*	Neutral alpha galactosidase2	cmo00052	1.0713	2.967E-03
MELO3C011771.2	XP_008445911.1	*CmAAG2*	Alpha-galactosidase (Melibiase)2	cmo00052	1.5211	4.092E-02
MELO3C032910.2	XP_008440953.1	*CmATP-PPKN*	ATP-dependent 6-phosphofructokinase (Phosphofructokinase)	cmo00052	1.1856	3.992E-02
MELO3C009979.2	XP_008443553.1	*Cm NAGLIKE2*	Galactinol-sucrose galactosyltransferase 5	cmo00052	2.4512	2.042E-04
MELO3C010314.2	XP_008443958.1	*CmNAG3*	Galactinol-sucrose galactosyltransferase 6 isoform X1	cmo00052	−0.9338	2.874E-02
MELO3C015912.2	XP_008451468.1	*CmSCS*	Stachyose synthase	cmo00052	2.4118	7.679E-04
MELO3C005363.2	NP_001284469.1	*CmAIN2*	Acid Invertase 2 (acid beta-fructofuranosidase-like)	cmo00052/ cmo00500	2.3430	1.957E-08
MELO3C005293.2	XP_008467118.1	*CmPGIcyt*	Phosphoglucomutase, cytoplasmic	cmo00052/ cmo00500	0.8393	1.918E-02
MELO3C017002.2	XP_008452915.1	*CmAAML*	Alpha-amylase (1,4-alpha-D-glucan glucanohydrolase)	cmo00500	0.9488	1.996E-02
MELO3C012010.2	XP_008446229.1	*CmTPS9*	Alpha-trehalose-phosphate synthase [UDP-forming] 9	cmo00500	1.1210	8.375E-03
MELO3C016121.2	XP_008451866.1	*CmBAML*	Beta-amylase	cmo00500	−1.2463	6.429E-03
MELO3C034277.2	XP_008453064.1	*CmBGL18*	Beta-glucosidase 18-like	cmo00500	1.7017	1.035E-04
MELO3C015214.2	XP_008450452.1	*CmBGL24*	Beta-glucosidase 24	cmo00500	4.5276	6.270E-04
MELO3C021895.2	XP_008459280.1	*CmEGLC*	Endoglucanase-like	cmo00500	7.8063	3.544E-05
MELO3C002024.2	XP_008440956.1	*CmGBGL1*	Glucan endo-1,3-beta-glucosidase 1	cmo00500	−1.8416	4.230E-02
MELO3C030768.2	XP_016900389.1	*CmIBAML*	Inactive Beta-amylase	cmo00500	1.7307	4.735E-02
MELO3C015552.2	XP_008450968.1	*CmSUS1*	Sucrose synthase 1	cmo00500	−1.2647	5.108E-05
MELO3C025101.2	XP_008463167.1	*CmSUS2*	Sucrose synthase 2	cmo00500	3.8280	1.430E-41
MELO3C009570.2	XP_008442968.1	*CmSPP1*	Sucrose-phosphatase 1	cmo00500	0.8290	4.230E-02
MELO3C020357.2	XP_008457154.1	*CmSPS2*	Sucrose-phosphate synthase 2	cmo00500	1.6220	4.051E-03
MELO3C006984.2	XP_008439346.1	*CmTPP1*	Trehalose 6-phosphate phosphatase 1	cmo00500	3.7901	4.521E-02
MELO3C018715.2	XP_016901732.1	*CmTPS7*	Trehalose-6-phosphate synthase 7	cmo00500	−0.6675	4.521E-02
MELO3C013838.2	XP_008448661.1	*CmTPS5*	Trehalose-6-phosphate synthase 5	cmo00500	−1.0657	6.854E-04
MELO3C005858.2	XP_008437557.1	*CmAEChit*	Acidic endochitinase	cmo00520	2.1904	4.663E-05
MELO3C009722.2	XP_008443206.1	*CmALAR*	Alpha-L-arabinofuranosidase 1-like isoform X2	cmo00520	2.5093	2.053E-17
MELO3C006704.2	XP_008444611.1	*CmEP3-Like*	Endochitinase EP3-like	cmo00520	2.1214	1.476E-02
MELO3C005859.2	XP_016903343.1	*CmHV-ALIKE*	Hevamine-A-like	cmo00520	4.4816	5.239E-03
MELO3C019691.2	XP_016902486.1	*CmHEXT3*	Hexosyltransferase 3	cmo00520	1.2974	1.711E-02
MELO3C005640.2	XP_008451740.1	*CmUGE3*	UDP-glucose epimerase 3	cmo00520	1.5495	6.583E-07
MELO3C022932.2	XP_008460595.1	*CmAUXRF1*	Auxin response factor1	cmo04075	−0.7397	5.303E-03
MELO3C003906.2	XP_008450396.1	*CmER1*	Ethylene receptor 1	cmo04075	−1.2850	1.144e-06
MELO3C006371.2	XP_008461049.1	*CmAUXRS*	Auxin-resposive protein	cmo04075	−1.7529	1.147E-02
MELO3C011021.2	XP_008444821.1	*CmENDP*	Endoplasmin homolog	cmo04141	−0.7411	4.808E-02

^a^ cmo00040: pentose and glucuronate interconversions; cmo00052: galactose metabolism; cmo00500: starch and sucrose metabolism; cmo00520: amino sugar and nucleotide sugar metabolism; cmo04075: plant hormone signal transduction; cmo04141: protein processing in endoplasmic reticulum

^b^ The positive values are up-regulated genes and the negative values are down-regulated genes when considerate the 10 DAP stage

**Fig. 5:**
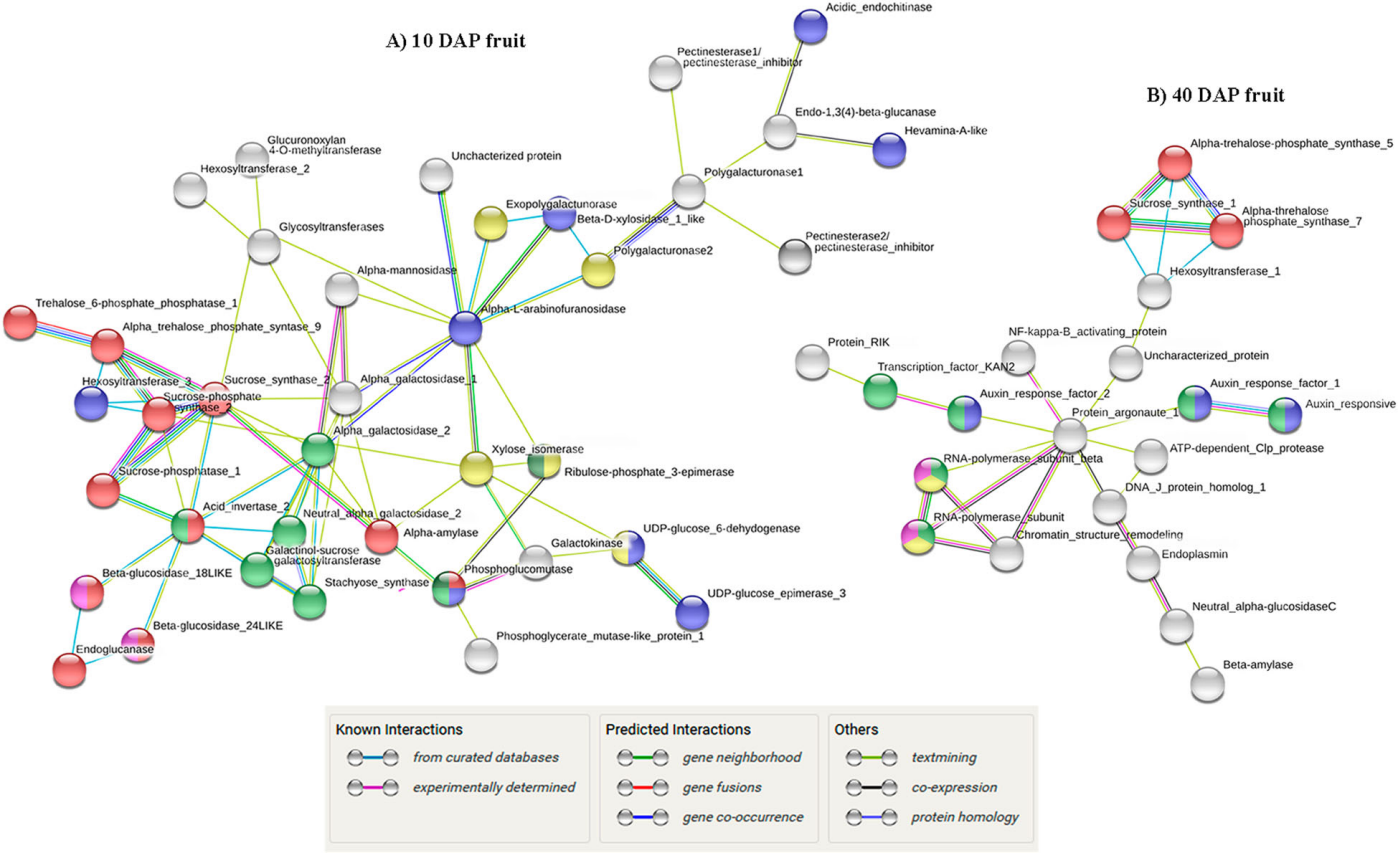
Protein–protein interaction network of sugar pathway and associated routes in the 10 DAP (**a**) and 40 DAP (**b**) melon fruits obtained by STRING analyses. Nodes represent related proteins and edges represent protein–protein associations. In A) red is “Starch and sucrose metabolism”, blue is “Amino and nucleotide sugar metabolism”, light green is “Galactose metabolism”, yellow is “Pentose and glucoronate interconversions”, pink is “Cyanoamino acid metabolism” and dark green is “Pentose phosphate pathway”. In B) red is “Starch and sucrose metabolism”, blue is “Auxin signalling pathway”, green is “Transcription”, yellow is “Nucleotidyltransferase” and pink is “DNA-directed RNA polymerase”. The white nodes are genes not classified within a pathway or protein group

For network construction, we used the STRING database (https://string-db.org) that returned 417 nodes, 671 edges and the *p*-value for protein-protein interaction (PPI) enrichment was < 1.0e-16 for 10 DAP fruit genes (Additional file 8: Figure S5, Table S7). The 40 DAP fruit genes results showed 404 nodes, 1512 edges and the p-value PPI enrichment was < 5.79e-08 (Additional file 8: Figure S6, Table S8). The functional enrichment in the network demonstrated a high number of proteins involved in metabolic pathways and protein processing in the young and full-ripe fruit respectively (Additional file 8: Figure S5, S6). Proteins related to the sugar pathway were selected from total DE genes and the subnetwork generated was composed of 38 nodes, 68 edges and PPI enrichment p-value < 1.0e-16 in young fruit. The proteins with the highest interaction in this analysis were alpha-N-arabinofuranosidase 1 (XP_008443206.1), sucrose synthase (XP_008463167.1) and acid invertase 2 (NP_001284469.1) (Fig. 5, Additional file 9: Tables S9, S10). Regarding the mature fruit, the subnetwork generated was characterized by 22 nodes, 27 edges and PPI enrichment p-value < 1.0e-16. The protein argonaute 1 (XP_008438929.1) and probable galacturonosyltransferase 10 (XP_008447733.1) presented the highest interactions number (Fig. 5, Additional file 9: Tables S11, S12).

### Sugar pathway and associated proteins

**Fig. 6:**
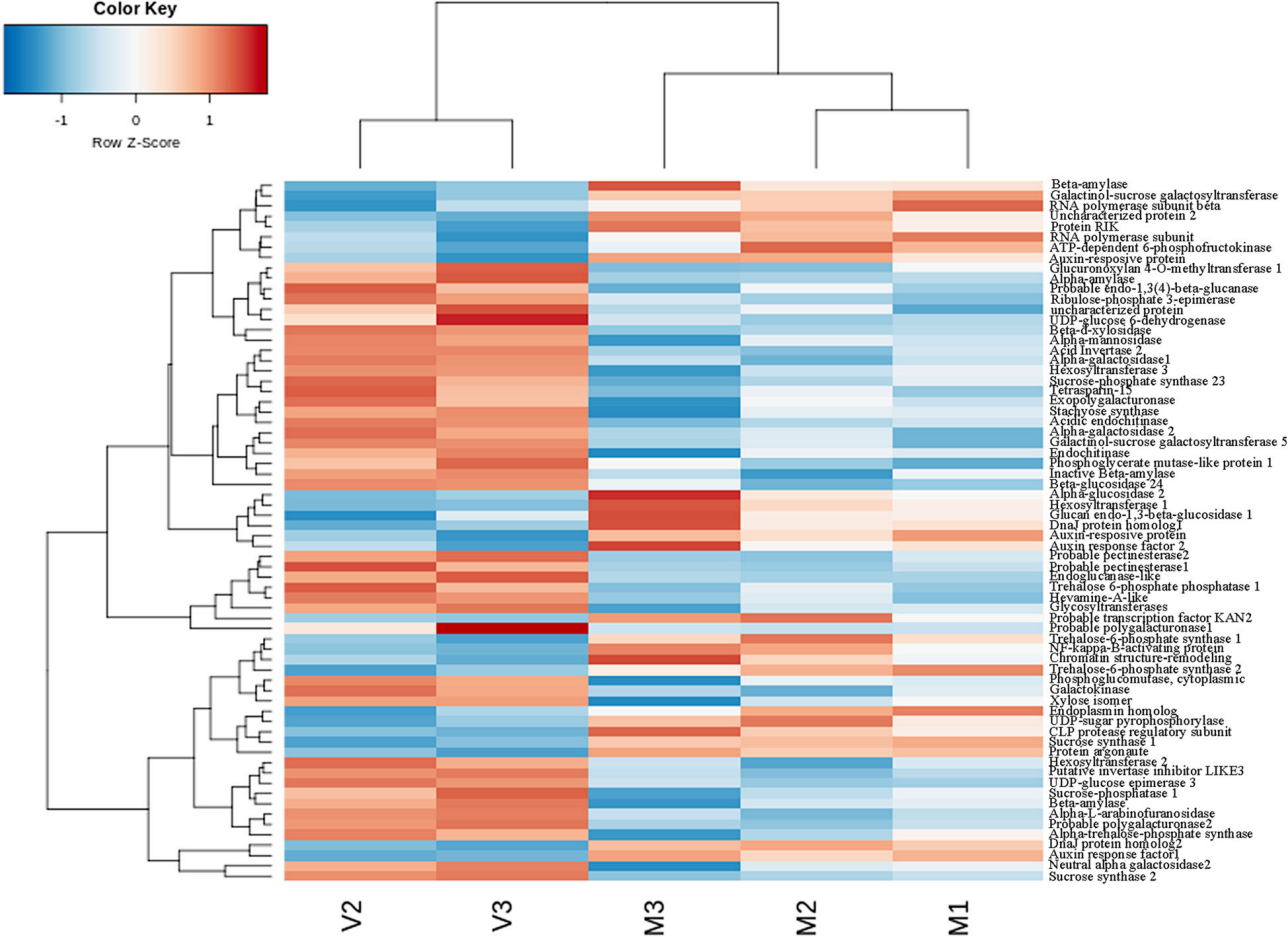
Hierarchical clustering analyses of DE genes of sugar and associated pathways of young (10 DPA) and mature (40 DAP) fruit samples. The log2 fold change values were converted by rlog (regularized logarithm) function in Deseq2. Each line represents one gene and the rows are the samples. The colour bar represents the rlog values and ranges from blue (low expression) to red (high expression)

Seventeen DE genes are associated with the sucrose metabolism by KEGG analyses (Fig. 6, Table 4, Additional file 10: Figure S7, Additional file 11: Figure S10). The genes that present higher interaction with these enzymes (STRING database) by PPI analyses and those that are important in sucrose metabolism described in previous studies (not available in the KEGG database) were also considered [9, 16] (Fig. 6, Table 4, Additional file 10: Figures S8, S9, Additional file 11: Figure S10). Some enzymes associated with this pathway are encoded by multiple genes and their amino acid sequences were aligned using the MUSCLE algorithm [27] as well as submitted to percentage similarity analysis (http://imed.med.ucm.es/Tools/sias.html software). The results showed a wide difference between the isoenzymes; and the alpha galactosidases, invertase inhibitor and hexosyltransferase sequences were the most dissimilar (Additional file 12: Figure S11).

Considering RNA-seq analysis, in the ‘starch and sucrose metabolism’ (KEGG: cmo00500) 12 genes are more expressed in young fruit (acid invertase 2/ *CmAIN2*, phosphoglucomutase/*CmPGIcyt*, alpha-amylase/*CmAAML*, alpha-trehalose-phosphate synthase9/*CmTPS9*, beta-glucosidase 18-like/*CmBGL18*, beta-glucosidase 24/*CmBGL24*, endoglucanase-like/*CmEGLC*, inactive beta-amylase/*CmIBAML*, sucrose synthase 2/*CmSUS2*, sucrose-phosphatase1/*CmSPP1*, sucrose-phosphate synthase 2/*Cm*SPS2, trehalose 6-phosphate phosphatase 1/*CmTPP1*); and 5 genes are more expressed in full-ripe fruit (beta-amylase/*CmBAML*, glucan endo-1,3-beta-glucosidase 1/*CmGBGL1*, sucrose synthase 1/*CmSUS1*, trehalose-6-phosphate synthase 7/*CmTPS7*, trehalose-6-phosphate synthase 5/*CmTPS5*) (Table 4, Additional file 11: Figure S10). The highest log2 fold change values were to *CmEGLC* (7.8063) and *CmBGL24* (4.5276) in young melon. For mature melon they were to *CmGBGL1* (1.8416) and *CmSUS1* (1.2647) (Table 4). The RT-qPCR (quantitative reverse transcription PCR analysis) was conducted for some of these genes in the 10 DAP, 20 DAP, 30 DAP and 40 DAP stages (Fig. 3). In this analysis, the *CmAIN2* gene has a markedly increased expression from 10 to 20 DAP fruit, declining rapidly in subsequent stages (Fig. 3). The two sucrose synthase isoenzymes showed different expression patterns in fruit maturation as also observed in RNA-seq. *CmSUS1* relative expression has a continuous increase from 10 DAP to 40 DAP fruit. In contrast, the *CmSUS2* gene has a higher expression level in younger fruit and gradually decreased in the following ripening stages (Fig. 3). The expression level of *CmSPS2* was more remarkable in 30 DAP fruits when compared to other maturation stages (Fig. 3). *CmSPP1* expression increased from 10 DAP to 20 DAP and then decreased in the following developmental stages (Fig. 3). The *CmINH-LIKE3* is not presented in the KEGG pathway; however, it has been included in RT-qPCR analyses because the literature reports its function in invertase inhibition. The expression profile of this gene demonstrated a marked expression only in younger fruit when compared to other development stages (Fig. 3). However, the *CmINH2* isoform presented higher expression in 40 DAP fruit when compared to 10 DAP fruit (RNA-seq analysis).

In the ‘amino sugar and nucleotide sugar metabolism’ (cmo00520), 7 genes are more expressed in 10 DAP fruit (UDP-glucose 6-dehydrogenase/*CmUG6D*, Acidic endochitinase/*CmAEChit*, Alpha-L-arabinofuranosidase 1-like isoform/*CmALAR*, Endochitinase EP3-like/*CmEP3-Like*, Hevamine-A-like/*CmHV-ALIKE*, Hexosyltransferase 3/*CmHEXT3*, UDP-glucose epimerase 3/*CmUGE3*) and 1 gene is more expressed in 40 DAP (UDP-sugar pyrophosphorylase/*CmUGGP*) (Table 4, Additional file 11: Figure S10). The most representative expression level was to *CmHV-ALIKE* (4.4816). In the RT-qPCR analysis, the gene expression of *CmUGE3* was relatively low in young fruit, increased rapidly in the 20 DAP stage and decreased in the following developmental stages (Fig. 3).

The ‘galactose metabolism’ (cmo00052) has 9 DE genes, 6 of them more expressed in young fruit (Alkaline alpha-galactosidase/*CmNAG2*, Alpha-galactosidase 2/*CmAAG2*, Galactinol-sucrose galactosyltransferase 5/*CmNAGLIKE2*, Stachyose synthase/*CmSCS*, Acid Invertase 2/*CmAIN2*, Phosphoglucomutase/*CmPGIcyt*) and 3 more expressed in mature fruit (UDP-sugar pyrophosphorylase/*CmUGGP*, ATP-dependent 6-phosphofructokinase/*CmATP-PPKN*, Galactinol-sucrose galactosyltransferase 6 isoform X1/*CmNAG3*) (Table 4, Additional file 11: Figure S10). The relative expression of the *CmNAG2* gene showed a rapid increase from 10 DAP to 20 DAP decreasing in 30 DAP and keeping constant in 40 DAP (Fig. 3). The RT-qPCR of *CmAIN2* has been previously discussed.

Also, another 32 DEGs were identified in network analyses that are potentially associated with the sugar pathway (Table 4, Additional file 11: Figure S10). The more expressed genes were: probable polygalacturonase1 (6.3658), probable pectinesterase1 (5.7293), probable pectinesterase 2 (4.8748) for young fruits and probable transcription factor KAN2 (4.48), DnaJ protein homolog1 (3.51), Protein argonaute 1 (3.016) for full-ripe fruits.

## Discussion

### Global characteristics of the ‘yellow’ non-climacteric melon ripening

Fruit ripening and development is a genetically programmed and irreversible process that involves physiological, biochemical and organoleptic changes influencing the fruit quality such as flavour, texture, colour and aroma [28]. However, the study of the metabolic networks is complex and the central signal of genic cascade is not completely understood. In our study, we used an important commercial non-climacteric ‘Yellow’ melon fruit (*Cucumis melo, inodorus* group) as experimental material to comprehend the main metabolic processes that involve maturation in this phenotype, focusing on the sugar pathway study that is a main quality attribute in melon fruits.

RNA-seq technology was used to analyze the transcriptomic differences between young (10 DAP) and mature (40 DAP) non-climacteric melon fruit. A total of 895 DE genes are down-regulated and 909 are up-regulated during melon ripening. GO enrichment analysis showed that the DE genes in young fruit were more related to molecular transport and metabolic processes including the ‘carbohydrate metabolism’; while in ripe fruit the most DE genes are required for peptide metabolism and protein biosynthesis. In addition, the integrative KEGG analysis conducted for metabolic pathways demonstrated that ‘carbon fixation in photosynthetic organisms’ and ‘carbon metabolism’ pathways were enriched in both fruit development stages; however different genes or isoforms are DE. At the beginning of fruit development there is high anabolism and catabolism of sugar that is the metabolic process required for carbon skeleton construction and energy supply in plants. In strawberry fruit, an important role of oxidative phosphorylation in ripening was demonstrated [29]. The DE genes enriched in 40 DAP melon fruits are related to the sucrose accumulation function [6]. The protein processes in the endoplasmic reticulum, spliceosome mechanism and ribosome biogenesis were also significantly enriched in KEGG analysis in the late development of melon indicating high transcription and translation rate. Moreover, the high splicing process is reflected in the production of different proteins that can act and control a specific metabolic route. This characteristic associated with the activation of different protein isoforms can also explain the KEGG enrichment of the same pathways in both maturation stages as previously mentioned. Studies have reported the presence of paralogous copies acting in diverse metabolic pathways in plants, including in sugar metabolism, that in melon have definite functionalization concerning both development stages and tissue specificity [9]. The high activity of photosynthesis in young melon when compared to full-ripe fruit has also been described for grape and other melon varieties [6, 30].

The ‘plant hormone signal transduction’ is an important process in fruit ripening [31, 32], and this pathway was significantly enriched in the early melon fruit development. Ethylene (ETH), abscisic acid (ABA) and brassinosteroids (BRs) have been suggested to promote ripening through complex interactions; while auxin (IAA), cytokinins (CYT), gibberellin (GA) and jasmonic acid (JA) are putative inhibitors of ripening [29, 33]. In our study, the DE genes present in IAA, JA, GA and CYT signal transduction decreased during maturation which also occurs in other non-climacteric fruits [29, 34, 35]. In the ABA pathway, only the ‘protein phosphatase 2C 77’ gene (repressor of the abscisic acid signalling pathway [36]) was DE in the 10 DAP fruit. Studies have been suggested that ABA plays an important role in the regulation of non-climacteric fruits [37, 38] and the key gene for its biosynthesis is 9-cis-epoxycarotenoid dioxygenase (*CmNCED*) that was significantly more expressed in full-ripe fruit (Additional file 7: Table S6). This can indicate that ABA might be involved in the regulation of melon maturation and senescence. Interestingly, there are intimate connections between sugar and ABA signalling [39]. The BR burst production generally occurs in the colour change stage in late fruit development [29, 33, 40]. In our study, the genes related to BR signal transduction are more expressed in young ‘Yellow’ melon fruit; however the colour change occurs from 20 DAP to 30 DAP fruits. Thus further studies should be conducted to understand the transcriptome profile of these stages. The expression of some genes present in the ethylene and salicylic acid metabolism were highest in mature fruit (Additional file 7: Table S6). One of these genes is the ethylene receptor 1 that has been shown to negatively regulate ethylene signal transduction and suppress ethylene responses [41]. Thus, it can be a candidate gene in non-climacteric and climacteric melon comparative study.

In the subnetwork protein-protein interaction (PPI), the results of the 10 DAP fruits showed the interaction of 6 metabolic pathways: ‘Starch and sucrose metabolism’; ‘Amino and nucleotide sugar metabolism’; ‘Galactose metabolism’; ‘Pentose and glucuronate interconversions’; ‘Cynoamino acid metabolism’; and ‘Pentose phosphate pathway’. Furthermore, enzymes related to cell wall degradation were identified such as pectinesterase and polygalacturonase that are mainly responsible for the pectin changes. The up-regulation of these genes and those associated with sucrose synthesis in the early stage of development are involved with progressive fruit softening and sucrose accumulation. In flesh watermelon, some isoforms of pectinesterase and polygalacturonase also show an increase in the first development stages, decreasing in the full-ripe fruit [42]. Another cell wall enzyme was alpha-L-arabinofuranosidase that catalyzes the breaks in the arabinoxylan (major component of cell wall plant hemicellulose) [43]. Saladié et al. (2015) demonstrated that several genes related to cell wall degradation were more strongly up-regulated in climacteric melon (cv. Védrantais) than non-climacteric (cv. Piel de Sapo) [6]. The sugar metabolism is an important process in fruit ripening and development and sucrose accumulation is the major determinant of melon sweetness [6, 44]. One enzyme of this pathway is the acid invertase (*CmAIN2*) that had the second highest number of interactions in the young melon fruit subnetwork (Fig. 5; Additional file 9: Table S9). This reinforces the idea of its key function in the catabolism of sucrose [6]. Two beta-glucosidases (*CmGL18*, *CmGL24*) have an interaction with *CmAIN2*, these enzymes have the function of hydrolyzing the terminal, non-reducing beta-D-glucosyl residues (final reaction in cellulose hydrolysis) with the release of beta-D-glucose (primary energy source in plants) [45] that suggest a high sugar conversion to energy in the early fruit development stage. Another important enzyme in the subnetwork is sucrose synthase 2 (*CmSUS2*) that has a strong interaction with alpha-trehalose phosphate synthase 9 (*CmTPS9*) followed by trehalose phosphate phosphatase (*CmTPP1*). These enzymes and others of sugar metabolism will be discussed in more detail below in the next topic.

Although the majority of DE genes of auxin and sugar metabolism are up-regulated in 10 DAP melons, some isoforms or different genes from these pathways are more expressed in 40 DAP. The subnetwork generated for mature fruit is represented by a different sucrose synthase (*CmSUS1*) which also has high interaction with two alpha-trehalose phosphate synthase isoenzymes (*CmTPS7*, *CmTPS5*). The trehalose phosphate synthases (TPS) convert glucose-6-phosphate and uridine diphosphate (UDP) glucose into trehalose-6phosphate (T6P) and the subsequent dephosphorization of T6P is catalyzed by trehalose-phosphate phosphatases. A recent study reported that threalose-6-phosphate inhibited sucrose synthase and consequently the sucrose cleavage in castor bean [46]. The T6P may be undergoing a higher conversion into trehalose in young melon due to the greater trehalose-phosphate phosphatase gene expression. Thus T6P accumulation is expected in full-ripe fruit, once that TPP is down-regulated, contributing to the increase of sucrose content [47]. In addition, genes involved with the auxin pathway such as auxin response factor (*CmAUXRF1*, *CmAUXRF2*) and responsive auxin protein (*CmAUXRS*) are present in this subnetwork and have interaction through hexosyltransferase and argonaute proteins with the *CmSUS1*, *CmTPS7* and *CmTPS5* (Fig. 5). In that respect, previous studies reported that auxin reduces the sugar content in fruits [48]. However, the precise association of the genes *CmAUXRF1*, *CmAUXRF2* and *CmAUXRS* with the sugar pathway requires further studies. Regarding the argonaute proteins, they bind to micro RNAs (miRNA) and act in transcript cleavage [49]. Plant miRNAs typically target transcription factors including the auxin-response factor [49]. A weak interaction was detected between hexosyltransferases, unknown proteins and argonaute proteins and further studies should be conducted to better understand this association. It is also noteworthy that the chromatin structure-remodelling complex protein SYD (*CmCSREM*) gene present in this subnetwork is related to a promotor regulation of several genes downstream of the jasmonate and ethylene signalling pathways [50].

### Sucrose metabolism

**Fig. 7:**
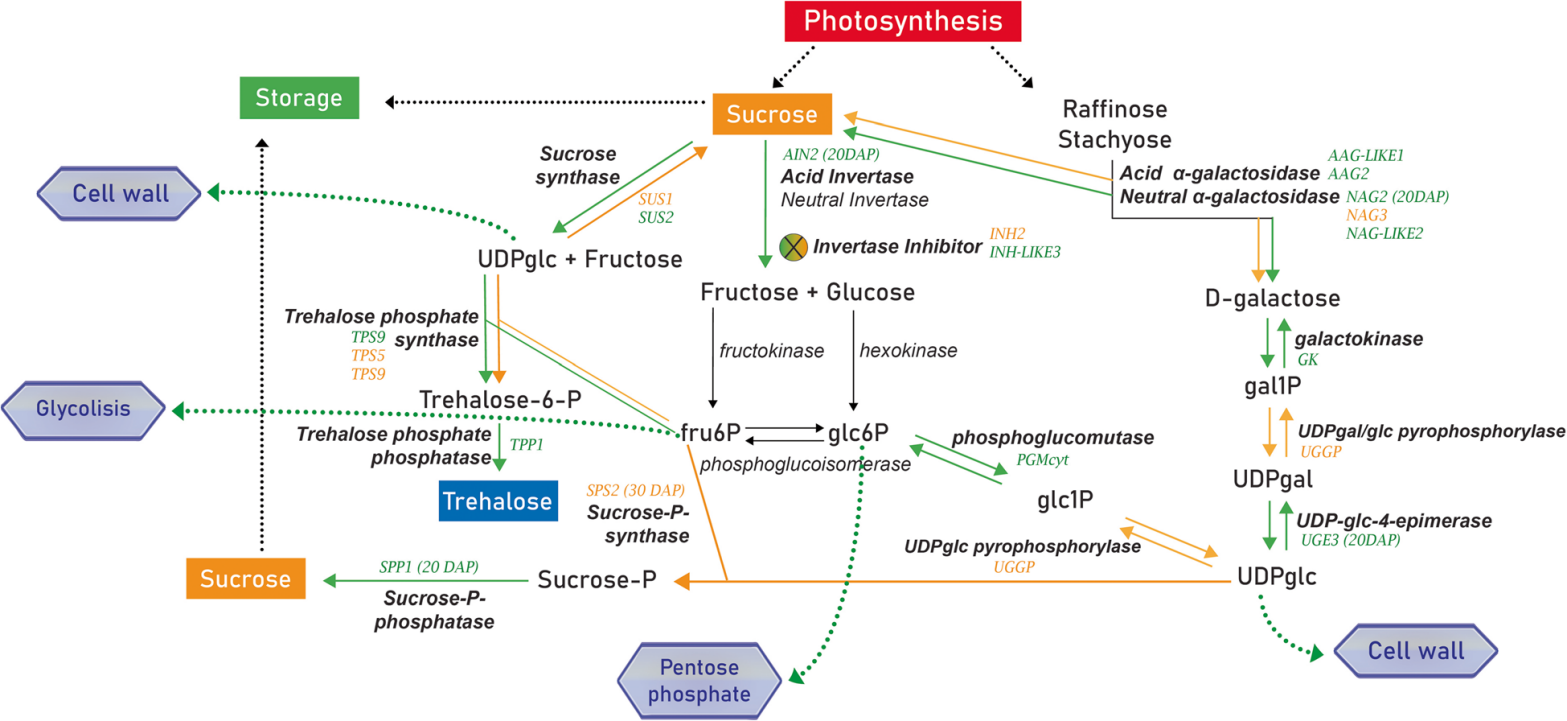
Differential expression of sugar metabolism related genes in the melon ripening process. The green arrows represent 10 DAP or 20 DAP fruits and the yellow arrows 30 DAP or 40 DAP fruits. The genes with burst of expression in the intermediate stages (20 DAP and 30 DAP) have the phase indicated in parentheses

Sugar metabolism is an important pathway related to the sweetness of fruits and it is the most attractive characteristic for consumers [51]. Furthermore, studies have reported that sugars may serve as important signals that modulate a wide range of processes in plant physiology including fruit maturation [39, 52, 53]. In our study, a total of 17 genes were DEs in the sucrose; amino and nucleotide sugar; and galactosidase pathways and 8 were evaluated in two additional development stages (20 and 30 DAP). Sucrose is the main sugar component that gives the sweet taste in melon and its high content at the mature stage could be used as a marker [6]. Only sucrose synthases and invertases are known enzymes responsible for sucrose cleavage [10]. The sucrose synthases convert sucrose to fructose and UDP glucose that is a reversible reaction [10]. In our study, two isoforms were DEs by RNA-seq analysis, the *CmSUS1* that was up-regulated in full-ripe fruit while *CmSUS2* had a burst of gene expression in 10 DAP fruit. In fruit maturation, there is a gradual expression increase of *CmSUS1* and decrease of *CmSUS2* in the ‘Yellow’ melon. In non-climacteric melons, ‘Hami’ [51] and ‘Piel del Sapo’ [6], the same expression profile was observed. In ‘Dulce’ climacteric melon, the *CmSUS1* was more expressed in young fruit, followed by near-silencing in mature fruit. *CmSUS2* showed low levels of expression throughout fruit development and the third sucrose synthase (*CmSUS3*) was DE being weakly expressed in the young fruit and increased in the maturing fruit [9]. Thus, it may be suggested that in non-climacteric melons, *CmSUS1* is mainly responsible for the synthesis of sucrose for storage in the vacuole, contributing to ripe fruit taste, while *CmSUS2* acts in an opposite way providing the substrate for energy production by sucrose catabolism during early development (Fig. 7). Also, the TPS and TPP have an important function in the sucrose synthase activities contributing to sucrose content in the fruit as previously described (Fig. 7).

Invertases produce glucose instead of UDP-glucose and fructose in a non-reversible reaction. Acid invertases have been attributed to vacuole localization while neutral invertases have generally been located in the cytosol, consistent with the optimal neutral pH activity and absence of glycosylation [9]. In the RNA-seq analysis, only the acid invertase (*CmAIN2*) was DE in non-climacteric ‘Yellow’ melon. Previous studies with ‘Piel del Sapo’ [6] and ‘Hami’ non-climacteric melon fruit [51] also showed only transcriptional activity of acid invertase 2 (*CmAIN2*). In ‘Dulce’ climacteric melons, four neutral invertase (*CmNIN1*, *CmNIN2*, *CmNIN3* and *CmNIN4*) were DE, as well as the acid invertase 2 (*CmAIN2*) [9]. The peak of *CmAIN2* expression occurs in the 20 DAP ‘Yellow’ melon fruits and consistently decreased in the following developmental stages (Fig. 7). Studies have demonstrated that acid and neutral invertase genes are highly expressed in young developing fruit, and subsequently declined substantially at the sucrose accumulation stage [6, 9, 16, 51]. This reduction of soluble acid invertase activity signals the metabolic transition from fruit growth to sucrose accumulation [3, 18]. The higher expression of neutral invertases in climacteric melon fruit suggests that cytoplasmatic sugar catabolism might be an additional source of energy, supporting the hypothesis that climacteric melon fruit spends more energy during fruit development, due to respiration, than non-climacteric ones. In the non-climacteric and climacteric melon comparison, studies demonstrated that the acid invertase gene (*CmAIN2*) was almost 10-fold higher in ‘Védrantais’ (climacteric) than in ‘Piel del Sapo’ (non-climacteric). The high activity of soluble acid invertase (*CmAIN2*) might limit the accumulation of sucrose during climacteric ripening and increase organic acids, such as malate, that impart a stale flavour to the fruit [3, 6, 9, 18]. Thus, the inhibition of *CmAIN2* can be a key process in the differences of sugar content and melon quality.

Invertase inhibitors are responsible for decreasing the activity of soluble acid invertases through post-translational regulation, reducing sugar consumed in respiration and regulating the accumulation of sucrose during melon development and ripening [6, 18]. Two invertase inhibitors (*CmINH2*; *CmINH-LIKE3*) were DE by RNA-seq analysis and are characterized by the presence of plant invertase/pectin methyltransferase inhibitor domain (Additional file 12: Figure S11). The invertase inhibitor 2 presented higher transcription level in full-ripe (40 DAP) than in youngest melon (10 DAP) (Fig. 7). On the other hand the putative invertase inhibitor 3 (*CmINH-LIKE3*) had high activity in the beginning of development and low expression in the following stages (Fig. 7). Hence, in the 20 DAP stage the inactivation of *CmAIN2* by *CmINH-LIKE3* protein interaction may be occurring. Subsequently, the *CmAIN2* expression rapidly decreases and the *CmINH-LIKE3* transcription is not necessary anymore. The *CmINH2* can have affinity with other invertases that have not been evaluated on the intermediate stages (20 DAP and 30 DAP). The putative *CmINH-LIKE3* expression was not reported in previous studies. The DE of *CmINH2* was also detected in climacteric ‘Dulce’ melon; however, it is more strongly expressed in the first stages of development decreasing during ripening [9]. One other DE isoform detected for this same variety was *CmINH1* that was expressed at high levels in the 30 DAP stage [9]. The results demonstrated different expression profiles for non-climacteric ‘Yellow’ melon and climacteric ‘Dulce’ melon, indicating recruitment of distinct INH isoforms in the regulation of the invertases or its activation in different stages of ripening. Moreover, two invertase inhibitors (cCL2226Contig1 and c15d_02-B02-M13R_c) are about 30 times higher in non-climacteric ‘Piel del Sapo’ melon than in climacteric ‘Védrantais’ melon [6]. These characteristics are in accordance with invertase expression differences.

The raffinose oligosaccharides (RFOs) synthesized by photosynthesis can be converted into sucrose and galactose by α-galactosidases in fruit tissues. In melon, α-galactosidase includes acid and neutral isoenzymes and in young fruit, both can be used to provide energy for the growth metabolism. In mature fruits, sucrose can be stored in the vacuole while galactose can be metabolized to sucrose [3, 6, 9]. In our study, three neutral α-galactosidases (*CmNAG2*, *CmNAGLIKE2*, *CmNAG3*) were DEs (Fig. 7). In the early development stages of ‘Yellow’ melon, there is an increase of the *CmNAG2* expression, that has the highest level in the 20 DAP stage and decreases in 30 and 40 DAP. Though the RNA-seq analyses demonstrated that in the 40 DAP stage the expression of this enzyme was higher than in 10 DAP, the log2 fold change was low (1.07). These differences might be due to individual variations. The *CmNAGLIKE2* also has high activity in the beginning of fruit development, while *CmNAG3* peaks in the 40 DAP stage. Previous studies showed no DE of neutral α-galactosidases in non-climacteric melon. In climacteric ‘Dulce’ melon the high activity transcriptional of *CmNAG2* was also detected in early stages and low activity of acid α-galactosidases in the maturation process [9]. We suggest that the sucrose produced in the catabolic reaction of *CmNAG2* and *CmNAGLIKE2* in young fruit is recruited in respiration by its conversion to hexoses, evidenced by the high activity of *CmAIN2* and *CmSUS2* in the same stages. The *CmNAG3* has a function in the sucrose accumulation in full-ripe melon.

Another important route of sucrose synthesis is by conversion of galactose-1P into glucose-1P by UDP-glucose epimerase (UGE) [3, 6, 9, 17]. In the present study, we observed the increase of *CmUGE3* expression in the initial development, followed by subsequent decrease until the full-ripe stage, implying a concomitant gene expression with *CmNAG2* (Fig. 7). The activity of both enzymes denotes high sucrose production in young fruits. In climacteric ‘Dulce’ melon, three UGEs were DE throughout fruit development (*CmUGE1*, *CmUGE2* and *CmUGE3*), but *CmUGE3* expression increased significantly during fruit maturation [9]. The *CmUGE3* gene expression or its enzyme activity were not reported in other non-climacteric melon in previous studies [6, 51]. Antagonistic profile of *CmUGE3* is observed in the two melon phenotypes that can be associated with differences in sugar accumulation.

Sucrose-P synthase (SPS) is considered the key gene for sucrose accumulation in fruit ripening [9, 16, 18]. This enzyme catalyzes the reversible transfer of a hexosyl group from UDP-glucose to D-fructose 6-phosphate to form UDP and D-sucrose-6-phosphate [54]. Only *CmSPS2* was DE in our study, which has an increase during fruit maturation, reaching the highest levels in colour change melons (30 DAP) (Fig. 7). This is in agreement with gene expression observed in ‘Hami’ non-climacteric melon [51]. In climacteric ‘Dulce’ melon, *CmSPS2* is weakly expressed during fruit development, but *CmSPS1* rapidly increased from 20 DAP, peaking at late developmental stages [9]. The gene expression of SPS was similar when comparing climacteric and non-climacteric melon, however different isoenzymes are responsible for syntheses D-sucrose-6-phosphate. The sucrose phosphate phosphatase (SPP) has complementary activity with SPS by conversion of sucrose-6-phosphate to sucrose [3, 9, 16, 18, 51]. In our study only *CmSPP1* was DE, having a moderate expression without pronounced differences in the first two stages but with evident decrease in the full-ripe fruit. In non-climacteric ‘Hami’ melon, the DE of sucrose-P phosphatase was not observed [51]. In climacteric ‘Dulce’ melon, the *CmSPP1* gene was weakly expressed with a slight increase in the 40 DAP stage [9].

## Conclusion

Considering the limited knowledge about molecular mechanisms that act in the ripening process in non-climacteric melon, studies that involve high-throughput analyses like RNA-seq are paramount to open new perspectives on this matter. Sucrose-cleaving enzymes perform essential mechanisms for the distribution and use of sucrose in fruits. Only sucrose synthase and invertase enzymes can cleave sucrose. *CmSUS2* and *CmAIN2* are up-regulated in the early development stages of the ‘Yellow’ melon (Fig. 7), indicating high hexose production, which in turn increases the respiration metabolism and the generation of hexose-based signals. Studies demonstrated that these signals are involved in development processes such as cell division [10]. In addition, the UDP glucose product of sucrose synthase has been implicated in the formation of diverse cell wall polysaccharides [55]. The sucrose substrate for these enzymes is provided by *CmNAG2*, *CmNAGLIKE2* and *CmUGE3* transcriptional activity that is high in the same stages. The new putative invertase inhibitor *CmINH-LIKE3* (exclusively expressed in ‘Yellow’ melon) decreases invertase activity in young non-climacteric fruit (Fig. 7), suggesting its importance during non-climacteric melon fruit development, sucrose accumulation and organic acid content. SPP1 has the highest expression in 20 DAP fruit and *CmSPS2* in 30 DAP, both enzymes have a complementary role in sucrose biosynthesis in the intermediate stages (Fig. 7). Finally, *CmNAG3* and *CmSUS1* have a crucial function in sucrose accumulation in the late stages of fruit development (Fig. 7). Also, the higher expression of *CmTPP* in the early stages increases the trehalose-6-phosphate conversion to trehalose preventing sucrose synthase inhibition *(CmSUS2*) (Fig. 7). In contrast, trehalose-6-phosphate accumulation by TPS activity inhibits sucrose cleavage in full-ripe melons (Fig. 7).

Many genes within hormone pathways showed differential expression detected by RNA-seq analysis. The hormones also play an essential function in fruit ripening, but the mechanisms are complex and poorly understood. In our study, for IAA, JA, GA, CYT, ABA and BRs signal transduction, most genes are more expressed in young than full-ripe fruit. This characteristic is observed in previous studies [29, 34, 35, 56]. In mature fruit, the auxin response factor, responsive auxin, 9-cis-epoxycarotenoid dioxygenase and receptor of ethylene 1 with the highest transcriptional activity were detected and are interesting genes for further melon ripening studies. Furthermore, several studies have demonstrated the integration of sucrose and hormonal pathways including auxin and abscisic acid, as well as epigenetic control (microRNAs and chromosomal modification) acting on fruit development.

This is the first study conducted for non-climacteric ‘Yellow’ Brazilian commercial melon and the results on sugar metabolism and related pathways during development and ripening contribute to new perspectives in management practices and molecular tools to improve fruit quality.

## Methods

### Plant material

Non-climacteric melon fruit of a ‘Yellow’ commercial genotype *(Cucumis melo, Inodorus* group) was cultivated and provided in the different ripening stages by Itaueira Agropecuária SA company in São Paulo (Brazil). Fruit was manually pollinated, and three biological replicates were harvested at different development stages: 10 days after pollination (DAP), 20 DAP, 30 DAP and 40 DAP. For each sampling time, flesh mesocarp was collected, immediately frozen in liquid nitrogen and stored at − 80 °C until analysis.

### Colour, SS (soluble solids) and pH measurement

Peel and pulp colour at different ripening stages were measured using a Minolta CR400 colorimeter. The CIE (*Commission Internationale de l’Eclairage)* L* (lightness), a* (green/red coordinate), b* (blue/yellow coordinate) colour scale was adopted. The angle Hue was calculated by the equation $h{}^{\circ}={\mathit{\tan}}^{-1}\left(\frac{b\ast }{a\ast}\right)$ if a* > 0 and b* > 0 or by equation $h{}^{\circ}=180+{\mathit{\tan}}^{-1}\left(\frac{b\ast }{a\ast}\right)$ if a* < 0 or b* > 0 [20]. The soluble solids content (SS° Brix) and the pH were measured using digital refractometer and automatic pH matter respectively. Three measurements were made for each fruit and a mean was obtained.

### RNA extraction

Total RNA was extracted in biological triplicate (different fruit) of four development stages using the sodium perchlorate method as described for melon by Campos et al. (2017) [57]. The RNA quality and quantity were determined using Nanovue™ spectrophotometer and 1% agarose gel electrophoresis. Only RNAs that presented A260/A280 ratio ~ 2.0, A260/A230 ratio ~ 1.80 and no discernible degradation were used for RNA-seq and qPCR analyses.

### Preparation of cDNA libraries and RNA-seq

The cDNA library preparation for RNA-seq analyses was performed to 10 DAP and 40 DAP fruits. Sample RNA quality and concentration for RNA-seq were assessed with the Agilent 2100 Bioanalyzer (Thermo Scientific). Messenger RNA (mRNA) was isolated using the Dynabeads mRNA Direct Micro kit (Life Technologies). Single-end libraries were prepared with the Ion Total RNA-Seq Kit v2, barcoded with the PI™ Chip Kit v3 at the Federal University of Paraná (Curitiba, BR). After pooling into two-sample groups, the libraries were sequenced (five technical replicas) on an Ion Torrent™ (Life Technologies™) using the PI Template 200 bp v3 and Ion PI Sequencing 200 Kit v3. A total of ~ 27 million reads were obtained for each sample. The raw sequencing data has been deposited in the NCBI sequence read archive (SRA) under the accession number SRP230494 (https://trace.ncbi.nlm.nih.gov/Traces/sra/?study=SRP230494) .

### Gene expression analysis of RNA-Seq data

RNA-Seq reads from each biological replicate (2 per 10 DAP and 3 per 40 DAP) were filtered and submitted to adaptor trimming using fastx-toolkits (hannonlab.cshl.edu/fastx_toolkit/) and cutadpt [58] respectively. Reads showing ≥80% of sequenced bases with Phred scores over 20 and more than 50 bp in length were selected. The melon genome (*Cucumis melo* version v3.6.1) and annotation (gff3 file) provided at https://www.melonomics.net were used as a reference in differential expression analysis. Transcriptome mapping was achieved by using the software Bowtie2 aligner [24]. Gene counts were calculated using featureCounts and only reads with overlapping in a single gene were considered for RNA-seq analysis [59]. Differential expression analyses were carried out applying a statistical test evaluating the negative binomial distribution provided in the R package DeSeq2 [60]. For each gene, the padj ≤0.05 was considered as the significant threshold. The hierarchical clustering was performed to sample clustering analysis and to evaluate the profile of the top 50 DE genes using the gplots package and heatmap.2 function available in R.

### Gene ontology (GO) and Kyoto encyclopedia of genes and genomes (KEGG)

Gene ontology term enrichment analysis of DE genes was performed using http://cucurbitgenomics.org/goenrich software (dataset melon DHL92 v3.61), with FDR (false discovery rate) adjusted *p*-value < 0.05. KEGG pathway enrichment analysis was carried out using DAVID according to the default actions [26]. The pathways of differentially expressed genes were visualized using the ‘Pathview’ software based on the KO-gene-assignment file and fold change value for each gene under pairwise comparisons [61]. The degree of log2 fold changes was highlighted in different colours.

### Protein-protein interaction (PPI) network construction and modules mining

Search Tool for the Retrieval of Interacting Genes/Proteins (STRING) is a database of protein-protein interaction [25]. This database contains direct and physically related interactions between known and predicted proteins and genes. The sources are mainly from (a) experimentally determined (b) text mining in scientific articles and other databases, (c) gene-neighbourhood, (d) gene fusions, (e) co-expression, (f) gene co-occurrence, and (g) protein homology. The system uses a scoring mechanism to give a certain weight to the results and finally gives a comprehensive high throughput analyses [62]. For this analysis we setting the minimum required interaction score 0.400, none max number of interactors and all interaction sources were selected. The Cytoscape software [63] was used to analyze protein-protein interaction (PPI) generated by STRING. In this study, we set as input the genes 10 DAP-biased fruit separately from genes of 40 DAP-biased fruit. We selected the proteins interactions more relationship with sucrose metabolism from the general network.

### Quantitative reverse transcription PCR analysis (RT-qPCR)

To validate the accuracy of transcriptome profiling, the gene expression of eight transcripts related to the sugar pathway was evaluated by quantitative reverse transcription PCR. Gene specific primers were designed using the ‘PrimerBlast’ database (https://www.ncbi.nlm.nih.gov/tools/primer-blast/) (Additional file 3: Table S3). The RPS15 and RPL genes were used with internal control according to Kong et al. (2014) [64]. Also, in these analyses all fruit development stages were considered (10 DAP, 20 DAP, 30 DAP and 40 DAP). Total RNA was treated with the TURBO™ DNase kit (Invitrogen) to remove genomic DNA residues from the extraction and it was submitted to cDNA conversion by Maxima H minus First Strand cDNA Synthesis kit (Thermo Scientific) following the manufacturer’s instructions.

RT-qPCR was performed on a LightCycler® Nano platform (Roche Diagnostics GmbH, Mannheim, Germany) using 100 ng of cDNA in a reaction containing 1 μL of forward and reverse primer (10 μM), 10 μL FastStart Essential DNA Green Master 2X (Roche), in a final volume of 20 μL. The amplification conditions were performed at 94 °C for 10 min and then cycled at 95 °C for 15 s, 55–60 °C for 20s, 72 °C for 20s for 45 cycles. A melting curve analysis (60 °C to 99 °C) was performed after the thermal profile to ensure specificity in the amplification. Each assay was performed in triplicates. Relative gene expression analysis was performed using the 2^-∆∆Ct^ method according to Livak and Schmittgen (2001) [65]. Data were converted to a log2 fold change scale to make the data comparable with the RNA-seq results. Pearson’s correlation distance was calculated across 10 DAP and 40 DAP developmental stages.

### RT-qPCR statistical analysis

The R was used for the statistical analyses. Normality was statistically assessed by the Shapiro–Wilk test [66]. Values that were not normally distributed were transformed by the Box–Cox method [67]. Significant differences among means were determined with the ANOVA (*P* ≤ 0.05) and Tukey’s test (P ≤ 0.05). It was not possible to normalize the sucrose synthase 1 (*CmSUS1*) and sucrose synthase 2 (*CmSUS2*) genes in RT-qPCR, and the Kruskal-Wallis & Wilcoxon tests (P ≤ 0.05) were applied in this case.

## Supplementary information

**Additional file 1: Table S1.** Count of number of reads per gene obtained by featureCounts software. Only reads with overlapping in a sigle gene were considered for RNA-seq analysis; **Table S2.** RNA-seq data analysis (diferential expression and statistical test).

**Additional file 2: Figure S1.** PlotMA (Deseq2 R package) shows the log2 fold changes of young fruits (positive values) and full-ripe fruit (negative values) over the mean of normalized counts for all the samples. Points in red are genes that have significant differential expression (adjusted *p*-value ≤0.05). Points that fall out of the window are plotted as open triangles pointing either up or down.

**Additional File 3: Table S3.** Target genes and reference genes used in RT-qPCR analysis; **Figure S2.** Pearson’s correlation between the 10 DAP and 40 DAP development stage. The expression ratio for RNA-seq and RT-qPCR analysis are represented by log2 fold change.

**Additional file 4: Figure S3.** Heatmap of the sample-to-sample distances that gives an overview over similarities and dissimilarities between samples (V is 10 DAP fruit and M is 40 DAP fruit). Dark blue shade indicates higher levels of similarity and light blue indicates higher levels of dissimilarities.

**Additional file 5: Table S4.** Gene ontology enrichment analysis of young and mature melon DE genes. This analysis was performed using FDR (false discovery rate) adjusted p-value < 0.05 on DE genes (http://cucurbitgenomics.org/goenrich).

**Additional file 6: Table S5.** The top 50 DE genes between young (10 DPA) and mature (40 DAP) fruit samples; **Figure S4.** Hierarchical clustering analyses of DE top 50 genes between young (10 DPA) and mature (40 DAP) fruit samples. The log2 fold change values were converted by rlog (regularized logarithm) function in Deseq2. Each line represents one gene and the rows are the samples. The colour bar represents the rlog values and ranges from blue (low expression) to red (high expression).

**Additional file 7: Table S6.** Differential expressed genes present on KEGG enrichment pathways (Fisher exact test ≤0.05).

**Additional file 8: Figures S5, S6 and Tables S7, S8.** Figures represent protein–protein interaction network of young (Figure S4) and mature melon (Figure S5) fruit generated by STRING and Cytoscape analyses. The tables represent the characteristics of the network interaction.

**Additional File 9: Tables S9, S10, S11, S12.** Characteristics of PPI network interaction of sugar and associated pathways.

**Additional File 10: Figures S7, S8, S9.** KEGG (Kyoto Encyclopedia of Genes and Genomes) analyses using Pathview software (https://pathview.uncc.edu/) of “starch and sucrose metabolism”, “galactose metabolism” and “amino sugar and nucleotide sugar metabolism”. The colour bar represents de log2 fold change of the maturation process and ranges from green (up-regulated genes in 40 DAP fruit) to red (up-regulated genes in 10 DAP fruit). The blue letters are enzyme short names described in the KEGG pathway and the purple are enzyme short names that were described in the literature associated with the sugar pathway [6, 9]. There are protein isoforms that act in the same metabolic route and the information of all log2 fold change were included.

**Additional File 11: Figure S10.** Graphics of the normalized gene counts obtained by RNA-seq results (plotCounts function of DESeq2 analysis – differential gene expression analysis based on negative binomial distribution).

**Additional File 12: Figure S11.** Alignment of protein isoforms related to sugar metabolism or associated pathways. The domains were detected by Pfam database (https://pfam.xfam.org/) and the amino acid sequences were aligned by the MUSCLE algorithm.

## Data Availability

The raw sequencing data has been deposited in NCBI sequence read archive (SRA) under the accession number SRP230494 (https://trace.ncbi.nlm.nih.gov/Traces/sra/?study=SRP230494). All other data analyzed in the present study are included in this article and its additional files.

## Abbreviations

RNA: Ribonucleic acid
RNA-seq: RNA sequencing
PCR: Polymerase chain reaction
RT-qPCR: Quantitative reverse transcription PCR
DAP: Days after pollination
DNA: Deoxyribonucleic acid
DE: Differentially expressed
RFO: Raffinose family oligosaccharide
Glc: Glucose
NAG: Neutral α-galactosidase
AAG: Acid α-galactosidase
GK: Galactokinase
Gal1P: Galactose 1-phosphate
Glc1P: Glucose 1-phosphate
UGGP: UDP-gal/glc pyrophosphorylase
SPS: Sucrose-phosphate synthase
SPP: Sucrose-phosphate phosphatase
CIN: Cell wall invertase
HXK: Hexokinase
FK: Fructokinase
SUS: Sucrose synthase
UDP-glc: UDP glucose
NIN: Neutral invertase
AIN: Acid invertase
INH: Invertase inhibitor
NGS: Next generation sequencing
SS: Soluble solids
CIE: Commission Internationale de l’Eclairage
bp: Base pair
DEG: Differentially; expressed genes
GO: Gene ontology
MF: Molecular function
CC: Cellular component
BP: Biological process
ETH: Ethylene
ABA: Abscisic acid
BR: Brassinosteroids
IAA: Auxin
CYT: Cytokinins
GA: Gibberellin
JA: Jasmonic acid
UDP: Uridine diphosphate
T6P: Trehalose-6-phosphate
TPP: Trehalose phosphate phosphatase
miRNA: micro RNA
TPS: Trehalose phosphate synthase
UGE: UDP-glucose epimerase
cDNA: Complementary DNA
mRNA: Messenger RNA
BR: Brazil
NCBI: National Center for Biotechnology Information
KEGG: Kyoto Encyclopedia of Genes and Genomes
PPI: Protein-protein interaction
STRING: Search Tool for the Retrieval of Interacting Genes/Proteins
