# Supplementary figures and images for: Transcriptome profiling of non-climacteric ‘yellow’ melon during ripening: insights on sugar metabolism
Source: BMC Genomics. 2020 Mar 30;21:262. doi: 10.1186/s12864-020-6667-0 (PMC7106763; doi:10.1186/s12864-020-6667-0)

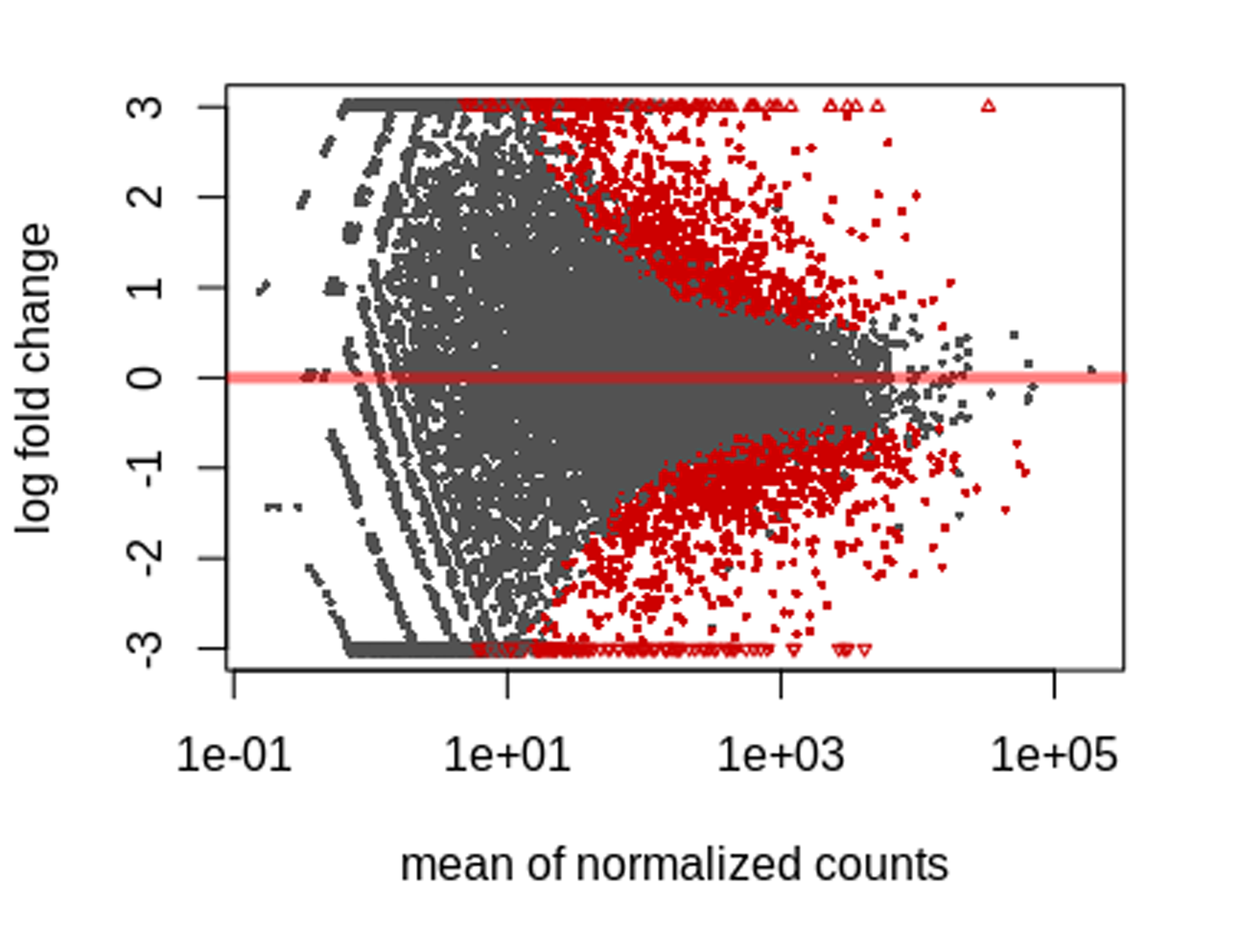

Supplement: Supplementary file 2 — Additional file 2: Figure S1. PlotMA (Deseq2 R package) shows the log2 fold changes of young fruits (positive values) and full-ripe fruit (negative values) over the mean of normalized counts for all the samples. Points in red are genes that have significant differential expression (adjusted p-value ≤0.05). Points that fall out of the window are plotted as open triangles pointing either up or down. [file 12864_2020_6667_MOESM2_ESM.tiff]

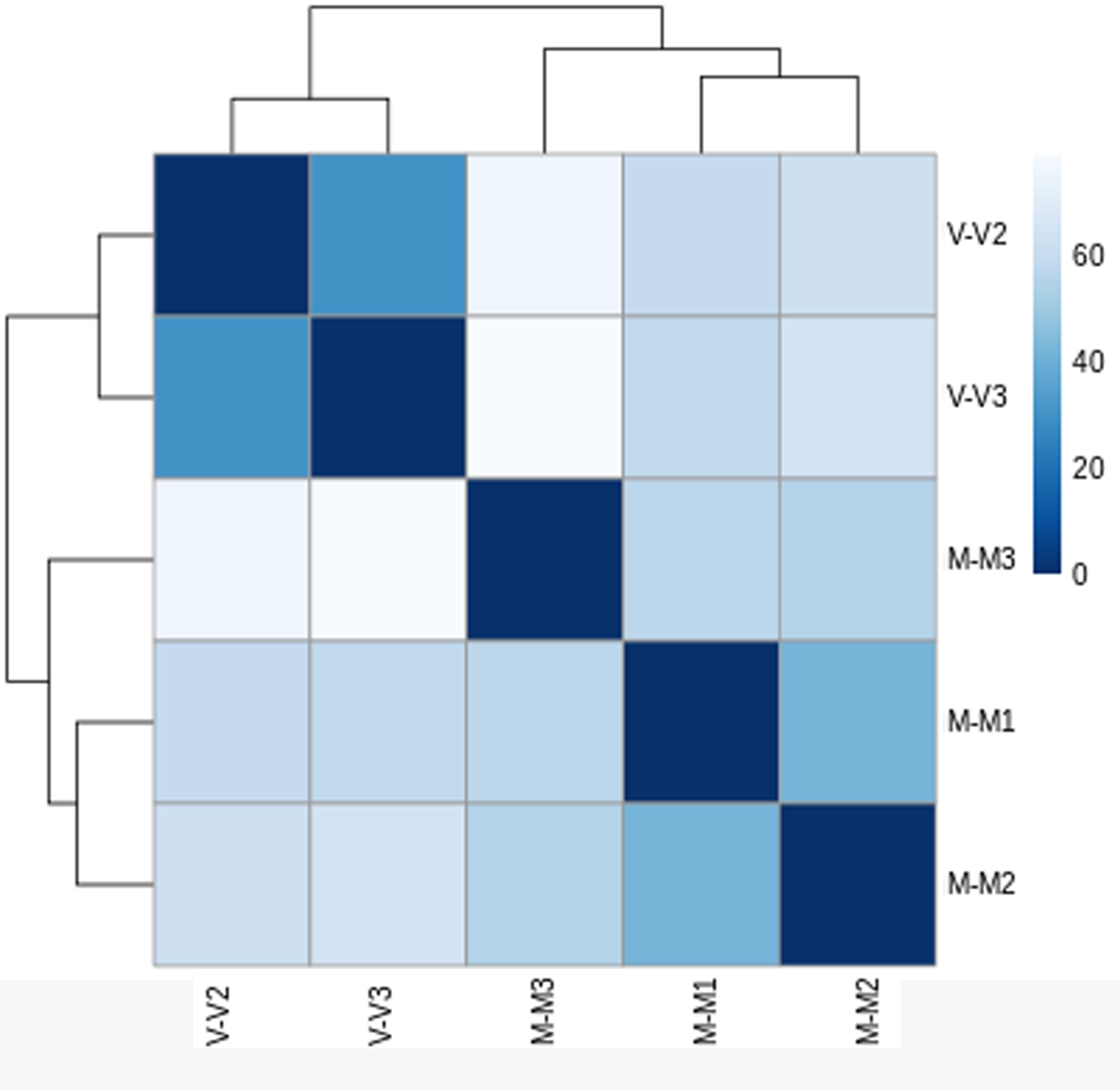

Supplement: Supplementary file 4 — Additional file 4: Figure S3. Heatmap of the sample-to-sample distances that gives an overview over similarities and dissimilarities between samples (V is 10 DAP fruit and M is 40 DAP fruit). Dark blue shade indicates higher levels of similarity and light blue indicates higher levels of dissimilarities. [file 12864_2020_6667_MOESM4_ESM.tiff]
