# Supplementary material for: Transcriptome profiling of non-climacteric ‘yellow’ melon during ripening: insights on sugar metabolism
Source: BMC Genomics. 2020 Mar 30;21:262. doi: 10.1186/s12864-020-6667-0 (PMC7106763; doi:10.1186/s12864-020-6667-0)

Table S3 Target genes and reference genes used in RT-qPCR analysis.

Gene	Short Form	Primer 5' – 3'	Product size (bp)	Temperature (°C)
Neutral alpha-galactosidase 2	*CmNAG2*	F – TTCCTGTGGTATCGATGGCG<br>R – ATGAATGGTGTGGGAGGCAG	258	62ºC
Acid Invertase 2	*CmAIN2*	F – CTCGATCTGTCAAAAAGCAGC<br>R – TTCCCCAAACGCCCCTAAAG	213	62ºC
New putative invertase inhibitor	*CmINH-Like3*	F' CTCGCATTTTCCTTCGTTGT<br>R' AAAGGTCCGCCGTGTCGCT	164	62ºC
UDPglc epimerase 3	*CmUGE3*	F – CTGTTGGGGAGAGTGTTGCT<br>R – GCTTCGTCCGACCATAAGGA	203	62ºC
Sucrose synthase 1	*CmSUS1*	F - AACCGTGTTCATAGCCTCCG<br>R – TCAGGACTTCCCCAAATGCC	195	64ºC
Sucrose synthase 2	*CmSUS2*	F - TCCAGATGCGAAGGGGACTA<br>R – ATCCGGCTTTGCTTCCATGA	204	62ºC
Sucrose-P-Synthase 1	*CmSPS2*	F - AGTTCGTTTCTTCGTTTGGCT<br>R – TTGGCGCTTCTTTTGTGATGG	198	62ºC
Sucrose-P-Phosphatase 1	*CmSPP1*	F' TATCGTCACTCTTCTCCAC<br>R' CATGAGACGTGCTGAAGCG	182	60ºC
Cytosolic 40s Ribosomal protein S15	*CmRPS15*	F - GAAGCTGCGTAAAGCGAAAC<br>R – GGTCTTTCCATTGTAAACTCCAA	132	62ºC
60s Ribosomal protein L	*CmRPL*	F - CGACAATACTGGAGCCAAGAA<br>R – CATCACCATATCTCCCACACAA	100	62ºC

Figure S2 Pearson's correlation between 10 DAP and 40 DAP development stage. The expression ratio for RNA-seq and RT-qPCR analysis are represented by log2 FoldChange.

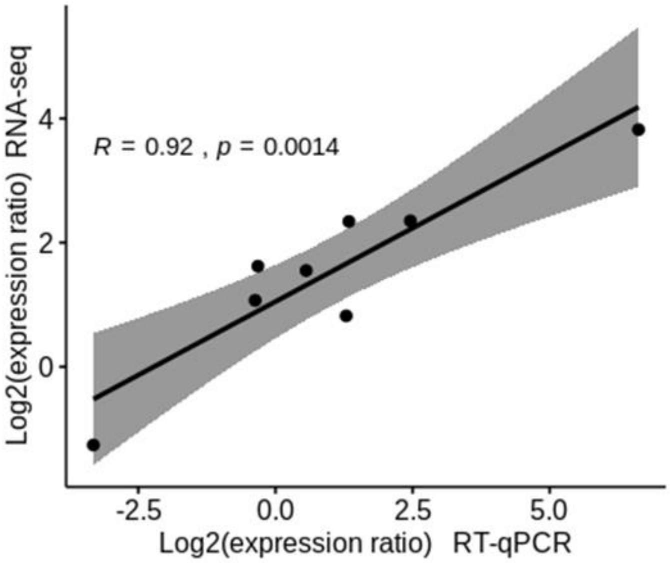

Supplement: Supplementary file 3 — Additional File 3: Table S3. Target genes and reference genes used in RT-qPCR analysis; Figure S2. Pearson’s correlation between the 10 DAP and 40 DAP development stage. The expression ratio for RNA-seq and RT-qPCR analysis are represented by log2 fold change. [file 12864_2020_6667_MOESM3_ESM.pdf]
