# Supplementary material for: Transcriptome profiling of non-climacteric ‘yellow’ melon during ripening: insights on sugar metabolism
Source: BMC Genomics. 2020 Mar 30;21:262. doi: 10.1186/s12864-020-6667-0 (PMC7106763; doi:10.1186/s12864-020-6667-0)
Supplement: Supplementary file 6 — Additional file 6: Table S5. The top 50 DE genes between young (10 DPA) and mature (40 DAP) fruit samples; Figure S4. Hierarchical clustering analyses of DE top 50 genes between young (10 DPA) and mature (40 DAP) fruit samples. The log2 fold change values were converted by rlog (regularized logarithm) function in Deseq2. Each line represents one gene and the rows are the samples. The colour bar represents the rlog values and ranges from blue (low expression) to red (high expression). [file 12864_2020_6667_MOESM6_ESM.pdf]

Table S5: The top 50 DE genes between young (10 DPA) and mature (40 DAP) fruits samples.

Melonomics ID (v4.0)	RefSeq	Description	Pathway (KEGG)*	Log2 FoldChange	padj
1 MELO3C010353.2	XP_008444009.1	protein NRT1/ PTR FAMILY 6.2-like	-	3.455867	4.106e-34
2 MELO3C017480.2	NP_001284442.1	Xyloglucan endotransglucosylase/hydrolase	cmo04075	3.967615	5.3832e-32
3 MELO3C016259.2	XP_016903401.1	Bidirectional sugar transporter SWEET	cmo00001	2.88248	1.1448e-09
4 MELO3C027112.2	XP_008453627.1	Protein PHLOEM PROTEIN 2-LIKE A1	-	3.2693	3.0634e-10
5 MELO3C004351.2	XP_008446316.1	probable pectate lyase P59	cmo00040	4.34667	1.79131e-49
6 MELO3C010210.2	XP_008443031.1	Unknown protein	-	4.45075	2.3567e-35
7 MELO3C022828.2	XP_008460506.1	Glutamine synthetase	cmo00220/cmo00250/cmo00630/ cmo00910/ cmo01230	4.541077	3.6177e-42
8 MELO3C012053.2	XP_016903282.1	thioredoxin H-type	cmo00001	4.937584	8.70463e-35
9 MELO3C020823.2	XP_016901630.1	Heparan-alpha-glucosaminide N-acetyltransferase	-	3.550465	1.8113e-29
10 MELO3C007006.2	XP_008459054.1	Protein PHLOEM PROTEIN 2-LIKE A9	-	3.986353	2.7978e-37
11 MELO3C015868.2	XP_008451400.1	2OG-Fe(II) oxygenase family oxidoreductase	-	3.909021	4.6833e-31
12 MELO3C005442.2	XP_008453829.1	BnaC09g40830D protein	-	4.078132	8.9163e-13
13 MELO3C024920.2	XP_008462987.1	zinc transporter 11	cmo00001	5.20622	4.8623e-63
14 MELO3C025265.2	XP_008447666.1	glycogen synthase kinase-3 homolog MsK-3	cmo00001	-4.17374	1.4152e-18
15 MELO3C018536.2	XP_008455060.1	pathogenesis-related protein 1-like	cmo04016/cmo04075/cmo04626	-4.71659	1.2173e-16
16 MELO3C029935.2	XP_008451399.1	mitochondrial ubiquitin ligase activator of NFKB 1	cmo00001	-3.90787	3.8504e-15
17 MELO3C001321.2	XP_016901186.1	ubiquitin carboxyl-terminal hydrolase 25	cmo00001	-5.61825	2.0827e-11
18 MELO3C027687.2	XP_008459469.1	Histone-lysine N-methyltransferase ATXR3	-	-7.21777	1.3361e-11
19 MELO3C024834.2	XP_008442759.1	protein DMR6-LIKE OXYGENASE 2-like	-	-3.42299	0.0043
20 MELO3C027648.2	XP_008452461.1	Translation initiation factor IF-1, chloroplastic	-	-3.53993	0.0042
21 MELO3C000330.2	XP_008444202.1	DNA-directed RNA polymerase subunit beta"	cmo00001	-4.62797	0.0009
22 MELO3C025247.2	YP_004841763.1	Photosystem II 32 kDa protein	-	-3.18283	0.0469
23 MELO3C018627.2	XP_008455196.1	7-deoxyloganetin glucosyltransferase-like	-	4.636456	1.36143e-21
24 MELO3C016297.2	XP_016902861.1	Nucleoside diphosphate	cmo00001	4.840522	4.4364

			kinase			e-27
25	MELO3C025456.2	XP_008446238.1	WAT1-related protein	-	5.17524	4.9317 e-28
26	MELO3C033058.2	XP_008443490.1	WAT1-related protein	-	4.72164	0.0490
27	MELO3C029341.2	XP_008450882.1	Harbinger transposase-derived nuclease	cmo00001	3.968895	7.2539 e-09
28	MELO3C021426.2	XP_008442833.1	Protein TERMINAL FLOWER 1	cmo00001	6.645424	0.0006
29	MELO3C021350.2	XP_008455946.1	monothiol glutaredoxin-S2-like	-	7.382433	1.1348 e-34
30	MELO3C018509.2	XP_008451903.1	Glutamate racemase	-	7.972957	0.0123 2
31	MELO3C015214.2	XP_008450452.1	beta-glucosidase 24	cmo00460/cmo00500/ cmo00940/cmo01110	4.527561	0.0006
32	MELO3C018593.2	XP_008447193.1	Cysteine proteinase inhibitor	-	3.978864	1.0020 e-12
33	MELO3C008672.2	XP_008441807.1	Indole-3-acetic acid-amido synthetase GH3.3	cmo00001	3.942719	1.7872 e-19
34	MELO3C015257.2	XP_008450545.1	nicotianamine synthase-like	-	5.290715	0.0231 2
35	MELO3C033658.2	XP_008446132.1	protein GLE1	cmo00001	4.827639	0.0002
36	MELO3C020271.2	XP_008443791.1	major latex protein 15-like	cmo00001	6.545427	1.4251 e-50
37	MELO3C022518.2	XP_008460146.1	gibberellin 20 oxidase 1-like	cmo00270/ cmo01110	9.98387	2.7101 e-12
38	MELO3C018880.2	XP_008455506.1	Glycine-rich protein	-	10.05126	0.0009
39	MELO3C003375.2	XP_008465934.1	Two-component response regulator-like protein APRR2	-	3.390193	1.6566 e-27
40	MELO3C009210.2	XP_008464642.1	Class I heat shock protein	cmo00001	3.549776	3.2868 2e-41
41	MELO3C014821.2	XP_008450029.1	Cystinosin-like protein	cmo00001	3.769818	1.5965 e-17
42	MELO3C025101.2	XP_008463167.1	Sucrose synthase 2	cmo00500	3.827967	1.4299 e-41
43	MELO3C010875.2	XP_008445562.1	Calcium-binding EF-hand	-	3.241976	2.2164 e-32
44	MELO3C018882.2	XP_008456303.1	Unknown protein	-	2.890856	2.6924 e-13
45	MELO3C003132.2	XP_008442554.1	Metallothionein-like protein 4A	-	4.568294	1.1824 e-44
46	MELO3C029417.2	XP_008459177.1	Unknown protein	-	-5.10999	3.0834 e-36
47	MELO3C014045.2	XP_016900654.1	(-)-germacrene D synthase-like	cmo00909	-5.99705	5.9103 e-72
48	MELO3C031475.2	XP_008445976.1	Unknown protein	cmo00001	-5.20179	1.1984 e-08
49	MELO3C003917.2	XP_008450536.1	Heat shock 70 kDa protein	cmo03040/ cmo04141/cmo04144	-3.04529	3.3153 e-32
50	MELO3C019470.2	XP_008462393.1	glutelin type-A 2-like	-	-3.32639	1.4299 e-41

*cmo00001: Brite Hierarchies; cmo00040: Pentose and glucuronate interconversions; cmo00220: Arginine biosynthesis; cmo00250:Alanine, aspartate and glutamate metabolism; cmo00270: Cysteine and methionine metabolism; cmo00460: Cyanoamino acid metabolism; cmo00500: Starch and sucrose metabolism; cmo00630: Glyoxylate and dicarboxylate metabolism; cmo00910:Nitrogen metabolism;

cmo00940: Phenylpropanoid biosynthesis; cmo00909: Sesquiterpenoid and triterpenoid biosynthesiscmo; 01110: Biosynthesis of secondary metabolites; cmo01230: Biosynthesis of amino acids; cmo03040: Spliceosomecmo; cmo04141: Protein processing in endoplasmic reticulum; cmo04144: Endocytosis; 04075: Plant hormone signal transduction;

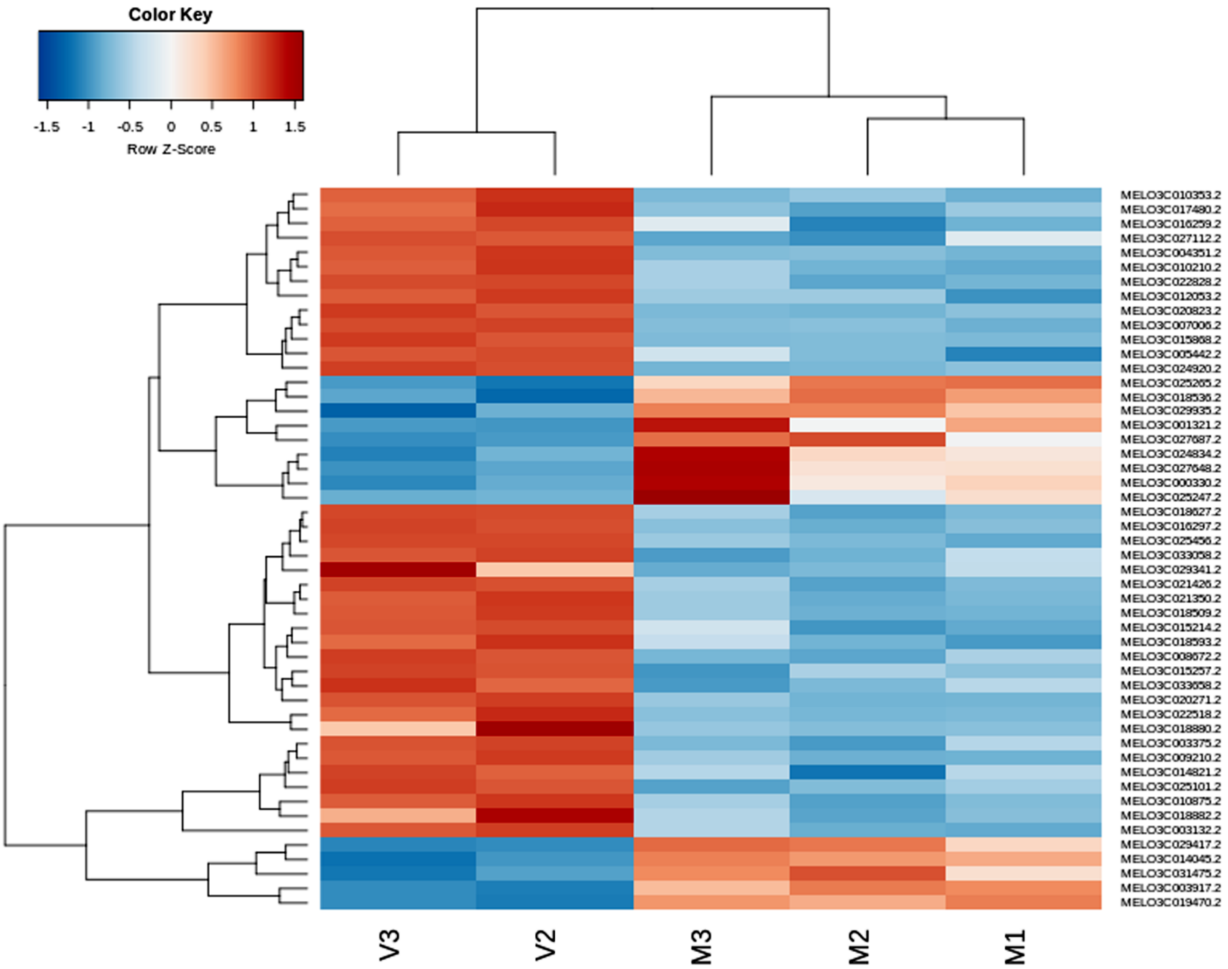

Figure S4 Hierarchical clustering analyses of DE top 50 genes between young (10 DPA) and mature (40 DAP) fruits samples. The log2 fold change values were converted by rlog (regularized logarithm) function in Deseq2. Each lines represents one gene and the rows the samples. The color bar represents the rlog values and ranges from blue (low gene expression) to red (high gene expression).
