# Supplementary material for: Transcriptome profiling of non-climacteric ‘yellow’ melon during ripening: insights on sugar metabolism
Source: BMC Genomics. 2020 Mar 30;21:262. doi: 10.1186/s12864-020-6667-0 (PMC7106763; doi:10.1186/s12864-020-6667-0)
Supplement: Supplementary file 7 — Additional file 7: Table S6. Differential expressed genes present on KEGG enrichment pathways (Fisher exact test ≤0.05). [file 12864_2020_6667_MOESM7_ESM.pdf]

Table S6: Differential expressed genes present on KEGG enrichment pathways (Ficher exact test ≤ 0.05).

10 DAP fruit

1) Plant hormone signal transduction

ID	Gene Name	Species	Log2 FoldChange
	**Gibberilin**		
103502508/XP_008464685.1	transcription factor PIF4-like(LOC103502508)	Cucumis melo	2.9819
103496794 /XP_008457015.1	DELLA protein GAI-like(LOC103496794)	Cucumis melo	2.0955
	**Auxin**		
103493078/XP_008451925.1	auxin response factor 18-like(LOC103493078)	Cucumis melo	1.3779
103490323/XP_008448013.1	auxin-induced protein 15A-like(LOC103490323)	Cucumis melo	6.4446
103489925/XP_008447319.1	auxin-induced protein 22A-like(LOC103489925)	Cucumis melo	1.2787
103499752/XP_008461049.1	auxin-induced protein IAA6(LOC103499752)	Cucumis melo	1.1910
103489837/XP_008447371.1	auxin-responsive protein IAA11(LOC103489837)	Cucumis melo	2.1070
103499461/XP_008460695.1 /XP_008460694.1	auxin-responsive protein IAA13(LOC103499461)	Cucumis melo	2.6297
103488149/XP_008444965.1	auxin-responsive protein IAA9-like(LOC103488149)	Cucumis melo	1.6545
103493400/XP_008452341.1	indole-3-acetic acid-amido synthetase GH3.6(LOC103493400)	Cucumis melo	5.7328
103485860/XP_008441807.1	indole-3-acetic acid-amido synthetase GH3.5-like(LOC103485860)	Cucumis melo	3.9427
103490488/XP_008448232.1	indole-3-acetic acid-amido synthetase GH3.6-like(LOC103490488)	Cucumis melo	2.5520
	**Acid Jasmonic**		
103490781/XP_008448683.1	transcription factor MYC2-like(LOC103490781)	Cucumis melo	1.9132
103501422/XP_008463217.1	coronatine-insensitive protein 1(LOC103501422)	Cucumis melo	0.6774
	**Brassinosteroid**		
103495262/XP_008454979.1	cyclin-D3-3(LOC103495262)	Cucumis melo	0.8344
103487865/XP_008444594.1	cyclin-D3-3-like(LOC103487865)	Cucumis melo	2.6610
103502635/XP_008464872.1	probable serine/threonine-protein kinase At4g35230(LOC103502635)	Cucumis melo	0.9627
103482744/XP_008437265.1	probable serine/threonine-protein kinase At5g41260(LOC103482744)	Cucumis melo	0.8628

	**Cytokinine**		
103487743/XP_008444405.1	two-component response regulator ARR11(LOC103487743)	Cucumis melo	1.8855
103488557/XP_016899933.1	histidine-containing phosphotransfer protein 4-like(LOC103488557)	Cucumis melo	2.6155

	**Abscisic acid**		
103488507/XP_008445513.1	protein phosphatase 2C 77-like(LOC103488507)	Cucumis melo	1.5051

Obs.: The auxin response factor 3 (103484487/XP_016899372.1), auxin response factor 1 (103499376/XP_008460595.1) and indole 3-acetic acid-induce protein ARG7 isoenzymes are more expressed in downstream 40 DAP fruit auxin pathway (-0.7381, -0.7397 and -1.13863 log2 FoldChange respectively). In the downstream ethylene and salicylic acid pathways the genes ethylene receptor 1 (103492012/XP_008450396.1/-1.285086703 log2FoldChange) and pathogenesis-related protein 1 (103495326/XP_008455060.1/-4.71658675 log2 FoldChange) are more expressed respectively. In the abscisic acid biosynthesis the 9-cis-epoxycarotenoid dioxygenase ((103493037XP_008451876.1/-1.79) is more expressed in 40 DAP fruit.

2) Carbon metabolism

ID	Gene Name	Species	Log2 FoldChange
103498931/XP_008459982.1	alcohol dehydrogenase class-3(LOC103498931)	Cucumis melo	0.7665
103485188/XP_008440909.1	aminomethyltransferase, mitochondrial(LOC103485188)	Cucumis melo	1.0930
103502185/XP_008464259.1	aspartate aminotransferase, mitochondrial-like(LOC103502185)	Cucumis melo	1.5208
103502619/XP_008464839.1	catalase isozyme 3(LOC103502619)	Cucumis melo	0.7841
103488872/XP_008446019.1	cysteine synthase(LOC103488872)	Cucumis melo	1.0895
103485981/XP_008441982.1	glutamate dehydrogenase 1(LOC103485981)	Cucumis melo	2.2010
103492923/XP_008451713.1	malate dehydrogenase, mitochondrial(LOC103492923)	Cucumis melo	1.0899
103489191/XP_008446456.1	probable 6-phosphogluconolactonase 1(LOC103489191)	Cucumis melo	1.1631
103483866/XP_016898966.1	probable acyl-CoA dehydrogenase IBR3(LOC103483866)	Cucumis melo	1.0748
103498321/XP_008459117.1	probable ribose-5-phosphate isomerase 4, chloroplastic(LOC103498321)	Cucumis melo	1.5180
103500862/XP_008462524.1	pyruvate kinase 2, cytosolic-like(LOC103500862)	Cucumis melo	1.5922
103483770/XP_008438755.1	pyruvate, phosphate dikinase, chloroplastic(LOC103483770)	Cucumis melo	1.6250
103503511/XP_008465941.1	ribose-phosphate pyrophosphokinase 1-like(LOC103503511)	Cucumis melo	1.2528
103489286/XP_008446602.1	ribulose bisphosphate carboxylase small chain, chloroplastic(LOC103489286)	Cucumis melo	1.5461
103485491/XP_008441351.1	ribulose-phosphate 3-epimerase, cytoplasmic isoform(LOC103485491)	Cucumis melo	1.0046
103484921/XP_016899268.1	uncharacterized LOC103484921(LOC103484921)	Cucumis melo	2.3091

3) Starch and sucrose metabolism

ID	Gene Name	Species	Log2 FoldChange
103504616/NP_001284469.1	acid beta-fructofuranosidase-like(INV2)	Cucumis melo	2.3437
103490714/MELO3C013786.2	alpha-glucosidase 2(LOC103490714)	Cucumis melo	1.0610
103494776/XP_008454350.1	beta-glucosidase 11-like(LOC103494776)	Cucumis melo	2.0601
103493887/XP_008453064.2	beta-glucosidase 18-like(LOC103493887)	Cucumis melo	1.7016
103492048/XP_008450452.1	beta-glucosidase 24(LOC103492048)	Cucumis melo	4.5275
103504550/XP_008467118.1	phosphoglucomutase, cytoplasmic(LOC103504550)	Cucumis melo	0.8392

103489022/ XP_008446229.1	probable alpha,alpha-trehalose-phosphate synthase [UDP-forming] 9(LOC103489022)	Cucumis melo	1.1209
103493790/XP_008452915.1	probable alpha-amylase 2(LOC103493790)	Cucumis melo	0.9488
103496894/XP_008457154.1	probable sucrose-phosphate synthase 2(LOC103496894)	Cucumis melo	1.6219
103484159/XP_008439346.1	probable trehalose-phosphate phosphatase C(LOC103484159)	Cucumis melo	3.7901
103501373/XP_008463167.1	sucrose synthase 2(LOC103501373)	Cucumis melo	3.8279

Obs.: The sucrose synthase 1(103492399/MELO3C004346, log2 FoldChange: -2.280855057), glucan endo -1,3 β – glucosidase (103485208/ XP_008440956.1/- log2 FoldChange: 1.8416), sucrose-phosphatase 1 (103486711/XP_008442968.1, log2 FoldChange: 0.829194958); Putative Trehalose-6-phosphate synthase (103490765/ XP_008448661.1, log2 FoldChange:-1.0656); Trehalose-6-phosphate synthase (103495491/XP_016901732.1, log2 FoldChange: -0.66752696); Beta-amylase (103493026/XP_008451866.1, log2 FoldChange:-1.246301429); Inactive β-amylase 4 (107990883/ XP_016900389.1, log2 FoldChange: 1.730691751) are more expressed in 40 DAP fruit.

4) Photosynthesis genes

ID	Gene Name	Species	Log2 FoldChange
103490694/XP_008448551.1	ferredoxin--NADP reductase, leaf isozyme, chloroplastic-like(LOC103490694)	Cucumis melo	1.1526
103484723/XP_008440181.1	oxygen-evolving enhancer protein 1, chloroplastic(LOC103484723)	Cucumis melo	0.6675
103494958/XP_008454569.1	photosynthetic NDH subunit of lumenal location 3, chloroplastic(LOC103494958)	Cucumis melo	1.8491
103483718/XP_008438687.1	photosystem I reaction center subunit II, chloroplastic(LOC103483718)	Cucumis melo	1.2652
103485855/XP_008441802.1	photosystem I reaction center subunit IV, chloroplastic-like(LOC103485855)	Cucumis melo	1.7186
103504056/XP_008466710.1	photosystem II 22 kDa protein, chloroplastic(LOC103504056)	Cucumis melo	4.5741
103501654/XP_008463515.1	psbP-like protein 1, chloroplastic(LOC103501654)	Cucumis melo	0.7721

5) Galactose metabolism

ID	Gene Name	Species	Log2 FoldChange
103504616/NP_001284469.1	acid beta-fructofuranosidase-like(INV2)	Cucumis melo	2.3437
103488794/XP_008445911.1	alpha-galactosidase(LOC103488794)	Cucumis melo	1.5210
103490714/MELO3C013786	alpha-glucosidase 2(LOC103490714)	Cucumis melo	1.0610
103504550/XP_008467118.1	phosphoglucomutase, cytoplasmic(LOC103504550)	Cucumis melo	0.8392
103499514/XP_008460758.1	probable galactinol--sucrose galactosyltransferase 2(LOC103499514)	Cucumis melo	1.0713
103487113/XP_008443553.1	probable galactinol--sucrose galactosyltransferase 5(LOC103487113)	Cucumis melo	2.4511
103492745/XP_008451468.1	stachyose synthase(LOC103492745)	Cucumis melo	2.4118

The raffinose synthase (XP_008443958.1/ 103487423, log2 FoldChange: -0.933814416), ATP-dependent 6-phosphofructokinase (XP_008440953.1 / 103485214, Log2FoldChange: -1.185646773) and alpha-glucosidase (MELO3C004346.2, log2FoldChange: -2.2808) are more expressed in 40 DAP fruit.

6) Carbon fixation in photosynthetic organisms

ID	Gene Name	Species	Log2 FoldChange
103502185/XP_008464259.1	aspartate aminotransferase, mitochondrial-like(LOC103502185)	Cucumis melo	1.5208
103492923/XP_008451713.1	malate dehydrogenase, mitochondrial(LOC103492923)	Cucumis melo	1.0899
103498321/XP_008459117.1	probable ribose-5-phosphate isomerase 4, chloroplastic(LOC103498321)	Cucumis melo	1.5180
103483770/XP_008438755.1	pyruvate, phosphate dikinase, chloroplastic(LOC103483770)	Cucumis melo	1.6250
103489286/XP_008446602.1	ribulose bisphosphate carboxylase small chain, chloroplastic(LOC103489286)	Cucumis melo	1.5461
103485491/XP_008441351.1	ribulose-phosphate 3-epimerase, cytoplasmic isoform(LOC103485491)	Cucumis melo	1.0046

40 DAP

1) Protein processing in endoplasmic reticulum

ID	Gene Name	Species	Log2 FoldChange
103501364/XP_008463154.1	15.7 kDa heat shock protein, peroxisomal(LOC103501364)	Cucumis melo	-1.8992
103502478/XP_008464642.1	17.8 kDa class I heat shock protein-like(LOC103502478)	Cucumis melo	-1.0400
103490772/XP_008448675.1	18.1 kDa class I heat shock protein-like(LOC103490772)	Cucumis melo	-2.9855
103497111/XP_008457419.1	18.5 kDa class I heat shock protein-like(LOC103497111)	Cucumis melo	-3.7264
103497112/XP_008457420.1	18.5 kDa class I heat shock protein-like(LOC103497112)	Cucumis melo	-1.4691
103495518/XP_008455327.1	E3 ubiquitin-protein ligase RMA1H1(LOC103495518)	Cucumis melo	-1.2754
103500816/XP_008462466.1	cell division cycle protein 48 homolog(LOC103500816)	Cucumis melo	-0.7106
103502393/XP_008464542.1	cell division cycle protein 48 homolog(LOC103502393)	Cucumis melo	-0.7344
103491749/XP_008450042.1	class I heat shock protein-like(LOC103491749)	Cucumis melo	-3.4016
103492771/XP_008451505.1	dnaJ protein ERDJ3B(LOC103492771)	Cucumis melo	-0.7516
103489367/XP_008446732.1	dnaJ protein homolog(LOC103489367)	Cucumis melo	-3.5188
103488055/XP_008444821.1	endoplasmin homolog(LOC103488055)	Cucumis melo	-0.7410
103492106/XP_008450536.1	heat shock 70 kDa protein(LOC103492106)	Cucumis melo	-3.0452
103498164/XP_008458892.1	heat shock cognate 70 kDa protein(LOC103498164)	Cucumis melo	-0.8653
103484110/XP_008439282.1	heat shock protein 83(LOC103484110)	Cucumis melo	-0.9152
103504539/XP_008467106.1	plant UBX domain-containing protein 2(LOC103504539)	Cucumis melo	-2.1817
103495249/XP_008454966.1	probable E3 ubiquitin ligase SUD1(LOC103495249)	Cucumis melo	-0.6980
103487399/XP_008443923.1	protein OS-9 homolog(LOC103487399)	Cucumis melo	-2.2872
103483120/XP_008437786.1	protein transport protein Sec61 subunit beta(LOC103483120)	Cucumis melo	-1.2158
103483006/ MELO3C013946.2	small heat shock protein, chloroplastic(LOC103483006)	Cucumis melo	-1.4319

103488933/XP_008446109.1	small heat shock protein, chloroplastic-like(LOC103488933)	Cucumis melo	-1.8242
103501214/XP_008462954.1	ubiquitin receptor RAD23b(LOC103501214)	Cucumis melo	-0.6879
103501476/XP_008463276.1	ubiquitin-conjugating enzyme E2 28(LOC103501476)	Cucumis melo	-0.7018
103483220/XP_008437957.1	ubiquitin-conjugating enzyme E2-17 kDa-like(LOC103483220)	Cucumis melo	-0.7910

2) Spliceosome

ID	Gene Name	Species	Log2 FoldChange
103494190/XP_008453490.1	DEAD-box ATP-dependent RNA helicase 42(LOC103494190)	Cucumis melo	-0.9660
103484204/XP_008439389.1	DExH-box ATP-dependent RNA helicase DExH12-like(LOC103484204)	Cucumis melo	-2.0368
103499294/XP_008460496.1	THO complex subunit 1(LOC103499294)	Cucumis melo	-0.9537
103497214/XP_008457549.1	THO complex subunit 4A(LOC103497214)	Cucumis melo	-0.7584
103502534/XP_008464716.1	U4/U6 small nuclear ribonucleoprotein PRP4-like protein(LOC103502534)	Cucumis melo	-1.0175
103494318/XP_008453669.1	cell division cycle 5-like protein(LOC103494318)	Cucumis melo	-1.1004
103500640/XP_008462238.1	crooked neck-like protein 1(LOC103500640)	Cucumis melo	-0.8044
103494047/XP_008453280.1	glycine-rich RNA-binding protein 2, mitochondrial-like(LOC103494047)	Cucumis melo	-1.1219
103492106/XP_008450536.1	heat shock 70 kDa protein(LOC103492106)	Cucumis melo	-3.0452
103498164/XP_008458892.1	heat shock cognate 70 kDa protein(LOC103498164)	Cucumis melo	-0.8653
103483553/XP_008438467.1	pre-mRNA-processing factor 19(LOC103483553)	Cucumis melo	-0.7412
103495367/XP_008455119.1	pre-mRNA-splicing factor SLU7(LOC103495367)	Cucumis melo	-0.6952
103483592/XP_008438525.1	pre-mRNA-splicing factor SLU7-like(LOC103483592)	Cucumis melo	-0.8591
103494929/XP_008454536.1	probable U6 snRNA-associated Sm-like protein LSm4(LOC103494929)	Cucumis melo	-1.5835
103497843/XP_008458420.1	protein pleiotropic regulatory locus 1(LOC103497843)	Cucumis melo	-2.3955
103485621/XP_008441520.1	sm-like protein LSM3B(LOC103485621)	Cucumis melo	-1.0844
103501154/XP_008462876.1	splicing factor 3B subunit 1(LOC103501154)	Cucumis melo	-0.7527

3) Carbon metabolism

ID	Gene Name	Species	Log2 FoldChange
103493120/XP_008451978.1	6-phosphogluconate dehydrogenase, decarboxylating 2, chloroplastic(LOC103493120)	Cucumis melo	-1.0763
103485214/XP_008440953.1	ATP-dependent 6-phosphofructokinase 2(LOC103485214)	Cucumis melo	-1.1856
103488170/XP_008445000.2	NADP-dependent malic enzyme(LOC103488170)	Cucumis melo	-0.8914
103489969/XP_008447551.1	NADP-dependent malic enzyme(LOC103489969)	Cucumis melo	-0.9623
103492256/XP_008450776.1	NADP-dependent malic enzyme(LOC103492256)	Cucumis melo	-0.7131
103502447/XP_008464603.1	S-formylglutathione hydrolase(LOC103502447)	Cucumis melo	-0.7331
103500505/XP_008462041.1	acetyl-CoA acetyltransferase, cytosolic 1(LOC103500505)	Cucumis melo	-1.2583
103485166/XP_008440877.1	aconitate hydratase(LOC103485166)	Cucumis melo	-0.6218
103492249/XP_008450767.2	cysteine synthase-like(LOC103492249)	Cucumis melo	-1.2556
103492251/XP_008450770.1	cysteine synthase-like(LOC103492251)	Cucumis melo	-2.3226
103487718/XP_008444367.1	glutamate--glyoxylate aminotransferase 2(LOC103487718)	Cucumis melo	-2.4882
103484230/XP_008439417.1	glyceraldehyde-3-phosphate dehydrogenase 2, cytosolic(LOC103484230)	Cucumis melo	-0.9103
103493832/XP_008452998.1	glyceraldehyde-3-phosphate dehydrogenase B, chloroplastic(LOC103493832)	Cucumis melo	-1.7013
103503632/XP_008466111.1	phosphoenolpyruvate carboxykinase [ATP](LOC103503632)	Cucumis melo	-1.1531
103491219/XP_008449299.1	plastidial pyruvate kinase 1, chloroplastic(LOC103491219)	Cucumis melo	-0.7581
103482989/XP_008437645.1	probable 6-phosphogluconolactonase 4, chloroplastic(LOC103482989)	Cucumis melo	-0.8031

4) Ribosome biogenesis in eukaryotes

ID	Gene Name	Species	Log2 FoldChange
103495169/XP_008454886.1	H/ACA ribonucleoprotein complex subunit 2-like protein(LOC103495169)	Cucumis melo	-0.8041
103491111/XP_008449192.1	H/ACA ribonucleoprotein complex subunit 4(LOC103491111)	Cucumis melo	-1.9299
103497366/XP_008457746.1	U3 small nucleolar RNA-associated protein 18 homolog(LOC103497366)	Cucumis melo	-0.9785
103500839/XP_008462502.1	WD repeat-containing protein 3(LOC103500839)	Cucumis melo	-1.0910
103503484/XP_008465906.1	casein kinase II subunit beta-like(LOC103503484)	Cucumis melo	-1.0937
103484122/XP_008439295.1	mediator of RNA polymerase II transcription subunit 36a-like(LOC103484122)	Cucumis melo	-1.1570
103502340/XP_008464470.1	nucleolar protein 56-like(LOC103502340)	Cucumis melo	-1.1028
103498805/XP_008459771.1	nucleolar protein 6(LOC103498805)	Cucumis melo	-2.3495
103495781/XP_008455669.1	probable nucleolar protein 5-2(LOC103495781)	Cucumis melo	-1.4378
103490911/XP_008448880.1	ribosomal RNA small subunit methyltransferase NEP1-like(LOC103490911)	Cucumis melo	-1.9151
103494871/XP_008454475.1	uncharacterized protein At3g06530(LOC103494871)	Cucumis melo	-2.9112

5) Carbon fixation in photosynthetic organisms

ID	Gene Name	Species	Log2 FoldChange
103488170/XP_008445000.2	NADP-dependent malic enzyme(LOC103488170)	Cucumis melo	-0.8914
103489969/XP_008447551.1	NADP-dependent malic enzyme(LOC103489969)	Cucumis melo	-0.9623
103492256/XP_008444367.1	NADP-dependent malic enzyme(LOC103492256)	Cucumis melo	-2.4882
103487718/XP_008444367.1	glutamate--glyoxylate aminotransferase 2(LOC103487718)	Cucumis melo	-2.4882
103484230/XP_008439417.1	glyceraldehyde-3-phosphate dehydrogenase 2, cytosolic(LOC103484230)	Cucumis melo	-0.9103
103493832/ XP_008452998.1	glyceraldehyde-3-phosphate dehydrogenase B, chloroplastic(LOC103493832)	Cucumis melo	-1.7013
103503632/ XP_008466111.1	phosphoenolpyruvate carboxykinase [ATP](LOC103503632)	Cucumis melo	-1.1531

6) **Pyruvate metabolism**

ID	Gene Name	Species	Log2 FoldChange
103488170/XP_008445000.2	NADP-dependent malic enzyme(LOC103488170)	Cucumis melo	-0.8914
103489969/XP_008447551.1	NADP-dependent malic enzyme(LOC103489969)	Cucumis melo	-0.9623
103492256/XP_008450776.1	NADP-dependent malic enzyme(LOC103492256)	Cucumis melo	-0.7131
103500505/XP_008462041.1	acetyl-CoA acetyltransferase, cytosolic 1(LOC103500505)	Cucumis melo	-1.2583
103503632/XP_008466111.1	phosphoenolpyruvate carboxykinase [ATP](LOC103503632)	Cucumis melo	-1.1531
103491219/XP_008449299.1	plastidial pyruvate kinase 1, chloroplastic(LOC103491219)	Cucumis melo	-0.7581
103494619/XP_008454116.1	probable hydroxyacylglutathione hydrolase 2, chloroplastic(LOC103494619)	Cucumis melo	-0.9479
