# Supplementary material for: Transcriptome profiling of non-climacteric ‘yellow’ melon during ripening: insights on sugar metabolism
Source: BMC Genomics. 2020 Mar 30;21:262. doi: 10.1186/s12864-020-6667-0 (PMC7106763; doi:10.1186/s12864-020-6667-0)
Supplement: Supplementary file 8 — Additional file 8: Figures S5, S6 and Tables S7, S8. Figures represent protein–protein interaction network of young (Figure S4) and mature melon (Figure S5) fruit generated by STRING and Cytoscape analyses. The tables represent the characteristics of the network interaction. [file 12864_2020_6667_MOESM8_ESM.pdf]

10 DAP fruit

Figure S5 10 DAP fruit network protein interaction (STRING software). The color are represented by functional enrichment in the network (legend). Nodes represent related proteins. Edges represent protein–protein associations.

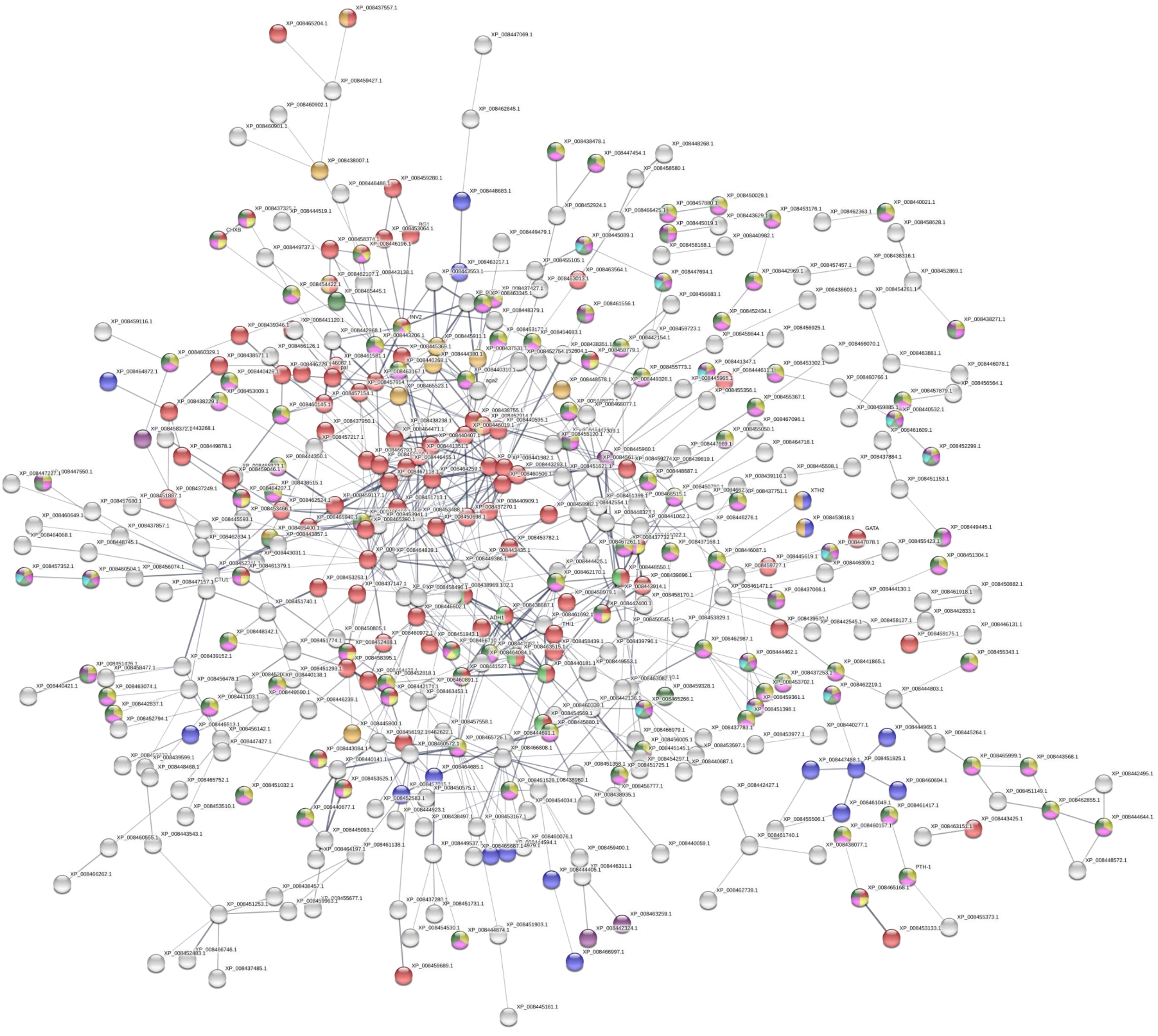

Legend:

KEGG Pathways			
pathway	*description*	*count in gene set*	*false discovery rate*
cmo01100	Metabolic pathways	96 of 1685	0.0035
cmo04075	Plant hormone signal transduction	23 of 256	0.0095
cmo00195	Photosynthesis	7 of 40	0.0427

UniProt Keywords			
keyword	*description*	*count in gene set*	*false discovery rate*
KW-1133	Transmembrane helix	193 of 3925	0.0018
KW-0812	Transmembrane	196 of 3941	0.0018
KW-0472	Membrane	199 of 4043	0.0018
KW-0406	Ion transport	16 of 134	0.0042
KW-0326	Glycosidase	21 of 224	0.0072
KW-0813	Transport	37 of 538	0.0132

Obs.: 1 gene of “Metabolic pathways”, 6 genes of “Plant hormone signal transduction”, 88 genes of “Transmembrane helix”, 91 of “Transmembrane”, 91 genes of “membrane”, 3 genes of “Ion transport”, 6 genes of “Glycosidases”, 12 genes of “Transport” are disconnected nodes in the network.

Table S7 10 DAP network profile (Cytoscape software)

Name/Refeseq ID	BetweennessCentrality	ClosenessCentrality	Degree	NumberOfDirectedEdges
**XP_008453488.1**	0.11244542	0.28940986	26	26
**XP_008441351.1**	0.12321749	0.28778135	20	20
**XP_008464259.1**	0.09142517	0.27816628	19	19
**XP_008438755.1**	0.06632325	0.26150475	15	15
**XP_008464839.1**	0.15539623	0.28034456	15	15
**XP_008467118.1**	0.03528732	0.27286585	15	15
**XP_008451713.1**	0.02773533	0.27559661	13	13
**XP_008440181.1**	0.0235859	0.23614776	13	13
**XP_008452241.1**	0.14318946	0.24706694	13	13
**XP_008441982.1**	0.04207467	0.25300353	13	13
**XP_008465726.1**	0.11418729	0.25923244	13	13
**XP_008456121.1**	0.07605292	0.24320652	13	13
**XP_008438687.1**	0.05377935	0.26420664	12	12
**XP_008458506.1**	0.05832615	0.27265804	11	11
**XP_008458496.1**	0.04872582	0.26169591	11	11
**XP_008460506.1**	0.01264813	0.25264644	11	11
**XP_008466808.1**	0.03713439	0.20492272	11	11
**XP_008441527.1**	0.04757811	0.2443686	11	11
**XP_008462170.1**	0.01740133	0.24420191	11	11
**XP_008446602.1**	0.03979069	0.25998548	10	10
**XP_008443206.1**	0.06427999	0.2405914	10	10
**XP_008452914.1**	0.04173978	0.24706694	10	10
**XP_008450545.1**	0.06334894	0.21385902	10	10
**XP_008464084.1**	0.02052228	0.25175809	10	10
**XP_008440909.1**	0.01853812	0.26459719	9	9
**XP_008443435.1**	0.06345302	0.27453988	9	9
**XP_008463440.1**	0.05573923	0.25211268	9	9
**XP_008467212.1**	0.02122836	0.20044793	9	9
**XP_008448550.1**	0.10639878	0.24537354	9	9
**XP_008460572.1**	0.04589427	0.22759059	9	9
**XP_008459117.1**	0.01340061	0.25663082	9	9
**XP_008463167.1**	0.01510519	0.21869273	9	9
**XP_008465390.1**	0.02989366	0.24189189	8	8
**XP_008444380.1**	0.0125398	0.22153465	8	8
**XP_008453782.1**	0.0239353	0.24320652	8	8
**XP_008446455.1**	0.00978878	0.25626342	8	8
**XP_008454560.1**	0.0500793	0.22222222	8	8
**XP_008465940.1**	0.06163388	0.25773938	8	8
**XP_008452488.1**	0.05691852	0.2306701	8	8
**XP_008437270.1**	0.01778418	0.24587912	8	8
**XP_008440595.1**	0.01089787	0.24337186	8	8
**XP_008452100.1**	0.03204604	0.2573688	8	8
**XP_008446019.1**	0.01825703	0.23413996	8	8
**XP_008445911.1**	0.01368921	0.21398685	8	8
**XP_008463515.1**	0.01101925	0.24843858	7	7
**XP_008438960.1**	0.06230861	0.22445141	7	7
**XP_008445960.1**	0.01479382	0.22572509	7	7

**XP_008448323.1**	0.02545825	0.22904671	7	7
**XP_008461692.1**	0.00864161	0.24403545	7	7
**XP_008444691.1**	0.02685242	0.22759059	7	7
**XP_008457154.1**	0.02901766	0.22759059	7	7
**XP_008460758.1**	0.01599232	0.20123665	7	7
**XP_008438969.1**	0.02391207	0.24403545	7	7
**XP_008442554.1**	0.06941288	0.23429319	7	7
**XP_008452754.1**	0.05619697	0.24554184	6	6
**XP_008451253.1**	0.01872709	0.14111155	6	6
**XP_008442136.1**	0.0256267	0.2201722	6	6
**XP_008450780.1**	0.02455748	0.2039886	6	6
**XP_008441062.1**	0.02657056	0.20492272	6	6
**XP_008440141.1**	0.03897075	0.22222222	6	6
**XP_008464471.1**	0.00675308	0.23007712	6	6
**XP_008449386.1**	0.01294897	0.25626342	6	6
**XP_008443293.1**	0.01710749	0.24965132	6	6
**XP_008437297.1**	0.00757642	0.23583663	6	6
**XP_008465523.1**	0.01510251	0.22194668	6	6
**XP_008444462.1**	0.00821639	0.18340164	6	6
**XP_008451774.1**	0.03132797	0.22459222	6	6
**XP_008442171.1**	0.02244632	0.2201722	6	6
**XP_008464685.1**	0.0370183	0.2144997	6	6
**XP_008466022.1**	0.01976409	0.21021726	6	6
**XP_008440407.1**	0.00738425	0.23126615	6	6
**XP_008440310.1**	0.02380382	0.23537147	6	6
**XP_008448578.1**	0.02059592	0.22658228	5	5
**XP_008466793.1**	0.00683113	0.25300353	5	5
**XP_008459274.1**	0.01169887	0.2169697	5	5
**XP_008460972.1**	0.0183405	0.22153465	5	5
**XP_008451925.1**	0.83333333	0.85714286	5	5
**XP_008456478.1**	0.03758571	0.20681687	5	5
**XP_008459727.1**	0.02066464	0.19648738	5	5
**XP_008466077.1**	0.0168034	0.23946488	5	5
**XP_008451468.1**	1.12E+00	0.18921776	5	5
**XP_008442400.1**	0.00547248	0.19757174	5	5
**XP_008462855.1**	0.01668153	0.09293873	5	5
**XP_008462987.1**	0.01438425	0.19288793	5	5
**XP_008462524.1**	0.03410722	0.25034965	5	5
**XP_008453941.1**	0.00100177	0.24965132	5	5
**XP_008456142.1**	0.0311242	0.1967033	5	5
**XP_008450698.1**	0.00382152	0.23708609	5	5
**XP_008461471.1**	0.02471887	0.19833795	5	5
**XP_008466102.1**	0.01755255	0.20515759	4	4
**XP_008457914.1**	0.01779578	0.22071517	4	4
**XP_008445880.1**	0.01113387	0.22126082	4	4
**XP_008456777.1**	0.00558659	0.18368394	4	4
**XP_008445369.1**	0.01766872	0.23661599	4	4
**XP_008441802.1**	0.00246951	0.24554184	4	4
**XP_008456192.1**	0.01410017	0.23111685	4	4

**XP_008443062.1**	0.00830748	0.22403004	4	4
**XP_008439152.1**	0.02297644	0.20504009	4	4
**XP_008443914.1**	0.00290622	0.21909425	4	4
**XP_008438007.1**	0.02772953	0.16429555	4	4
**XP_008451740.1**	0.00463293	0.1967033	4	4
**XP_008443553.1**	1.79E-02	0.18558839	4	4
**XP_008449537.1**	0.01671283	0.17722772	4	4
**XP_008458395.1**	0.00893971	0.22788033	4	4
**XP_008460145.1**	0.00409463	0.19498911	4	4
**XP_008438229.1**	0.00686508	0.17211538	4	4
**XP_008446087.1**	0.01862769	0.2037564	4	4
**XP_008443031.1**	0.01772099	0.21083628	4	4
**XP_008457015.1**	0.03580261	0.21233689	4	4
**XP_008446229.1**	0.00558659	0.19299191	4	4
**XP_008453253.1**	0.00779374	0.21644498	4	4
**XP_008445093.1**	0.00558659	0.1821883	3	3
**XP_008455120.1**	0.00784645	0.22319202	3	3
**XP_008461740.1**	1	1	3	3
**XP_008439896.1**	0	0.17687747	3	3
**XP_008461491.1**	0.00669022	0.23307292	3	3
**XP_008442968.1**	0	0.19467102	3	3
**XP_008437857.1**	0.01115754	0.19866815	3	3
**XP_008459427.1**	0.01115754	0.14139021	3	3
**XP_008442154.1**	0.00242505	0.18321392	3	3
**XP_008461138.1**	0.02329975	0.17687747	3	3
**XP_008461379.1**	0.00851987	0.19012215	3	3
**XP_008446311.1**	0.01115754	0.18377823	3	3
**XP_008444803.1**	0.04927781	0.14517437	3	3
**XP_008456005.1**	0	0.18358974	3	3
**XP_008458170.1**	0	0.17687747	3	3
**XP_008460329.1**	0.01061239	0.16886792	3	3
**XP_008441103.1**	0.01115754	0.18387262	3	3
**XP_008452604.1**	0	0.20911215	3	3
**XP_008455105.1**	0.00653831	0.20899008	3	3
**XP_008453167.1**	0.01114189	0.17687747	3	3
**XP_008438238.1**	0.00558659	0.17935872	3	3
**XP_008443138.1**	0.01115754	0.19185423	3	3
**XP_008465999.1**	0.03305792	0.11350666	3	3
**XP_008466425.1**	0.01114189	0.16558742	3	3
**XP_008463217.1**	0.01790507	0.22529893	3	3
**XP_008451358.1**	0.00825932	0.21579265	3	3
**XP_008460891.1**	0.00863946	0.21385902	3	3
**XP_008451293.1**	0.01097107	0.20011179	3	3
**XP_008457558.1**	0.01023284	0.19637959	3	3
**XP_008455773.1**	0.00608447	0.2085032	3	3
**XP_008462622.1**	0.00663791	0.20123665	3	3
**XP_008452924.1**	0.01115754	0.16558742	3	3
**XP_008451621.1**	0.00181765	0.21083628	3	3
**XP_008463881.1**	1	1	3	3

**XP_008464423.1**	0.00332518	0.20457143	3	3
**XP_008459982.1**	0.00488477	0.24982554	3	3
**XP_008439530.1**	0.07052877	0.2	3	3
**XP_008445593.1**	0.00851811	0.19691969	3	3
**XP_008454422.1**	0.01114189	0.18126582	3	3
**XP_008458439.1**	0.02464458	0.23568137	3	3
**XP_008449326.1**	0.01799764	0.17740337	3	3
**XP_008454458.1**	0.00784645	0.22319202	3	3
**XP_008458374.1**	5.48E-02	0.19488296	3	3
**XP_008466710.1**	7.94E+00	0.22759059	3	3
**XP_008461581.1**	0.01246501	0.23307292	3	3
**XP_008444644.1**	7.82E-06	0.08509627	3	3
**XP_008446196.1**	0.03305009	0.19541485	3	3
**XP_008467309.1**	0.00367952	0.18209563	2	2
**XP_008465687.1**	0.00261573	0.2146283	2	2
**XP_008451528.1**	0.00115098	0.19340897	2	2
**XP_008448687.1**	0.00558659	0.19012215	2	2
**XP_008459963.1**	0.00676904	0.15331906	2	2
**XP_008448872.1**	1.44E+00	0.21046443	2	2
**XP_008458979.1**	8.45E+00	0.20789779	2	2
**XP_008447669.1**	0.01114189	0.1903243	2	2
**XP_008445619.1**	0.00558659	0.19735391	2	2
**XP_008459361.1**	7.90E+00	0.17429406	2	2
**XP_008454979.1**	0.00261573	0.2146283	2	2
**XP_008460504.1**	0.00558659	0.19844789	2	2
**XP_008444425.1**	0.0019317	0.20960187	2	2
**XP_008464207.1**	0.00648971	0.20610248	2	2
**XP_008460766.1**	0.66666667	0.75	2	2
**XP_008463013.1**	0.01666588	0.19789939	2	2
**XP_008459844.1**	0.00558659	0.15086389	2	2
**XP_008450452.1**	0.00278547	0.16721158	2	2
**XP_008455506.1**	0.03333333	0.46153846	2	2
**XP_008459723.1**	0.00348815	0.19467102	2	2
**XP_008459328.1**	0.00183668	0.18626431	2	2
**XP_008463969.1**	0.00558659	0.2180268	2	2
**XP_008438571.1**	0.00738796	0.19083156	2	2
**XP_008465752.1**	0.01114189	0.16474919	2	2
**XP_008440677.1**	0	0.18209563	2	2
**XP_008466979.1**	0.00103928	0.18340164	2	2
**XP_008446276.1**	0	0.170233	2	2
**XP_008442969.1**	1	1	2	2
**XP_008438497.1**	6.18E+00	0.1919571	2	2
**XP_008451398.1**	7.90E+00	0.17429406	2	2
**XP_008437253.1**	0	0.1773155	2	2
**XP_008453064.1**	0.00278547	0.16721158	2	2
**XP_008465445.1**	0.00187508	0.15150233	2	2
**XP_008446309.1**	0.00558659	0.16574074	2	2
**XP_008440532.1**	0.66666667	0.75	2	2
**XP_008466520.1**	0	0.22388993	2	2

**XP_008444130.1**	0.00558659	0.16689977	2	2
**XP_008444405.1**	0.00558659	0.18358974	2	2
**XP_008443568.1**	0.01104799	0.10219812	2	2
**XP_008448379.1**	0.00242023	0.15417743	2	2
**XP_008457217.1**	0.00379475	0.19988833	2	2
**XP_008448683.1**	0.01114189	0.18434604	2	2
**XP_008462845.1**	0.00558659	0.15585546	2	2
**XP_008457680.1**	0.00558659	0.19844789	2	2
**XP_008460339.1**	0.00173466	0.19616438	2	2
**XP_008448268.1**	0	0.14217633	2	2
**XP_008449878.1**	0.00140084	0.17269658	2	2
**XP_008443543.1**	0.01060086	0.15105485	2	2
**XP_008442495.1**	0	0.08507605	2	2
**XP_008451903.1**	0.00558659	0.1504834	2	2
**XP_008452869.1**	1	1	2	2
**XP_008456683.1**	3.53E+00	0.16918715	2	2
**XP_008459046.1**	0.00558659	0.20056022	2	2
**XP_008437732.1**	0	0.19757174	2	2
**XP_008451149.1**	0.01104799	0.10219812	2	2
**XP_008454693.1**	8.05E+00	0.205393	2	2
**XP_008444594.1**	0.00261573	0.2146283	2	2
**XP_008460076.1**	0.00261573	0.2146283	2	2
**XP_008439286.1**	0	0.15358215	2	2
**XP_008447488.1**	0.13333333	0.6	2	2
**XP_008465266.1**	0.00738734	0.19746277	2	2
**XP_008447694.1**	7.62E+00	0.1752325	2	2
**XP_008438457.1**	0.00676904	0.15331906	2	2
**XP_008465168.1**	1	1	2	2
**XP_008445513.1**	0.00558659	0.15840708	2	2
**XP_008441865.1**	0.05445754	0.16831218	2	2
**XP_008437066.1**	0.00568282	0.18008048	2	2
**XP_008446239.1**	0.00558659	0.20669746	2	2
**XP_008461049.1**	0.13333333	0.6	2	2
**XP_008448572.1**	0	0.08507605	2	2
**XP_008445264.1**	0.0384489	0.12744749	2	2
**XP_008448342.1**	0	0.18665276	2	2
**XP_008449590.1**	0.01114189	0.18792651	2	2
**XP_008460555.1**	0.00558659	0.14161392	2	2
**XP_008445303.1**	4.30E+00	0.17228104	2	2
**XP_008437325.1**	0	0.15358215	2	2
**XP_008463564.1**	0.01666588	0.19789939	2	2
**XP_008437280.1**	0.00558659	0.18568465	2	2
**XP_008453525.1**	0.00558659	0.18071681	2	2
**XP_008445089.1**	8.34E+00	0.1761811	2	2
**XP_008452818.1**	0	0.21670702	2	2
**XP_008458779.1**	0.00437573	0.22948718	2	2
**XP_008445800.1**	0.00107708	0.19988833	2	2
**XP_008458372.1**	0.01511504	0.1994429	2	2
**XP_008439116.1**	0	0.170233	2	2

**XP_008437249.1**	0.0035429	0.17600787	2	2
**XP_008437531.1**	0.00558659	0.19002123	2	2
**XP_008462107.1**	0	0.19414317	2	2
**XP_008463453.1**	0.00212988	0.20527523	2	2
**XP_008439599.1**	0.01516219	0.17286335	2	2
**XP_008444611.1**	0.00558659	0.19594964	2	2
**XP_008465923.1**	0.00128301	0.17303045	2	2
**XP_008451426.1**	0.00558659	0.17039505	2	2
**XP_008455367.1**	0.00558659	0.16010733	2	2
**XP_008437168.1**	0.00214761	0.18931782	2	2
**XP_008453702.1**	7.90E+00	0.17429406	2	2
**XP_008448745.1**	0.00558659	0.19844789	2	2
**XP_008466515.1**	3.26E+00	0.19175147	2	2
**XP_008451153.1**	1	1	2	2
**XP_008459280.1**	7.82E-06	0.14342949	2	2
**XP_008443857.1**	0.00163421	0.22963438	2	2
**XP_008450575.1**	0	0.19320022	2	2
**XP_008458580.1**	0	0.14217633	2	2
**XP_008465055.1**	1	1	2	2
**XP_008437147.1**	0.00948805	0.22701332	2	2
**XP_008450805.1**	2.34E+00	0.19257665	2	2
**XP_008439819.1**	0.01014352	0.20777713	2	2
**XP_008461217.1**	0	0.17908954	1	1
**XP_008445598.1**	0	1	1	1
**XP_008465204.1**	0	0.12391831	1	1
**XP_008461556.1**	0	0.2020316	1	1
**XP_008455373.1**	0	0.66666667	1	1
**XP_008447427.1**	0	0.16682199	1	1
**XP_008461918.1**	0	1	1	1
**XP_008451943.1**	0	0.19627193	1	1
**XP_008448468.1**	0	0.15538194	1	1
**XP_008453829.1**	0	0.19637959	1	1
**XP_008465400.1**	0	0.17031399	1	1
**XP_008438603.1**	0	0.13113553	1	1
**XP_008451725.1**	0	0.19113721	1	1
**XP_008464197.1**	0	0.15312233	1	1
**XP_008438351.1**	0	0.2020316	1	1
**XP_008456074.1**	0	0.19822813	1	1
**XP_008466126.1**	0	0.2144997	1	1
**XP_008441347.1**	0	0.19573537	1	1
**XP_008459175.1**	0	1	1	1
**XP_008457880.1**	0	1	1	1
**XP_008466070.1**	0	0.13806402	1	1
**XP_008447078.1**	0	0.16429555	1	1
**XP_008458628.1**	0	0.66666667	1	1
**XP_008453009.1**	0	0.22459222	1	1
**XP_008457457.1**	0	1	1	1
**XP_008453466.1**	0	0.21909425	1	1
**XP_008462739.1**	0	0.6	1	1

**XP_008453172.1**	0	0.18981972	1	1
**XP_008437485.1**	0	0.12370422	1	1
**XP_008450029.1**	0	1	1	1
**XP_008447069.1**	0	0.13489073	1	1
**XP_008458168.1**	0	1	1	1
**XP_008440687.1**	0	0.18191057	1	1
**XP_008453621.1**	0	0.16934721	1	1
**XP_008440021.1**	0	1	1	1
**XP_008440982.1**	0	1	1	1
**XP_008453597.1**	0	0.18191057	1	1
**XP_008442837.1**	0	0.17145594	1	1
**XP_008446131.1**	0	1	1	1
**XP_008449505.1**	0	0.16490097	1	1
**XP_008454530.1**	0	0.15061001	1	1
**XP_008454034.1**	0	0.18549223	1	1
**XP_008458127.1**	0	1	1	1
**XP_008461609.1**	0	0.66666667	1	1
**XP_008452483.1**	0	0.12370422	1	1
**XP_008452299.1**	0	0.5	1	1
**XP_008462834.1**	0	0.21437126	1	1
**XP_008463345.1**	0	0.20741599	1	1
**XP_008443425.1**	0	1	1	1
**XP_008467261.1**	0	0.19573537	1	1
**XP_008445161.1**	0	0.13084795	1	1
**XP_008462363.1**	0	1	1	1
**XP_008456925.1**	0	0.16391941	1	1
**XP_008456564.1**	0	1	1	1
**XP_008463082.1**	0	0.20146314	1	1
**XP_008463259.1**	0	0.15531453	1	1
**XP_008444350.1**	0	0.2043379	1	1
**XP_008455677.1**	0	0.15417743	1	1
**XP_008440428.1**	0	0.22459222	1	1
**XP_008464068.1**	0	0.16566404	1	1
**XP_008442427.1**	0	0.6	1	1
**XP_008458477.1**	0	0.170233	1	1
**XP_008460649.1**	0	0.16581751	1	1
**XP_008444923.1**	0	0.18549223	1	1
**XP_008443084.1**	0	0.18053454	1	1
**XP_008452372.1**	0	0.13679786	1	1
**XP_008457879.1**	0	1	1	1
**XP_008453510.1**	0	0.16444649	1	1
**XP_008445019.1**	0	1	1	1
**XP_008446486.1**	0	0.16104363	1	1
**XP_008449737.1**	0	0.19403794	1	1
**XP_008455050.1**	0	0.15982143	1	1
**XP_008438478.1**	0	0.14211989	1	1
**XP_008453302.1**	0	0.5	1	1
**XP_008442833.1**	0	1	1	1
**XP_008437427.1**	0	0.19062833	1	1

**XP_008440268.1**	0	0.18792651	1	1
**XP_008454569.1**	0	0.19911012	1	1
**XP_008444519.1**	0	0.16104363	1	1
**XP_008442545.1**	0	0.16558742	1	1
**XP_008441120.1**	0	0.1521462	1	1
**XP_008437557.1**	0	0.12391831	1	1
**XP_008453133.1**	0	0.66666667	1	1
**XP_008464718.1**	0	0.16950758	1	1
**XP_008437950.1**	0	0.2144997	1	1
**XP_008453977.1**	0	1	1	1
**XP_008460902.1**	0	0.14116719	1	1
**XP_008447550.1**	0	0.16581751	1	1
**XP_008466997.1**	0	0.15517989	1	1
**XP_008463074.1**	0	0.17145594	1	1
**XP_008452794.1**	0	0.15538194	1	1
**XP_008452583.1**	0	0.18549223	1	1
**XP_008444965.1**	0	0.5	1	1
**XP_008455356.1**	0	0.17837569	1	1
**XP_008437884.1**	0	1	1	1
**XP_008455423.1**	0	0.14223282	1	1
**XP_008460694.1**	0	0.5	1	1
**XP_008454297.1**	0	0.18053454	1	1
**XP_008460901.1**	0	0.14116719	1	1
**XP_008462219.1**	0	0.5	1	1
**XP_008440138.1**	0	0.18753274	1	1
**XP_008466228.1**	0	0.17015209	1	1
**XP_008445965.1**	0	0.18425116	1	1
**XP_008437751.1**	0	0.17378641	1	1
**XP_008451304.1**	0	0.14308553	1	1
**XP_008443268.1**	0	0.16713352	1	1
**XP_008446082.1**	0	0.18714062	1	1
**XP_008457352.1**	0	0.16566404	1	1
**XP_008467096.1**	0	0.16950758	1	1
**XP_008454261.1**	0	0.6	1	1
**XP_008463151.1**	0	1	1	1
**XP_008438935.1**	0	0.18126582	1	1
**XP_008461399.1**	0	0.19573537	1	1
**XP_008447227.1**	0	0.16566404	1	1
**XP_008453618.1**	0	0.16934721	1	1
**XP_008439346.1**	0	0.16184448	1	1
**XP_008446078.1**	0	0.6	1	1
**XP_008438316.1**	0	1	1	1
**XP_008464872.1**	0	0.14690193	1	1
**XP_008451731.1**	0	0.15061001	1	1
**XP_008455343.1**	0	0.12681544	1	1
**XP_008459689.1**	0	0.15667396	1	1
**XP_008440421.1**	0	0.14564687	1	1
**XP_008452434.1**	0	0.66666667	1	1
**XP_008466746.1**	0	0.12370422	1	1

**XP_008451887.1**	0	0.1645977	1	1
**XP_008442324.1**	0	0.15531453	1	1
**XP_008451032.1**	0	0.17137386	1	1
**XP_008450882.1**	0	1	1	1
**XP_008443629.1**	0	1	1	1
**XP_008447157.1**	0	0.15982143	1	1
**XP_008460157.1**	0	0.66666667	1	1
**XP_008440277.1**	0	1	1	1
**XP_008449553.1**	0	0.20146314	1	1
**XP_008438271.1**	0	0.66666667	1	1
**XP_008453176.1**	0	0.66666667	1	1
**XP_008452006.1**	0	0.18349564	1	1
**XP_008466262.1**	0	0.12409012	1	1
**XP_008440059.1**	0	0.15524718	1	1
**XP_008459885.1**	0	0.6	1	1
**XP_008449479.1**	0	0.15975011	1	1
**XP_008459400.1**	0	0.18340164	1	1
**XP_008439796.1**	0	0.20146314	1	1
**XP_008459116.1**	0	0.14452967	1	1
**XP_008437783.1**	0	0.18191057	1	1
**XP_008449445.1**	0	0.66666667	1	1
**XP_008445145.1**	0	0.19113721	1	1
**XP_008461417.1**	0	0.66666667	1	1
**XP_008438077.1**	0	0.6	1	1
**XP_008438515.1**	0	0.2043379	1	1
**XP_008444874.1**	0	0.15061001	1	1
**XP_008447454.1**	0	0.14211989	1	1

40 DAP fruit

Figure S6 40 DAP fruit network protein interaction (STRING software). The color are represented by functional enrichment in the network (legend). Nodes represent related proteins. Edges represent protein–protein associations.

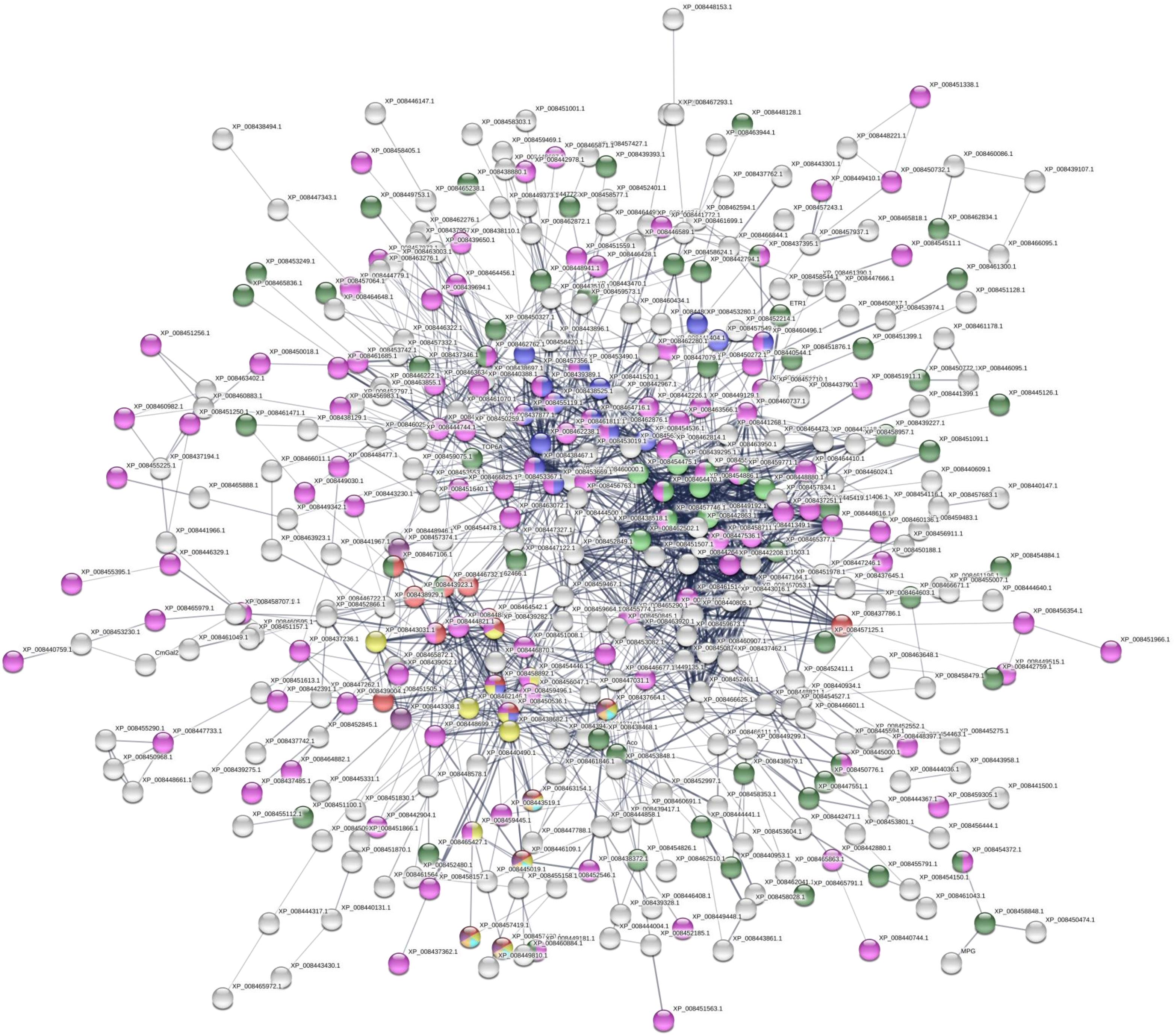

Legend:

KEGG Pathways

pathway	description	count in gene set	false discovery rate
cmo04141	Protein processing in endoplasmic reticulum	21 of 168	0.00081
cmo03040	Spliceosome	17 of 150	0.0083
cmo03008	Ribosome biogenesis in eukaryotes	10 of 75	0.0382

UniProt Keywords

keyword	description	count in gene set	false discovery rate
KW-0346	Stress response	17 of 52	7.39e-08
KW-0175	Coiled coil	179 of 3077	2.59e-06
KW-0479	Metal-binding	91 of 1662	0.0482

PFAM Protein Domains

domain	description	count in gene set	false discovery rate
PF00011	Hsp20/alpha crystallin family	9 of 35	0.0210

INTERPRO Protein Domains and Features

domain	description	count in gene set	false discovery rate
IPR031107	Small heat shock protein HSP20	9 of 25	0.0036
IPR008978	HSP20-like chaperone	11 of 47	0.0050
IPR002068	Alpha crystallin/Hsp20 domain	9 of 35	0.0117

Obs.: 3 genes of “Protein processing in endoplasmic reticulum”, 1 gene of “Stress response”, 61 genes of “Coiled coil”, 36 of “Metal binding”, 2 genes of “Hps20/alpha crystalline family”, 2 genes of “Small heat shock protein HSP20”, 2 genes of “HSP20-like chaperone”, 2 genes of “Alpha crystalline/Hsp20 domain” are disconnected nodes in the network.

Table S8 40 DAP network profile (Cytoscape software)

Name/RefseqID	BetweennessCentrality	ClosenessCentrality	Degree	NumberOfDirectedEdges
**XP_008454886.1**	0.04353076	0.36182336	47	47
**XP_008453669.1**	0.16161075	0.40021008	45	45
**XP_008464470.1**	0.02344788	0.3621673	41	41
**XP_008455669.1**	0.02811092	0.3544186	39	39
**XP_008447164.1**	0.01428812	0.35018382	39	39
**XP_008467053.1**	0.01374343	0.34986226	38	38
**XP_008442863.1**	0.01146916	0.34762774	37	37
**XP_008442648.1**	0.02225901	0.35310473	37	37
**XP_008462502.1**	0.0161428	0.33957219	35	35
**XP_008442208.1**	0.00724988	0.34231806	35	35
**XP_008439295.1**	0.01451041	0.34794521	35	35
**XP_008449192.1**	0.04602161	0.35875706	34	34
**XP_008457746.1**	0.01059097	0.33538732	33	33
**XP_008452849.1**	0.04547195	0.37170732	33	33
**XP_008437251.1**	0.00299694	0.32731959	32	32
**XP_008462876.1**	0.06736557	0.36182336	32	32
**XP_008461503.1**	0.00523682	0.33896797	32	32
**XP_008454475.1**	0.02589995	0.33568282	31	31
**XP_008438467.1**	0.04144616	0.35212569	31	31
**XP_008440805.1**	0.01247235	0.34448463	31	31
**XP_008454581.1**	0.01247235	0.34448463	31	31
**XP_008447536.1**	0.03704247	0.3557423	30	30
**XP_008465377.1**	0.00259549	0.33130435	29	29
**XP_008460907.1**	0.00967448	0.33716814	28	28
**XP_008462762.1**	0.05363781	0.34699454	28	28
**XP_008465290.1**	0.03363968	0.34201077	28	28
**XP_008461514.1**	0.0182462	0.34448463	27	27
**XP_008459771.1**	9.35E+00	0.32536294	26	26
**XP_008463920.1**	0.03203773	0.35376045	26	26
**XP_008437462.1**	0.0070598	0.33333333	25	25
**XP_008438779.1**	0.07888137	0.35909519	25	25
**XP_008438682.1**	0.02575229	0.33509235	25	25
**XP_008454446.1**	0.02308093	0.33866667	24	24
**XP_008443016.1**	0.01402858	0.32425532	23	23
**XP_008438518.1**	0.01137911	0.35180055	23	23
**XP_008460737.1**	0.00409518	0.31856187	23	23
**XP_008450536.1**	0.02424612	0.33391762	23	23
**XP_008450874.1**	0.0066178	0.32480818	22	22
**XP_008448880.1**	0.0010395	0.32233503	22	22
**XP_008441349.1**	0.00172675	0.31829574	22	22
**XP_008448616.1**	7.71E+00	0.30407023	22	22
**XP_008439282.1**	0.03921441	0.34604905	22	22
**XP_008459467.1**	0.03956305	0.36079545	22	22
**XP_008462146.1**	0.01932258	0.31856187	22	22
**XP_008456763.1**	0.04276626	0.34324324	22	22
**XP_008458892.1**	0.02864363	0.33391762	22	22
**XP_008466625.1**	0.02885188	0.31618257	21	21

**XP_008439389.1**	0.01258917	0.33627538	21	21
**XP_008444821.1**	0.06753463	0.34355275	21	21
**XP_008461070.1**	0.03733943	0.33987511	21	21
**XP_008449135.1**	0.03923533	0.33509235	21	21
**XP_008458420.1**	0.00814082	0.31203931	20	20
**XP_008462238.1**	0.00455571	0.31178396	20	20
**XP_008453490.1**	0.00714464	0.32987013	20	20
**XP_008464716.1**	0.01879395	0.32260796	20	20
**XP_008448895.1**	0.02209349	0.33746678	20	20
**XP_008451008.1**	0.01734303	0.34636364	19	19
**XP_008460000.1**	0.02097785	0.32124789	19	19
**XP_008452461.1**	0.00163808	0.31487603	18	18
**XP_008456305.1**	0.01342763	0.29788898	18	18
**XP_008446677.1**	0.02544184	0.33072917	18	18
**XP_008442118.1**	0.0068696	0.31051345	17	17
**XP_008442967.1**	0.00346616	0.32206255	17	17
**XP_008445419.1**	2.79E+00	0.31102041	15	15
**XP_008461811.1**	0.02415955	0.33421053	15	15
**XP_008446732.1**	0.03101555	0.33657244	15	15
**XP_008438468.1**	0.03110876	0.31229508	15	15
**XP_008441520.1**	0.00528569	0.31306491	14	14
**XP_008443031.1**	0.00894934	0.29835552	14	14
**XP_008464410.1**	2.16E-02	0.30023641	14	14
**XP_008437664.1**	0.01047539	0.29150727	14	14
**XP_008443510.1**	0.02773965	0.32260796	14	14
**XP_008451507.1**	0.00546093	0.31539735	14	14
**XP_008437786.1**	0.01566662	0.29017517	14	14
**XP_008448699.1**	0.00746309	0.29443586	13	13
**XP_008455119.1**	3.21E+00	0.3062701	12	12
**XP_008438525.1**	3.21E+00	0.3062701	12	12
**XP_008447031.1**	0.02313673	0.31332237	12	12
**XP_008443308.1**	0.00568491	0.2912844	12	12
**XP_008463566.1**	0.03556638	0.33627538	12	12
**XP_008453860.1**	0.00736016	0.32070707	11	11
**XP_008453019.1**	0.01631971	0.30023641	11	11
**XP_008446322.1**	0.01655297	0.29976397	11	11
**XP_008452997.1**	0.01433803	0.30142405	11	11
**XP_008451505.1**	0.0043249	0.28776435	10	10
**XP_008453367.1**	0.00785714	0.31051345	10	10
**XP_008458957.1**	1.49E-02	0.29106188	10	10
**XP_008453848.1**	0.01040009	0.28689759	10	10
**XP_008453082.1**	0.03187766	0.31882845	10	10
**XP_008462814.1**	0.00556374	0.28776435	10	10
**XP_008449299.1**	0.03327329	0.2881997	10	10
**XP_008441268.1**	0.00104533	0.30358566	10	10
**XP_008454536.1**	7.79E+00	0.30900243	9	9
**XP_008457549.1**	0.00479225	0.29512006	9	9
**XP_008447327.1**	0.01549364	0.32288136	9	9
**XP_008464542.1**	0.01343467	0.31618257	9	9

**XP_008459496.1**	0.00783295	0.30238095	9	9
**XP_008443896.1**	3.07E-02	0.28411633	9	9
**XP_008459445.1**	0.01363207	0.28055965	9	9
**XP_008448821.1**	0.0143627	0.27469358	9	9
**XP_008448941.1**	0.02433552	0.27973568	9	9
**XP_008450327.1**	0.00940392	0.29240215	9	9
**XP_008437346.1**	0.01102551	0.2881997	9	9
**XP_008438929.1**	0.04525324	0.31803005	9	9
**XP_008463072.1**	0.00330337	0.30358566	8	8
**XP_008451978.1**	0.02318902	0.30651649	8	8
**Aco**	0.01195922	0.28159645	8	8
**XP_008437877.1**	0.00678478	0.32179054	8	8
**XP_008440388.1**	0.00677319	0.30701048	8	8
**XP_008437161.1**	7.25E+00	0.29083969	8	8
**XP_008464473.1**	0.02110297	0.3	8	8
**XP_008446109.1**	0.00776202	0.25883152	8	8
**XP_008447122.1**	0.00454145	0.30023641	8	8
**XP_008459673.1**	0.00470834	0.31127451	8	8
**XP_008443470.1**	0.02335797	0.28432836	8	8
**XP_008439417.1**	0.01092416	0.29835552	8	8
**XP_008452866.1**	0.02979693	0.27528902	7	7
**XP_008462280.1**	7.71E+00	0.28863636	7	7
**XP_008450259.1**	7.18E+00	0.29765625	7	7
**XP_008462466.1**	0.00473241	0.31026059	7	7
**XP_008443923.1**	0.006095	0.28180473	7	7
**XP_008457356.1**	0.00200678	0.31989924	7	7
**XP_008438697.1**	0.00162804	0.30286169	7	7
**XP_008451640.1**	0.01529558	0.28841787	7	7
**XP_008439052.1**	0.0028146	0.29262673	7	7
**XP_008447079.1**	0.01192176	0.28222222	7	7
**XP_008448067.1**	0.00691142	0.31409728	7	7
**XP_008440934.1**	0.0108573	0.2962675	7	7
**XP_008450272.1**	0.01248094	0.26006826	7	7
**XP_008457125.1**	1.02E+00	0.28411633	7	7
**XP_008460496.1**	0.00830413	0.27789934	6	6
**XP_008463154.1**	0	0.28180473	6	6
**XP_008447246.1**	0.02139606	0.2733142	6	6
**XP_008449129.1**	0.00138572	0.27749454	6	6
**XP_008442226.1**	0.00770515	0.30238095	6	6
**XP_008448578.1**	0.01479436	0.26887791	6	6
**XP_008457937.1**	0.02623067	0.2555332	6	6
**XP_008466111.1**	0.00575218	0.26239669	6	6
**XP_008446601.1**	3.60E-07	0.26718093	6	6
**XP_008439004.1**	0.01079686	0.25900748	6	6
**TOP6A**	8.40E+00	0.28885519	6	6
**XP_008454826.1**	0.02104956	0.26830986	6	6
**XP_008458711.1**	0.00856982	0.27608696	6	6
**XP_008463634.1**	0.0059927	0.28929385	6	6
**XP_008462594.1**	0.01632311	0.27292264	6	6

**XP_008451559.1**	0.00991985	0.30214116	6	6
**XP_008453280.1**	4.26E+00	0.29083969	6	6
**XP_008467106.1**	0.00184639	0.26868829	5	5
**XP_008459664.1**	0.00212491	0.28222222	5	5
**XP_008461846.1**	3.14E+00	0.28689759	5	5
**XP_008439427.1**	0.00567027	0.27688953	5	5
**XP_008454527.1**	0.00459114	0.27608696	5	5
**XP_008444858.1**	0.01273482	0.25016415	5	5
**XP_008461699.1**	0.01246367	0.25918367	5	5
**XP_008439650.1**	0.00700149	0.28222222	5	5
**XP_008450845.1**	0.0021339	0.28097345	5	5
**XP_008452546.1**	0.00231924	0.22993361	5	5
**XP_008444744.1**	0.00178932	0.27729258	5	5
**XP_008455774.1**	0.00375391	0.28014706	5	5
**XP_008464603.1**	0.00668358	0.24869452	5	5
**XP_008465872.1**	0.00677386	0.25760649	5	5
**XP_008452710.1**	0.00675184	0.254	4	4
**XP_008453742.1**	0	0.28432836	4	4
**XP_008452411.1**	0.0116763	0.24007561	4	4
**XP_008453553.1**	6.23E+00	0.26662001	4	4
**XP_008462810.1**	0.01063157	0.30750605	4	4
**XP_008439694.1**	2.77E+00	0.29195402	4	4
**XP_008464456.1**	2.77E+00	0.29195402	4	4
**XP_008456047.1**	8.34E+00	0.26167582	4	4
**XP_008466844.1**	0.0068165	0.24098672	4	4
**XP_008439190.1**	0.01602206	0.26275862	4	4
**XP_008448477.1**	0.00179125	0.2845407	4	4
**XP_008444367.1**	0.00526316	0.18676471	4	4
**XP_008452214.1**	0.00101995	0.27608696	4	4
**XP_008451091.1**	0	0.2602459	4	4
**XP_008447262.1**	0.00606321	0.26239669	4	4
**XP_008440544.1**	0.00528996	0.25760649	4	4
**XP_008463950.1**	0.00352835	0.28180473	4	4
**XP_008455158.1**	0.01224253	0.24267516	4	4
**XP_008437194.1**	0.00184695	0.21525424	4	4
**XP_008459075.1**	7.75E+00	0.27098151	4	4
**XP_008447733.1**	0.01566515	0.19598765	4	4
**XP_008442794.1**	0.00337536	0.28646617	4	4
**XP_008458624.1**	0.00337536	0.28646617	4	4
**XP_008463402.1**	0.00820941	0.26662001	4	4
**XP_008460883.1**	0.00820941	0.26662001	4	4
**XP_008443744.1**	0.00667191	0.27002126	4	4
**XP_008463276.1**	5.81E-02	0.2602459	4	4
**XP_008437957.1**	5.81E-02	0.2602459	4	4
**XP_008464495.1**	6.30E-05	0.26868829	4	4
**XP_008463855.1**	3.30E+00	0.25656566	3	3
**XP_008461685.1**	0.00132993	0.27830533	3	3
**ETR1**	8.05E+00	0.22965642	3	3
**XP_008458353.1**	0.00524934	0.22597865	3	3

**XP_008444441.1**	0.01047106	0.24252069	3	3
**XP_008458157.1**	0.00524934	0.25165125	3	3
**XP_008451876.1**	0.01099037	0.22855429	3	3
**XP_008440490.1**	0	0.28306092	3	3
**XP_008441404.1**	4.05E-01	0.25673854	3	3
**XP_008447551.1**	0.00350831	0.22869148	3	3
**XP_008445000.1**	0.00350831	0.22869148	3	3
**XP_008450776.1**	0.00350831	0.22869148	3	3
**XP_008452185.1**	0.00524934	0.21202003	3	3
**XP_008446329.1**	0.00607842	0.2106136	3	3
**XP_008452845.1**	0.00524934	0.21947005	3	3
**XP_008445019.1**	0.00126514	0.23388582	3	3
**XP_008457374.1**	4.06E+00	0.29976397	3	3
**XP_008437395.1**	3.89E+00	0.23062954	3	3
**XP_008446589.1**	0	0.26736842	3	3
**XP_008461390.1**	0.00776706	0.26699369	3	3
**XP_008439227.1**	1.73E-06	0.27117438	3	3
**XP_008450968.1**	0	0.16408269	3	3
**XP_008448661.1**	0	0.16408269	3	3
**XP_008455290.1**	0	0.16408269	3	3
**XP_008458848.1**	1	1	3	3
**XP_008446870.1**	0	0.26718093	3	3
**XP_008437236.1**	4.36E-01	0.25587643	3	3
**XP_008444500.1**	3.22E+00	0.2803532	3	3
**XP_008438679.1**	1.73E+00	0.24967235	3	3
**XP_008460434.1**	0	0.26060192	3	3
**XP_008460254.1**	0.00454508	0.29017517	3	3
**XP_008453230.1**	0.00525625	0.15191388	3	3
**XP_008457834.1**	0.00554009	0.26348548	3	3
**XP_008458707.1**	0.02083851	0.21697039	3	3
**XP_008466671.1**	0.00536491	0.21858864	3	3
**XP_008466825.1**	1.39E+00	0.26569038	3	3
**XP_008442880.1**	0.01048487	0.22438163	3	3
**XP_008460691.1**	0	0.27002126	3	3
**XP_008465238.1**	0.00524934	0.26078029	3	3
**XP_008441967.1**	0.0010356	0.26275862	3	3
**XP_008449342.1**	0.01077204	0.27021277	3	3
**XP_008460982.1**	3.45E-06	0.21154914	3	3
**XP_008456983.1**	4.19E+00	0.26275862	3	3
**XP_008443519.1**	0.0010255	0.25989086	3	3
**XP_008448221.1**	0.00261776	0.20396146	3	3
**XP_008450732.1**	0.00261776	0.20396146	3	3
**XP_008463648.1**	0.00560795	0.26868829	3	3
**XP_008442391.1**	0.0055198	0.24660194	3	3
**XP_008456354.1**	0.00524934	0.22544379	3	3
**XP_008451830.1**	0	0.27059659	3	3
**XP_008442904.1**	0	0.27059659	3	3
**XP_008457973.1**	0	0.26330339	3	3

**XP_008462276.1**	0	0.26330339	3	3
**XP_008438110.1**	0	0.2881997	3	3
**XP_008449030.1**	7.95E+00	0.2651357	3	3
**XP_008437645.1**	0	0.23489519	2	2
**XP_008458028.1**	0	0.19538462	2	2
**XP_008438372.1**	3.96E-02	0.22746269	2	2
**XP_008453974.1**	0.00524934	0.18630807	2	2
**XP_008459573.1**	0	0.28929385	2	2
**XP_008443861.1**	0	0.19538462	2	2
**XP_008442471.1**	0.00524934	0.26569038	2	2
**XP_008460136.1**	0	0.26587579	2	2
**XP_008465427.1**	2.46E-01	0.21947005	2	2
**XP_008460595.1**	0.00524934	0.24175127	2	2
**XP_008455007.1**	3.60E-07	0.25989086	2	2
**XP_008461178.1**	0	1	2	2
**XP_008450772.1**	0	1	2	2
**XP_008450188.1**	0	0.23489519	2	2
**XP_008446095.1**	0	1	2	2
**XP_008453801.1**	0.00524934	0.24068225	2	2
**XP_008447788.1**	7.88E-02	0.24950884	2	2
**XP_008443230.1**	0.00122793	0.27509025	2	2
**XP_008463923.1**	9.15E+00	0.24236641	2	2
**XP_008465888.1**	0.00524934	0.21308725	2	2
**XP_008463003.1**	0	0.2555332	2	2
**XP_008457420.1**	0	0.22112594	2	2
**XP_008457419.1**	0	0.22112594	2	2
**XP_008445594.1**	0	0.2555332	2	2
**XP_008454478.1**	0	0.27021277	2	2
**XP_008440953.1**	0	0.24596514	2	2
**XP_008451250.1**	3.19E+00	0.19558522	2	2
**XP_008450018.1**	0.00390607	0.22557726	2	2
**XP_008461196.1**	0.00165231	0.26587579	2	2
**XP_008465979.1**	0.00522172	0.17870544	2	2
**XP_008451256.1**	3.45E-06	0.21084671	2	2
**XP_008462834.1**	0.16666667	0.75	2	2
**XP_008460086.1**	0.16666667	0.75	2	2
**XP_008466095.1**	0.16666667	0.75	2	2
**XP_008462872.1**	0	0.23302752	2	2
**XP_008451613.1**	0.02083161	0.24267516	2	2
**XP_008451399.1**	0.00524934	0.23562152	2	2
**XP_008440131.1**	1	1	2	2
**XP_008453604.1**	0	0.23827392	2	2
**XP_008437762.1**	3.45E-06	0.22306792	2	2
**XP_008464648.1**	0	0.27253219	2	2
**XP_008444779.1**	0	0.27253219	2	2
**XP_008444004.1**	0	0.21190211	2	2
**XP_008448397.1**	0	0.24918247	2	2
**XP_008446428.1**	5.11E+00	0.23331292	2	2

**XP_008466011.1**	2.38E+00	0.23021148	2	2
**XP_008454116.1**	5.74E+00	0.23842303	2	2
**XP_008456911.1**	0	0.26587579	2	2
**XP_008449373.1**	7.08E-05	0.23887147	2	2
**XP_008459469.1**	3.75E-02	0.25181758	2	2
**XP_008458479.1**	0.00125962	0.23007246	2	2
**XP_008441966.1**	0.00163846	0.2092257	2	2
**XP_008467293.1**	0.00524934	0.20616883	2	2
**XP_008461471.1**	1.89E+00	0.26532033	2	2
**XP_008452480.1**	9.90E+00	0.22023121	2	2
**XP_008439107.1**	0.16666667	0.75	2	2
**CmGal2**	0.00522172	0.17870544	2	2
**XP_008452797.1**	7.54E-02	0.26983003	2	2
**XP_008451338.1**	0	0.16955941	2	2
**XP_008446024.1**	3.99E-02	0.24205845	2	2
**XP_008441406.1**	3.99E-02	0.24205845	2	2
**XP_008447343.1**	0.00524934	0.23104912	2	2
**XP_008450952.1**	0.01047106	0.25795531	2	2
**XP_008444317.1**	0.00524934	0.20539084	2	2
**XP_008449515.1**	0	0.22531047	2	2
**XP_008447722.1**	0	0.24407431	1	1
**XP_008441772.1**	0	0.2557047	1	1
**XP_008462041.1**	0	0.18441433	1	1
**XP_008449410.1**	0	0.20363442	1	1
**XP_008465818.1**	0	0.20363442	1	1
**XP_008451911.1**	0	0.23090909	1	1
**XP_008452552.1**	0	0.19369598	1	1
**XP_008451563.1**	0	0.17501148	1	1
**XP_008443301.1**	0	0.19428863	1	1
**XP_008451866.1**	0	0.21202003	1	1
**XP_008461300.1**	0	0.1571134	1	1
**XP_008446722.1**	0	0.26532033	1	1
**XP_008455791.1**	0	0.21003308	1	1
**XP_008464882.1**	0	0.20583468	1	1
**XP_008461049.1**	0	0.19478528	1	1
**XP_008454511.1**	0	0.21084671	1	1
**XP_008441399.1**	0	0.26587579	1	1
**XP_008456444.1**	0	0.15743802	1	1
**MPG**	0	0.6	1	1
**XP_008450474.1**	0	0.6	1	1
**XP_008437742.1**	0	0.20796943	1	1
**XP_008439328.1**	0	0.20021019	1	1
**XP_008446408.1**	0	0.20021019	1	1
**XP_008450817.1**	0	0.2049489	1	1
**XP_008455225.1**	0	0.17573801	1	1
**XP_008449181.1**	0	0.19538462	1	1
**XP_008440759.1**	0	0.13192521	1	1
**XP_008458544.1**	0	0.20650407	1	1
**XP_008459483.1**	0	0.21476888	1	1

**XP_008449810.1**	0	0.19538462	1	1
**XP_008460884.1**	0	0.20572354	1	1
**XP_008449753.1**	0	0.25778078	1	1
**XP_008437485.1**	0	0.18005671	1	1
**XP_008454884.1**	0	0.17946302	1	1
**XP_008454150.1**	0	0.18334937	1	1
**XP_008454372.1**	0	0.6	1	1
**XP_008457683.1**	0	1	1	1
**XP_008440147.1**	0	1	1	1
**XP_008449448.1**	0	0.21166667	1	1
**XP_008445331.1**	0	0.2257109	1	1
**XP_008442978.1**	0	0.25181758	1	1
**XP_008451870.1**	0	0.66666667	1	1
**XP_008458577.1**	0	0.22151163	1	1
**XP_008461564.1**	0	0.24175127	1	1
**XP_008443790.1**	0	0.23104912	1	1
**XP_008444036.1**	0	1	1	1
**XP_008443958.1**	0	1	1	1
**XP_008459305.1**	0	1	1	1
**XP_008441500.1**	0	1	1	1
**XP_008455112.1**	0	1	1	1
**XP_008451100.1**	0	1	1	1
**XP_008457332.1**	0	0.28603604	1	1
**XP_008446222.1**	0	0.28603604	1	1
**XP_008437362.1**	0	0.20116156	1	1
**XP_008463944.1**	0	0.21452703	1	1
**XP_008454463.1**	0	0.19369598	1	1
**XP_008447666.1**	0	0.20650407	1	1
**XP_008465791.1**	0	0.25115359	1	1
**XP_008457246.1**	0	0.21273032	1	1
**XP_008439275.1**	0	0.19792208	1	1
**XP_008446147.1**	0	0.20695274	1	1
**XP_008451966.1**	0	0.18405797	1	1
**XP_008457243.1**	0	0.26587579	1	1
**XP_008445275.1**	0	0.256393	1	1
**XP_008465863.1**	0	0.256393	1	1
**XP_008438129.1**	0	0.22398589	1	1
**XP_008448946.1**	0	0.24423077	1	1
**XP_008448153.1**	0	0.17100539	1	1
**XP_008448687.1**	0	0.21871412	1	1
**XP_008457427.1**	0	0.21871412	1	1
**XP_008444640.1**	0	0.19926778	1	1
**XP_008465836.1**	0	0.26439972	1	1
**XP_008453249.1**	0	0.26439972	1	1
**XP_008465871.1**	0	0.21871412	1	1
**XP_008438880.1**	0	0.21871412	1	1
**XP_008438494.1**	0	0.18777723	1	1
**XP_008439393.1**	0	0.22151163	1	1
**XP_008458303.1**	0	0.24407431	1	1

**XP_008451001.1**	0	0.24407431	1	1
**XP_008452401.1**	0	0.25065789	1	1
**XP_008440609.1**	0	0.20865279	1	1
**XP_008451128.1**	0	0.19078618	1	1
**XP_008462510.1**	0	0.24190476	1	1
**XP_008457064.1**	0	0.22451385	1	1
**XP_008458405.1**	0	0.22023121	1	1
**XP_008461043.1**	0	0.19409068	1	1
**XP_008465972.1**	0	0.1704698	1	1
**XP_008442759.1**	0	0.21190211	1	1
**XP_008443430.1**	0	0.66666667	1	1
**XP_008448128.1**	0	0.21452703	1	1
**XP_008451157.1**	0	0.25198413	1	1
**XP_008455395.1**	0	0.17405208	1	1
**XP_008445126.1**	0	0.23708774	1	1
**XP_008440744.1**	0	0.18334937	1	1
