# Supplementary material for: Transcriptome profiling of non-climacteric ‘yellow’ melon during ripening: insights on sugar metabolism
Source: BMC Genomics. 2020 Mar 30;21:262. doi: 10.1186/s12864-020-6667-0 (PMC7106763; doi:10.1186/s12864-020-6667-0)
Supplement: Supplementary file 9 — Additional File 9: Tables S9, S10, S11, S12. Characteristics of PPI network interaction of sugar and associated pathways. [file 12864_2020_6667_MOESM9_ESM.pdf]

Table S9 Subnetwork profile of young fruit (10 DAP) generated by STRING and Cytoscape analyses.

ID name	BetweennessCentrality	ClosenessCentrality	Degree	NumberOfDirectedEdges
XP_008443206.1	0.48676176	0.45121951	10	10
XP_008463167.1	0.16653082	0.38947368	9	9
NP_001284469.1	0.18057343	0.37373737	9	9
XP_008444380.1	0.1022308	0.41573034	8	8
XP_008445911.1	0.16947304	0.42045455	8	8
XP_008452100.1	0.19270699	0.40217391	6	6
XP_008457154.1	0.09373659	0.37373737	6	6
XP_008440310.1	0.12805305	0.39361702	5	5
XP_008451468.1	0.00209138	0.33333333	5	5
XP_008452914.1	0.07100672	0.37	5	5
XP_008446229.1	0.05405405	0.30327869	4	4
XP_008438007.1	0.25075075	0.28244275	4	4
XP_008467118.1	0.05934506	0.31623932	4	4
XP_008443553.1	0.001716	0.33035714	4	4
XP_008460758.1	0.001716	0.33035714	4	4
XP_008460145.1	0	0.30081301	3	3
XP_008459427.1	0.10660661	0.22699387	3	3
XP_008458374.1	0.00525526	0.33636364	3	3
XP_008438969.1	0.00775776	0.30327869	3	3
XP_008446196.1	0.27927928	0.35238095	3	3
XP_008465523.1	2.15E-01	0.3592233	3	3
XP_008453253.1	0.05405405	0.30081301	3	3
XP_008441351.1	0.03878879	0.35576923	3	3
XP_008442968.1	0	0.31355932	3	3
XP_008459280.1	7.51E+00	0.22155689	2	2
XP_008450452.1	0.02627628	0.27819549	2	2
XP_008462107.1	0	0.31623932	2	2
XP_008453064.1	0.02627628	0.27819549	2	2
XP_008437557.1	0	0.18592965	1	1
XP_008465204.1	0	0.18592965	1	1
XP_008451740.1	0	0.2327044	1	1
XP_008466126.1	0	0.24183007	1	1
XP_008439346.1	0	0.23417722	1	1
XP_008454693.1	0	0.28461538	1	1
XP_008449737.1	0	0.31355932	1	1
XP_008460901.1	0	0.22155689	1	1
XP_008460902.1	0	0.22155689	1	1
XP_008437427.1	0	0.28461538	1	1

Table S10 STRING interactions of subnetwork young fruit (10 DAP).

#node1	node2	node1_string_internal_id	node2_string_internal_id	node1_external_id	node2_external_id	neighborhood_on_chromosome	gene_fusion	phylogenetic_cooccurrence	homology	coexpression	experimentally_determined_interaction	database_annotated	automated_textmining	combined_score
XP_008457154.1	XP_008442968.1	530584	521376	3656.XP_008457154.1	3656.XP_008442968.1	0.075	0	0.306	0	0	0.154	0.9	0.455	0.965
XP_008463167.1	XP_008442968.1	534543	521376	3656.XP_008463167.1	3656.XP_008442968.1	0.075	0	0.422	0	0	0.154	0.9	0.141	0.954
NP_001284469.1	XP_008463167.1	537263	534543	3656.XP_008467212.1	3656.XP_008463167.1	0	0	0	0	0	0	0.65	0.874	0.954
XP_008451468.1	XP_008445911.1	526835	523289	3656.XP_008451468.1	3656.XP_008445911.1	0	0	0	0	0	0	0.9	0.495	0.947
XP_008453253.1	XP_008451740.1	528014	527018	3656.XP_008453253.1	3656.XP_008451740.1	0.165	0	0	0	0.111	0	0.9	0.355	0.945
XP_008445911.1	XP_008451468.1	532919	526835	3656.XP_008460758.1	3656.XP_008451468.1	0	0	0	0.85	0	0	0.9	0.565	0.908
XP_008451468.1	XP_008443553.1	526835	521754	3656.XP_008451468.1	3656.XP_008443553.1	0	0	0	0.931	0	0	0.9	0.715	0.904
NP_001284469.1	XP_008451468.1	537263	526835	3656.XP_008467212.1	3656.XP_008451468.1	0	0	0	0	0	0	0.9	0.042	0.9
XP_008445911.1	XP_008443553.1	523289	521754	3656.XP_008445911.1	3656.XP_008443553.1	0	0	0	0	0	0	0.65	0.567	0.841
XP_008445911.1	XP_008445911.1	532919	523289	3656.XP_008460758.1	3656.XP_008445911.1	0	0	0	0	0	0	0.65	0.518	0.824
XP_008452914.1	XP_008444380.1	527786	522291	3656.XP_008452914.1	3656.XP_008444380.1	0	0	0	0	0	0	0	0.82	0.82
XP_008446229.1	XP_008439346.1	523509	519034	3656.XP_008446229.1	3656.XP_008439346.1	0	0.007	0.443	0.579	0	0	0.65	0.627	0.785
XP_008463167.1	XP_008457154.1	534543	530584	3656.XP_008463167.1	3656.XP_008457154.1	0	0	0.356	0.594	0	0	0.65	0.622	0.771
XP_008445911.1	XP_008443206.1	523289	521518	3656.XP_008445911.1	3656.XP_008443206.1	0	0	0.299	0	0	0	0	0.629	0.728
XP_008463167.1	XP_008440310.1	534543	519647	3656.XP_008463167.1	3656.XP_008440310.1	0	0	0	0	0	0	0	0.707	0.707
NP_001284469.1	XP_008442968.1	537263	521376	3656.XP_008467212.1	3656.XP_008442968.1	0.129	0	0	0	0	0	0.65	0.105	0.703
NP_001284469.1	XP_008457154.1	537263	530584	3656.XP_008467212.1	3656.XP_008457154.1	0	0	0	0	0	0	0	0.688	0.688
XP_008457154.1	XP_008446229.1	530584	523509	3656.XP_008457154.1	3656.XP_008446229.1	0.055	0	0	0	0.072	0.045	0.65	0.08	0.681
XP_008463167.1	XP_008446229.1	534543	523509	3656.XP_008463167.1	3656.XP_008446229.1	0.055	0	0	0	0.072	0.045	0.65	0.08	0.681

NP_001284469.1	XP_008445911.1	537263	523289	3656.XP_008467212.1	3656.XP_008445911.1	0	0	0	0	0	0	0.65	0.115	0.677
NP_001284469.1	XP_008443553.1	537263	521754	3656.XP_008467212.1	3656.XP_008443553.1	0	0	0	0	0	0	0.65	0.11	0.675
NP_001284469.1	XP_008453064.1	537263	527877	3656.XP_008467212.1	3656.XP_008453064.1	0	0	0	0	0	0	0.65	0.045	0.651
NP_001284469.1	BG1	537263	526192	3656.XP_008467212.1	3656.XP_008450452.1	0	0	0	0	0	0	0.65	0.045	0.651
XP_008459280.1	BG1	531977	526192	3656.XP_008459280.1	3656.XP_008450452.1	0	0	0	0	0	0	0.65	0	0.65
NP_001284469.1	XP_008445911.1	537263	532919	3656.XP_008467212.1	3656.XP_008460758.1	0	0	0	0	0	0	0.65	0	0.65
XP_008459280.1	XP_008453064.1	531977	527877	3656.XP_008459280.1	3656.XP_008453064.1	0	0	0	0	0	0	0.65	0	0.65
XP_008452100.1	XP_008443206.1	527257	521518	3656.XP_008452100.1	3656.XP_008443206.1	0.054	0	0	0	0.115	0	0	0.578	0.615
XP_008458374.1	XP_008443206.1	531389	521518	3656.XP_008458374.1	3656.XP_008443206.1	0.084	0	0	0	0.063	0	0	0.579	0.607
XP_008462107.1	XP_008443206.1	533812	521518	3656.XP_008462107.1	3656.XP_008443206.1	0	0	0	0	0	0	0.6	0.056	0.606
XP_008446196	XP_008443206	523487	521518	3656.XP_008446196.	3656.XP_008443206.	0	0	0	0	0	0	0.6	0.056	0.606
.1			.1				1				1			
XP_008454693.1	XP_008440310.1	528953	519647	3656.XP_008454693.1	3656.XP_008440310.1	0	0	0	0	0	0	0	0.606	0.606
XP_008463167.1	XP_008460145.1	534543	532534	3656.XP_008463167.1	3656.XP_008460145.1	0	0	0	0	0	0	0.6	0	0.6
XP_008458374.1	XP_008446196.1	531389	523487	3656.XP_008458374.1	3656.XP_008446196.1	0	0	0	0	0	0	0.6	0	0.6
XP_008460145.1	XP_008446229.1	532534	523509	3656.XP_008460145.1	3656.XP_008446229.1	0	0	0	0	0	0	0.6	0	0.6
XP_008460145.1	XP_008457154.1	532534	530584	3656.XP_008460145.1	3656.XP_008457154.1	0	0	0	0	0	0	0.6	0	0.6
XP_008462107.1	XP_008458374.1	533812	531389	3656.XP_008462107.1	3656.XP_008458374.1	0	0	0	0	0	0	0.6	0	0.6
XP_008451468.1	XP_008444380.1	526835	522291	3656.XP_008451468.1	3656.XP_008444380.1	0	0	0	0	0	0	0	0.592	0.592
XP_008465523.1	XP_008445911.1	536060	523289	3656.XP_008465523.1	3656.XP_008445911.1	0	0	0	0	0.045	0.322	0	0.42	0.591
XP_008465523.1	XP_008444380.1	536060	522291	3656.XP_008465523.1	3656.XP_008444380.1	0	0	0	0	0.045	0.322	0	0.42	0.591
XP_008463167.1	XP_008452914.1	534543	527786	3656.XP_008463167.1	3656.XP_008452914.1	0.065	0	0	0	0	0.047	0	0.574	0.587
XP_008467118.1	XP_008438969.1	537199	518799	3656.XP_008467118.1	3656.XP_008438969.1	0	0	0	0	0.052	0.281	0	0.423	0.572

XP_008443206.1	XP_008441351.1	521518	520304	3656.XP_008443206.1	3656.XP_008441351.1	0	0	0	0	0	0	0	0.563	0.563
XP_008444380.1	XP_008443553.1	522291	521754	3656.XP_008444380.1	3656.XP_008443553.1	0	0	0	0	0	0	0	0.563	0.563
XP_008452100.1	XP_008441351.1	527257	520304	3656.XP_008452100.1	3656.XP_008441351.1	0	0	0	0	0	0	0	0.562	0.562
XP_008444380.1	XP_008443206.1	522291	521518	3656.XP_008444380.1	3656.XP_008443206.1	0	0	0.313	0	0	0	0	0.36	0.541
XP_008463167.1	XP_008444380.1	534543	522291	3656.XP_008463167.1	3656.XP_008444380.1	0	0	0	0	0	0	0	0.536	0.536
XP_008445911.1	XP_008444380.1	532919	522291	3656.XP_008460758.1	3656.XP_008444380.1	0	0	0	0	0	0	0	0.522	0.522
XP_008440310.1	XP_008437427.1	519647	517790	3656.XP_008440310.1	3656.XP_008437427.1	0	0	0	0	0	0	0	0.504	0.504
XP_008449737.1	XP_008443206.1	525751	521518	3656.XP_008449737.1	3656.XP_008443206.1	0.174	0	0.363	0	0	0	0	0.095	0.482
XP_008452914.1	XP_008445911.1	527786	523289	3656.XP_008452914.1	3656.XP_008445911.1	0	0	0	0	0	0	0	0.455	0.455
XP_008463167.1	XP_008445911.1	534543	523289	3656.XP_008463167.1	3656.XP_008445911.1	0	0	0	0	0	0	0	0.453	0.453
XP_008465204.1	XP_008459427.1	535837	532081	3656.XP_008465204.1	3656.XP_008459427.1	0	0	0	0	0.302	0	0	0.206	0.422
XP_008467118.1	XP_008466126.1	537199	536479	3656.XP_008467118.1	3656.XP_008466126.1	0	0	0	0	0	0	0	0.423	0.422
XP_008459427.1	XP_008437557.1	532081	517882	3656.XP_008459427.1	3656.XP_008437557.1	0	0	0	0	0.302	0	0	0.206	0.422
XP_008453253.1	XP_008438969.1	528014	518799	3656.XP_008453253.1	3656.XP_008438969.1	0	0	0	0	0	0	0	0.422	0.422
XP_008457154.1	XP_008452100.1	530584	527257	3656.XP_008457154.1	3656.XP_008452100.1	0	0	0	0	0	0	0	0.423	0.422
XP_008452914.1	XP_008452100.1	527786	527257	3656.XP_008452914.1	3656.XP_008452100.1	0	0	0	0	0	0	0	0.42	0.42
XP_008465523.1	XP_008443206.1	536060	521518	3656.XP_008465523.1	3656.XP_008443206.1	0	0	0	0	0	0	0	0.421	0.42
XP_008443206.1	XP_008440310.1	521518	519647	3656.XP_008443206.1	3656.XP_008440310.1	0	0	0	0	0	0	0	0.42	0.42
XP_008446196.1	XP_008438007.1	523487	518180	3656.XP_008446196.1	3656.XP_008438007.1	0	0	0.444	0.581	0.056	0	0	0.624	0.42
XP_008444380.1	XP_008440310.1	522291	519647	3656.XP_008444380.1	3656.XP_008440310.1	0	0	0	0	0	0	0	0.42	0.42
XP_008460902.1	XP_008438007.1	533022	518180	3656.XP_008460902.1	3656.XP_008438007.1	0	0	0	0	0	0	0	0.42	0.42
XP_008460901.1	XP_008438007.1	533021	518180	3656.XP_008460901.1	3656.XP_008438007.1	0	0	0	0	0	0	0	0.42	0.42
XP_008459427.1	XP_008438007.1	532081	518180	3656.XP_008459427.1	3656.XP_008438007.1	0	0	0	0	0	0	0	0.42	0.42

XP_008453<br>253.1	XP_008452<br>100.1	528014	527257	3656.XP_0<br>08453253.1	3656.XP_0<br>08452100.1	0	0	0	0	0	0	0	0.421	0.42
XP_008452<br>100.1	XP_008438<br>969.1	527257	518799	3656.XP_0<br>08452100.1	3656.XP_0<br>08438969.1	0.085	0	0	0	0	0	0	0.378	0.406
XP_008467<br>118<br>.1	XP_008452<br>914	537199	527786<br><br>.1	3656.XP_0<br>08467118.	3656.XP_0<br>08452914.	0.171	0<br><br>1	0	0	0	0<br><br>1	0	0.312	0.405
XP_008467<br>118.1	XP_008441<br>351.1	537199	520304	3656.XP_0<br>08467118.1	3656.XP_0<br>08441351.1	0	0	0	0	0.101	0	0	0.363	0.402

Table S11 Subnetwork profile of full-ripe fruit (40 DAP) generated by STRING and Cytoscape analyses.

ID name	BetweennessCentrality	ClosenessCentrality	Degree	NumberOfDirectedEdges
XP_008438929.1	0.84210526	0.52777778	9	9
XP_008447733.1	0.28070175	0.32758621	4	4
XP_008452849.1	0	0.36538462	3	3
XP_008450968.1	0	0.25675676	3	3
XP_008455290.1	0	0.25675676	3	3
XP_008448661.1	0	0.25675676	3	3
XP_008438779.1	0	0.36538462	3	3
XP_008446732.1	0.28070175	0.40425532	3	3
XP_008465290.1	0	0.36538462	3	3
XP_008460595.1	0.10526316	0.36538462	2	2
XP_008459496.1	0	0.38	2	2
XP_008451613.1	0.35087719	0.41304348	2	2
XP_008444821.1	0.19883041	0.31147541	2	2
XP_008463923.1	0.19883041	0.38	2	2
XP_008466011.1	0.10526316	0.28787879	2	2
XP_008448578.1	0.10526316	0.24675325	2	2
XP_008443958.1	0	1	1	1
XP_008444036.1	0	1	1	1
XP_008443230.1	0	0.35185185	1	1
XP_008451866.1	0	0.2	1	1
XP_008461049.1	0	0.27142857	1	1
XP_008460254.1	0	0.22619048	1	1

Table S12 STRING interactions of subnetwork full-ripe fruit (40 DAP).

#node1	node2	node1_string_internal_id	node2_string_internal_id	node1_external_id	node2_external_id	neighborhood_on_chromosome	gene_fusion	phylogenetic_cooccurrence	homology	coexpression	experimentally_determined_interaction	database_annotated	automated_textmining	combined_score
XP_008465290.1	XP_008452849.1	535896	527746	3656.XP_008465290.1	3656.XP_008452849.1	0.127	0	0	0	0.384	0.361	0	0.692	0.879
XP_008448578.1	XP_008444821.1	524999	522606	3656.XP_008448578.1	3656.XP_008444821.1	0	0	0	0	0.075	0.825	0	0.069	0.836
XP_008451866.1	XP_008448578.1	527092	524999	3656.XP_008451866.1	3656.XP_008448578.1	0	0	0	0	0	0	0	0.711	0.711
XP_008461049.1	XP_008460595.1	533123	532800	3656.XP_008461049.1	3656.XP_008460595.1	0	0	0	0.595	0	0.193	0.6	0.221	0.688
XP_008450968.1	XP_008448661.1	526528	525048	3656.XP_008450968.1	3656.XP_008448661.1	0.055	0	0	0	0.072	0.045	0.65	0.08	0.681
XP_008455290.1	XP_008450968.1	529346	526528	3656.XP_008455290.1	3656.XP_008450968.1	0.055	0	0	0	0.072	0.045	0.65	0.08	0.681
XP_008452849.1	XP_008438779.1	527746	518690	3656.XP_008452849.1	3656.XP_008438779.1	0	0	0	0	0.359	0.288	0	0.336	0.67
XP_008455290.1	XP_008448661.1	529346	525048	3656.XP_008455290.1	3656.XP_008448661.1	0	0	0.449	0.971	0	0	0.65	0.22	0.656
XP_008463923.1	XP_008438929.1	535028	518778	3656.XP_008463923.1	3656.XP_008438929.1	0	0	0	0	0	0	0	0.628	0.628
XP_008450968.1	XP_008447733.1	526528	524493	3656.XP_008450968.1	3656.XP_008447733.1	0	0	0	0	0	0	0.6	0	0.6
XP_008455290.1	XP_008447733.1	529346	524493	3656.XP_008455290.1	3656.XP_008447733.1	0	0	0	0	0	0	0.6	0	0.6
XP_008448661.1	XP_008447733.1	525048	524493	3656.XP_008448661.1	3656.XP_008447733.1	0	0	0	0	0	0	0.6	0	0.6
XP_008451613.1	XP_008438929.1	526931	518778	3656.XP_008451613.1	3656.XP_008438929.1	0	0	0	0	0	0	0	0.562	0.562
XP_008446732.1	XP_008444821.1	523841	522606	3656.XP_008446732.1	3656.XP_008444821.1	0	0	0	0	0.125	0.154	0	0.42	0.533
XP_008444036.1	XP_008443958.1	522087	522029	3656.XP_008444036.1	3656.XP_008443958.1	0	0	0	0	0	0	0	0.533	0.533
XP_008460595.1	XP_008438929.1	532800	518778	3656.XP_008460595.1	3656.XP_008438929.1	0	0	0	0	0	0	0	0.523	0.523
XP_008466011.1	XP_008463923.1	536414	535028	3656.XP_008466011.1	3656.XP_008463923.1	0	0	0	0	0	0.089	0	0.495	0.52
XP_008446732.1	XP_008438929.1	523841	518778	3656.XP_008446732.1	3656.XP_008438929.1	0	0	0	0	0.045	0	0	0.502	0.504
XP_008451613.1	XP_008447733.1	526931	524493	3656.XP_008451613.1	3656.XP_008447733.1	0	0	0	0	0	0	0	0.486	0.485

XP_008465290.1	XP_008438779.1	535896	518690	3656.XP_008465290.1	3656.XP_008438779.1	0	0	0	0	0.187	0.343	0	0.096	0.474
XP_008438929.1	XP_008438779.1	518778	518690	3656.XP_008438929.1	3656.XP_008438779.1	0	0	0	0	0.046	0.091	0	0.439	0.471
XP_008465290.1	XP_008438929.1	535896	518778	3656.XP_008465290.1	3656.XP_008438929.1	0	0	0	0	0.045	0.182	0	0.356	0.452
XP_008466011.1	XP_008460254.1	536414	532610	3656.XP_008466011.1	3656.XP_008460254.1	0	0	0	0	0	0	0	0.432	0.432
XP_008459496.1	XP_008446732.1	532127	523841	3656.XP_008459496.1	3656.XP_008446732.1	0	0	0	0	0	0	0	0.422	0.422
XP_008459496.1	XP_008438929.1	532127	518778	3656.XP_008459496.1	3656.XP_008438929.1	0	0	0	0	0	0	0	0.421	0.42
XP_008452849.1	XP_008438929.1	527746	518778	3656.XP_008452849.1	3656.XP_008438929.1	0	0	0	0	0	0	0	0.42	0.42
XP_008443230.1	XP_008438929.1	521530	518778	3656.XP_008443230.1	3656.XP_008438929.1	0	0	0	0	0	0.302	0	0.188	0.408
