# Supplementary material for: Transcriptome profiling of non-climacteric ‘yellow’ melon during ripening: insights on sugar metabolism
Source: BMC Genomics. 2020 Mar 30;21:262. doi: 10.1186/s12864-020-6667-0 (PMC7106763; doi:10.1186/s12864-020-6667-0)
Supplement: Supplementary file 10 — Additional File 10: Figures S7, S8, S9. KEGG (Kyoto Encyclopedia of Genes and Genomes) analyses using Pathview software (https://pathview.uncc.edu/) of “starch and sucrose metabolism”, “galactose metabolism” and “amino sugar and nucleotide sugar metabolism”. The colour bar represents de log2 fold change of the maturation process and ranges from green (up-regulated genes in 40 DAP fruit) to red (up-regulated genes in 10 DAP fruit). The blue letters are enzyme short names described in the KEGG pathway and the purple are enzyme short names that were described in the literature associated with the sugar pathway [6, 9]. There are protein isoforms that act in the same metabolic route and the information of all log2 fold change were included. [file 12864_2020_6667_MOESM10_ESM.pdf]

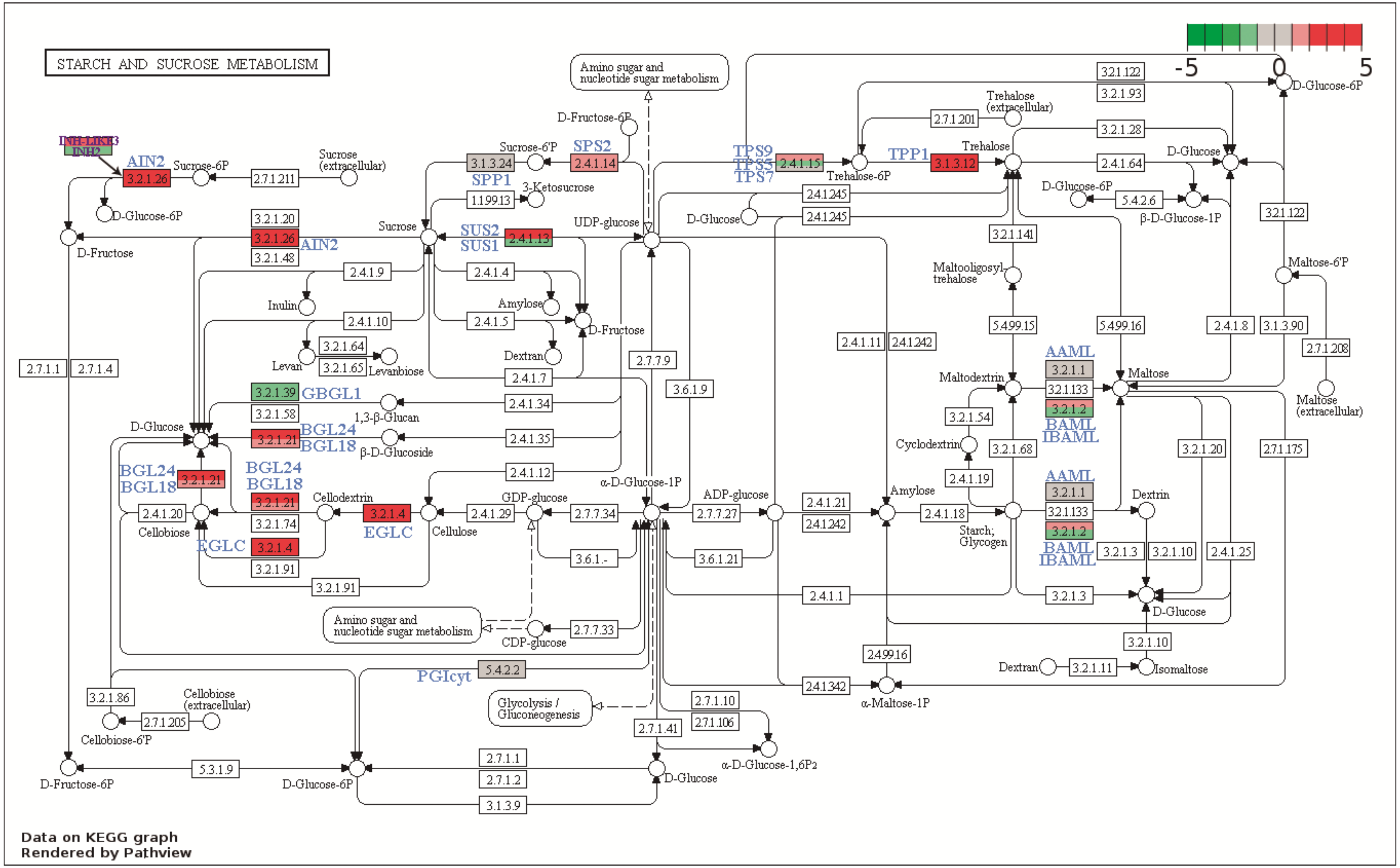

Figure S7 “Starch and sucrose metabolism” (cm00500). The color bar represents de log2 FoldChange of maturation process and ranges from green (up-regulated genes in 40 DAP fruit) to red (up-regulated genes in 10 DAP fruit). The blues letters are enzymes short names described in KEEG pathway and the purple are enzymes short names that were described in the literature associated with sugar pathway [13].

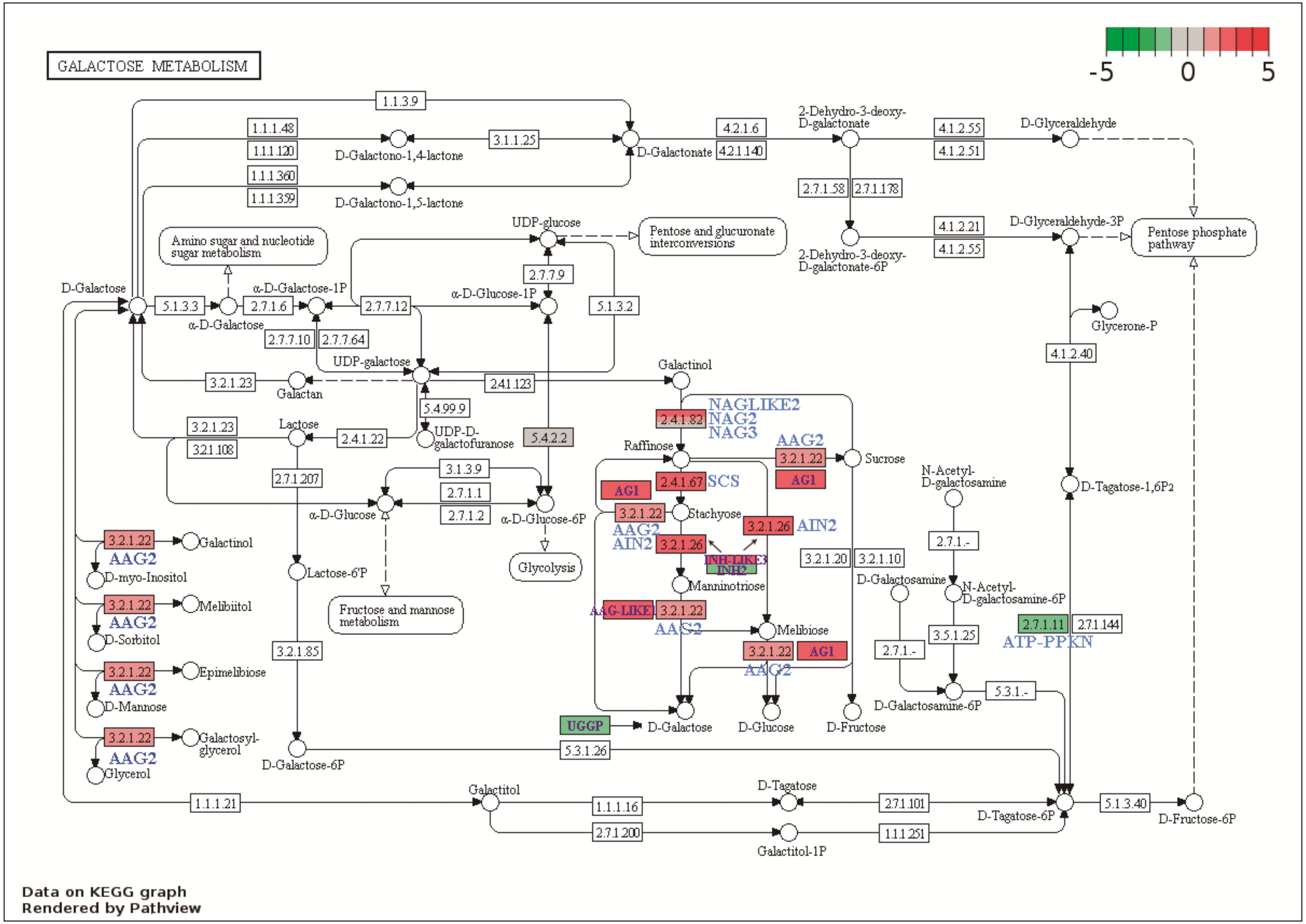

Figure S8 "Galactose metabolism" (cm00052). The color bar represents de log2 FoldChange of maturation process and ranges from green (up-regulated genes in 40 DAP fruit) to red (up-regulated genes in 10 DAP fruit). The blues letters are enzymes short names described in KEEG pathway and the purple are enzymes short names that were described in the literature associated with sugar pathway [13].

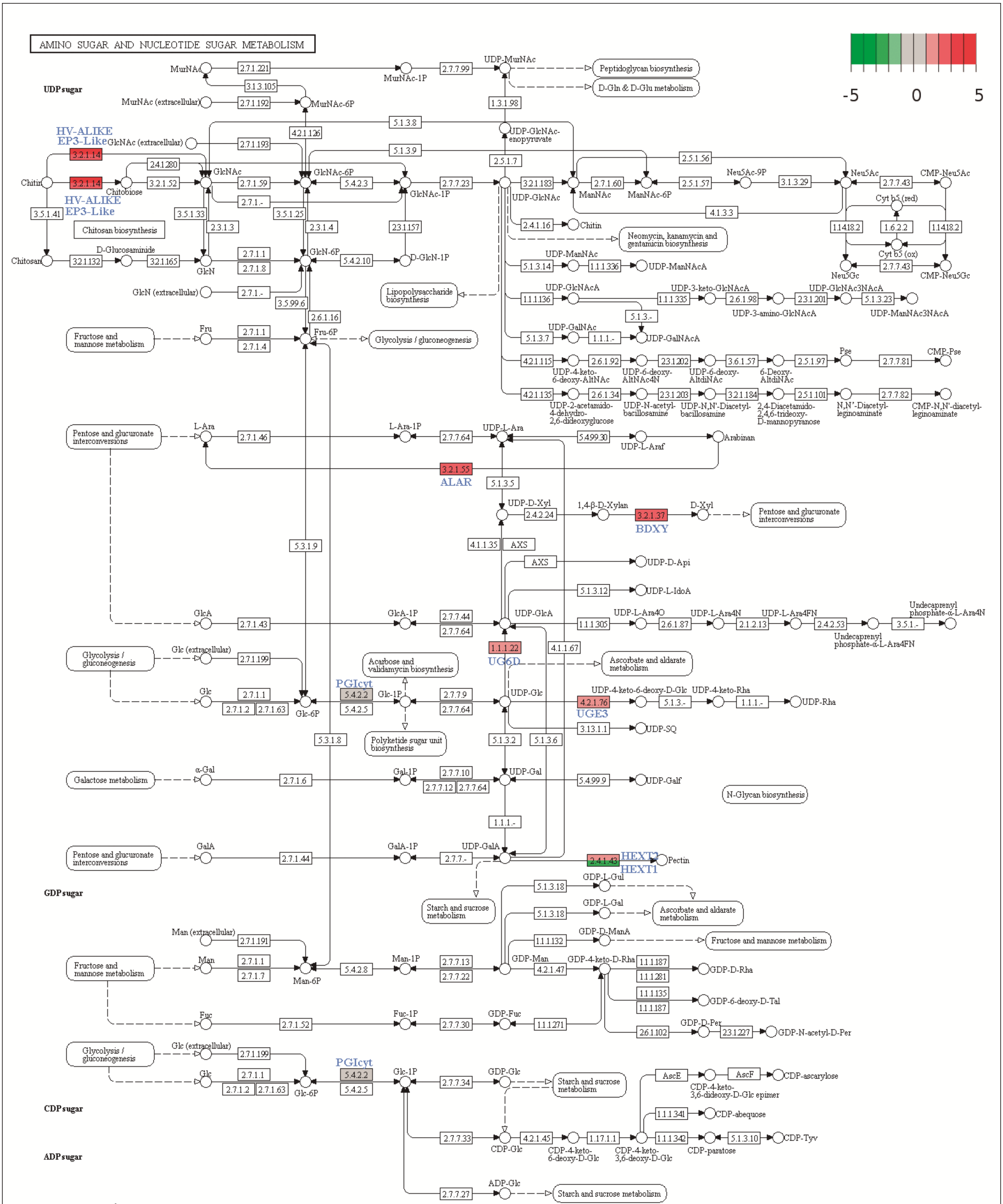

Data on KEGG graph
Rendered by Pathview

Figure S9 “Amino sugar and nucleotide sugar metabolism” (cm00520). The color bar represents de log2 FoldChange of maturation process and ranges from green (up-regulated genes in 40 DAP fruit) to red (up-regulated genes in 10 DAP fruit). The blues letters are enzymes short names described in KEEG pathway and the purple are enzymes short names that were described in the literature associated with sugar pathway [13].
