# Supplementary material for: Transcriptome profiling of non-climacteric ‘yellow’ melon during ripening: insights on sugar metabolism
Source: BMC Genomics. 2020 Mar 30;21:262. doi: 10.1186/s12864-020-6667-0 (PMC7106763; doi:10.1186/s12864-020-6667-0)

Figure S10: Graphics of the normalized counts reads per gene obtained by RNA-seq results (plotCounts function of DESeq2 analysis - differential gene expression analysis based on the negative binomial distribution).

1) DE genes not present in KEEG pathway database.

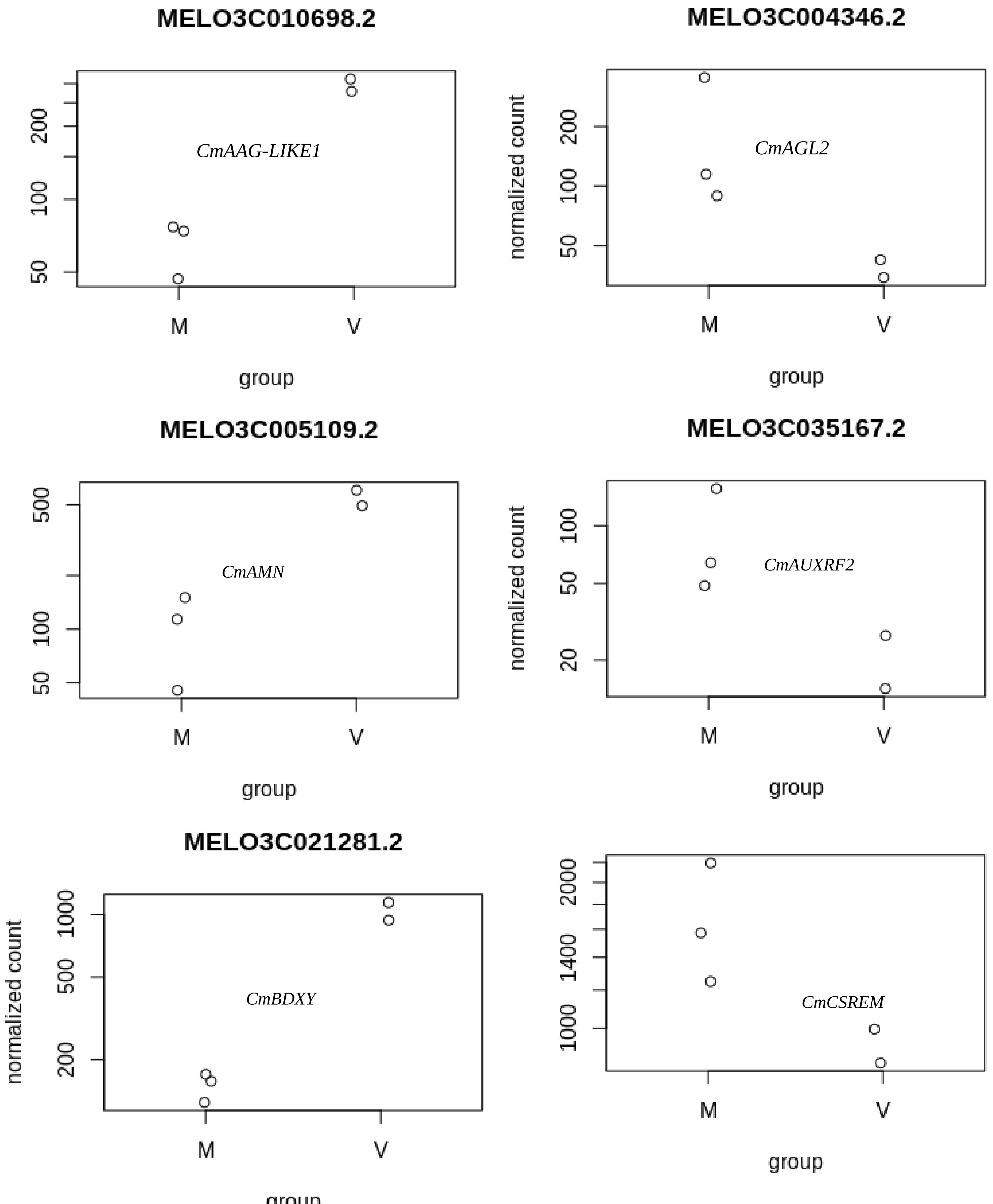

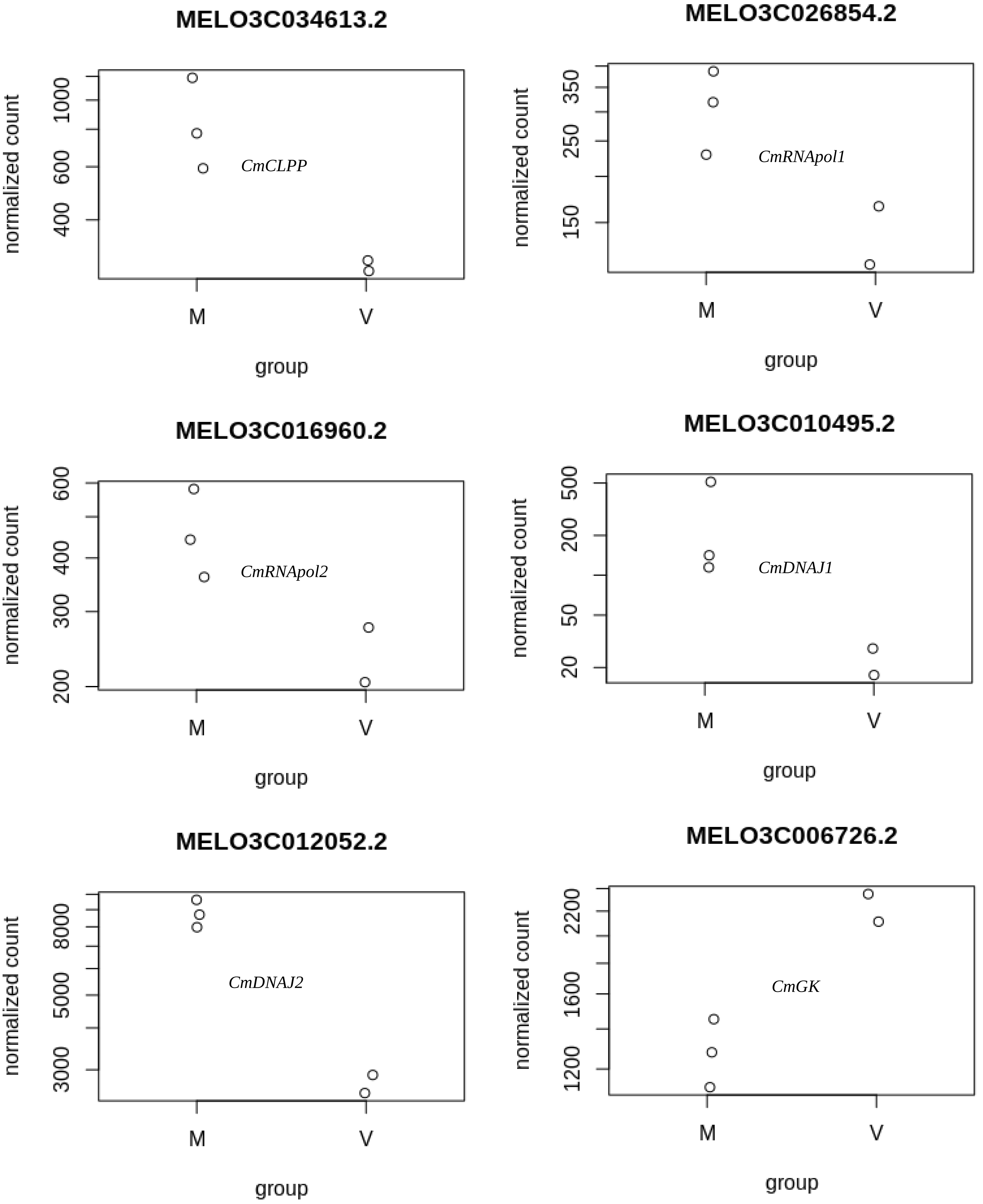
MELO3C034613.2
normalized count
400 600 1000
CmCLPP
M V
group
MELO3C026854.2
normalized count
150 250 350
CmRNApol1
M V
group
MELO3C016960.2
normalized count
200 300 400 600
CmRNApol2
M V
group
MELO3C010495.2
normalized count
20 50 200 500
CmDNAJ1
M V
group
MELO3C012052.2
normalized count
3000 5000 8000
CmDNAJ2
M V
group
MELO3C006726.2
normalized count
1200 1600 2200
CmGK
M V
group

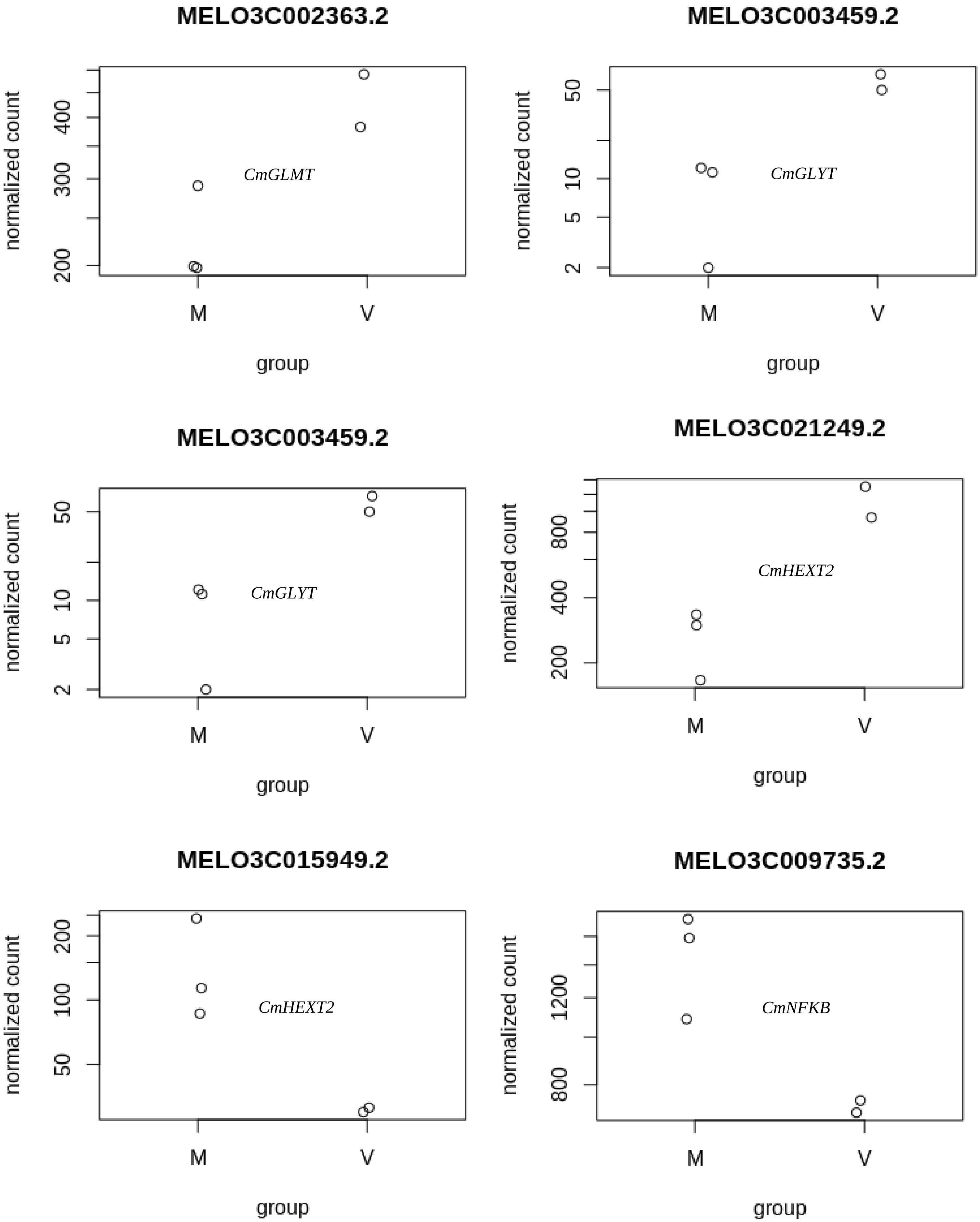
MELO3C002363.2
CmGLMT
normalized count
200
300
400
M
V
group
MELO3C003459.2
CmGLYT
normalized count
2
5
10
50
M
V
group
MELO3C003459.2
CmGLYT
normalized count
2
5
10
50
M
V
group
MELO3C021249.2
CmHEXT2
normalized count
200
400
800
M
V
group
MELO3C015949.2
CmHEXT2
normalized count
50
100
200
M
V
group
MELO3C009735.2
CmNFKB
normalized count
800
1200
M
V
group

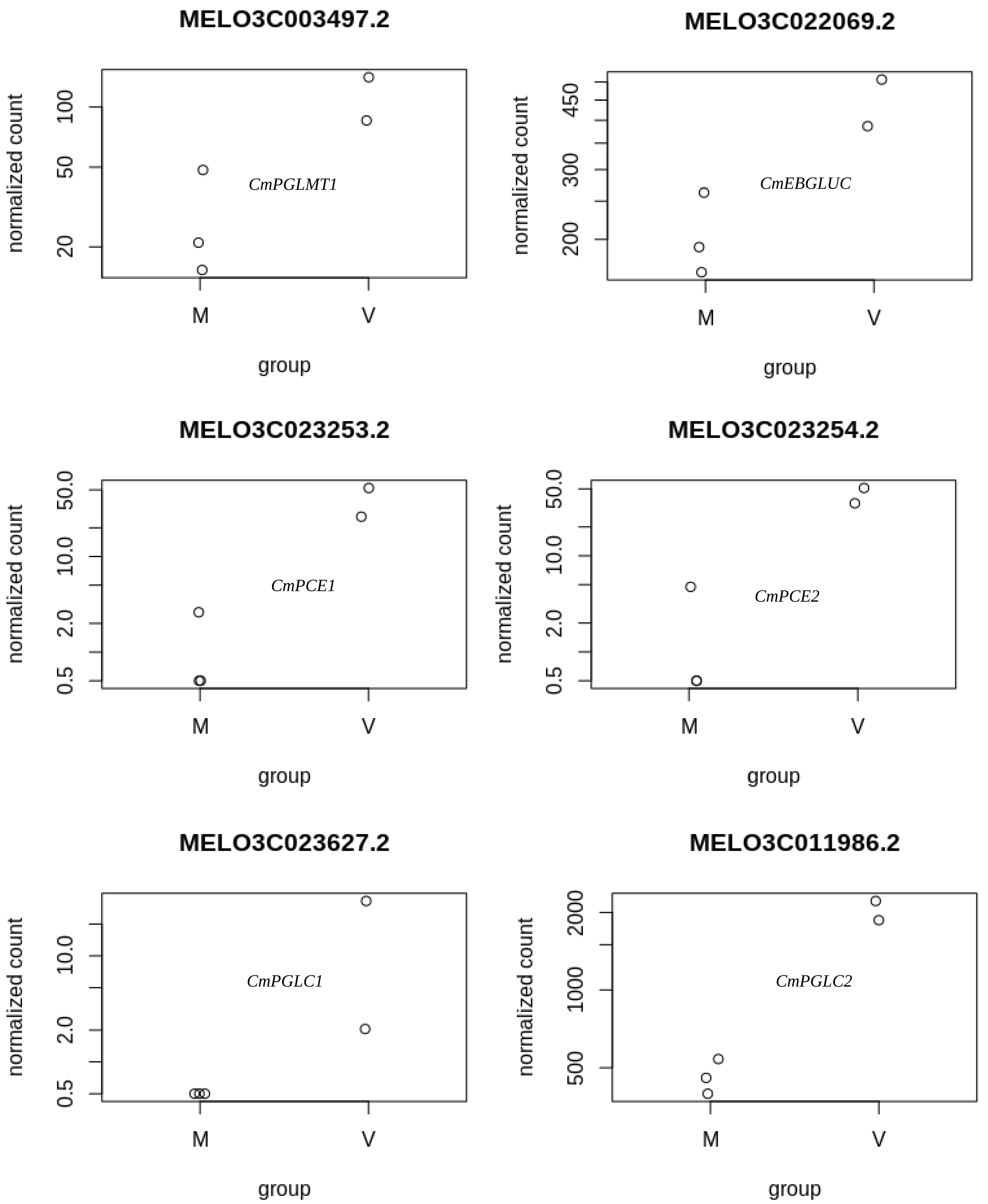
MELO3C003497.2
CmPGLMT1
normalized count
100
50
20
M
V
group
MELO3C022069.2
CmEBGLUC
normalized count
450
300
200
M
V
group
MELO3C023253.2
CmPCE1
normalized count
50.0
10.0
2.0
0.5
M
V
group
MELO3C023254.2
CmPCE2
normalized count
50.0
10.0
2.0
0.5
M
V
group
MELO3C023627.2
CmPGLC1
normalized count
10.0
2.0
0.5
M
V
group
MELO3C011986.2
CmPGLC2
normalized count
2000
1000
500
M
V
group

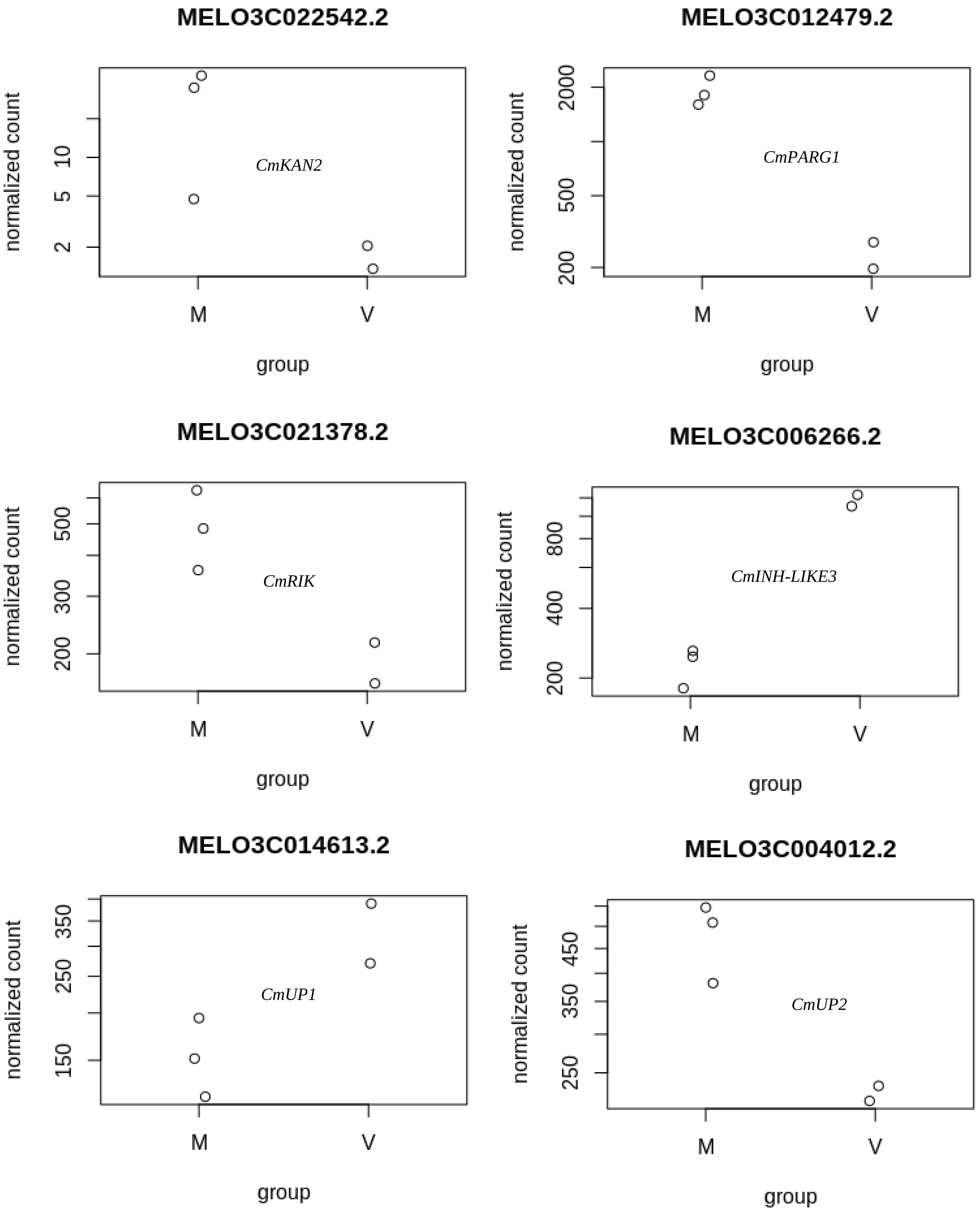
MELO3C022542.2
CmKAN2
normalized count
2
5
10
M
V
group
MELO3C012479.2
CmPARG1
normalized count
200
500
2000
M
V
group
MELO3C021378.2
CmRIK
normalized count
200
300
500
M
V
group
MELO3C006266.2
CmINH-LIKE3
normalized count
200
400
800
M
V
group
MELO3C014613.2
CmUP1
normalized count
150
250
350
M
V
group
MELO3C004012.2
CmUP2
normalized count
250
350
450
M
V
group

2) Pentose and glucuronate interconversions (cmo00040).

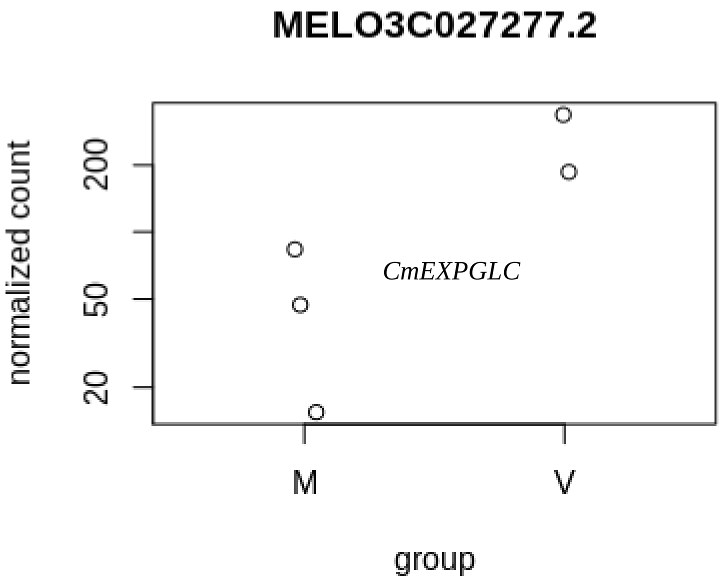

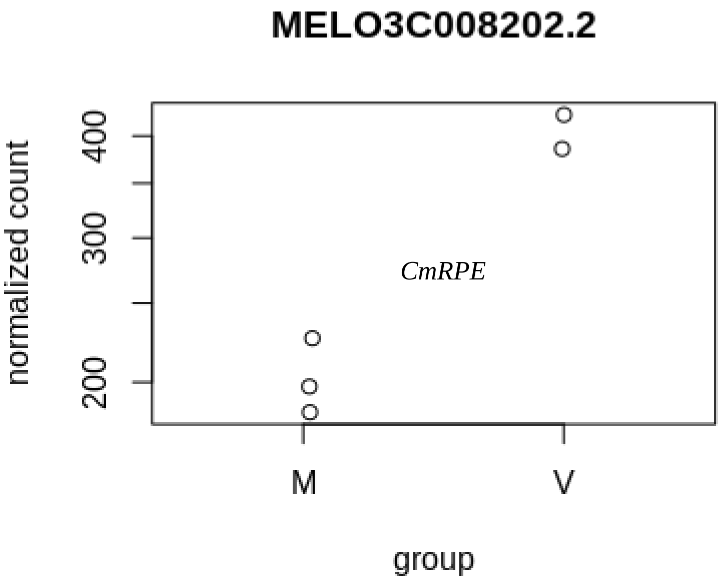

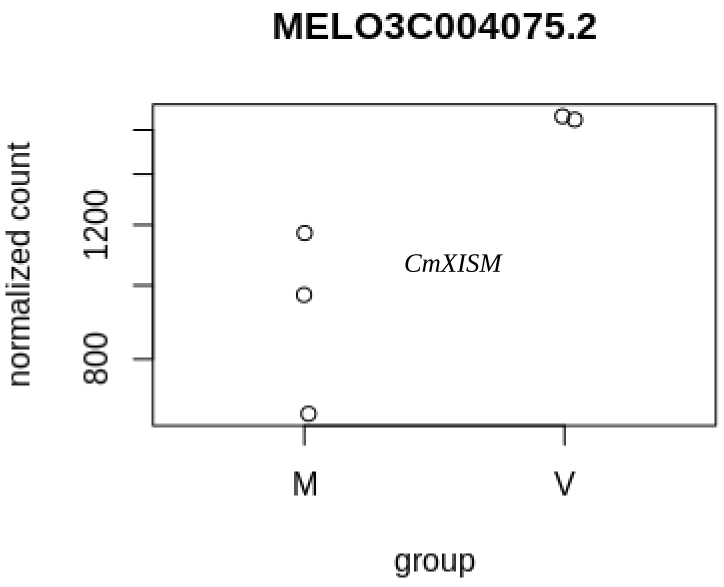

3) Pentose and glucuronate interconversions (cmo00040); galactose metabolismo (cmo00052) and amino sugar and nucleotide sugar metabolismo (cmo00520).

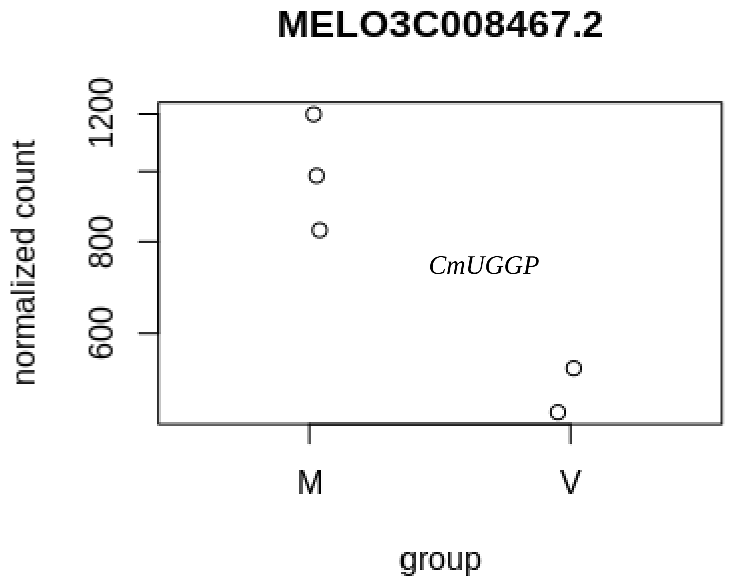

4) Pentose and glucuronate interconversions (cmo00040) and amino sugar and nucleotide sugar metabolismo (cmo00520).

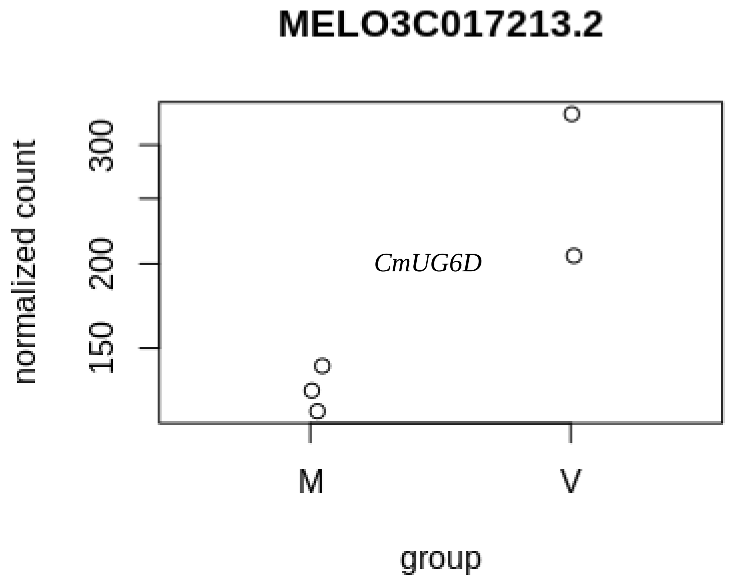

5) Galactose metabolism (cmo00052).

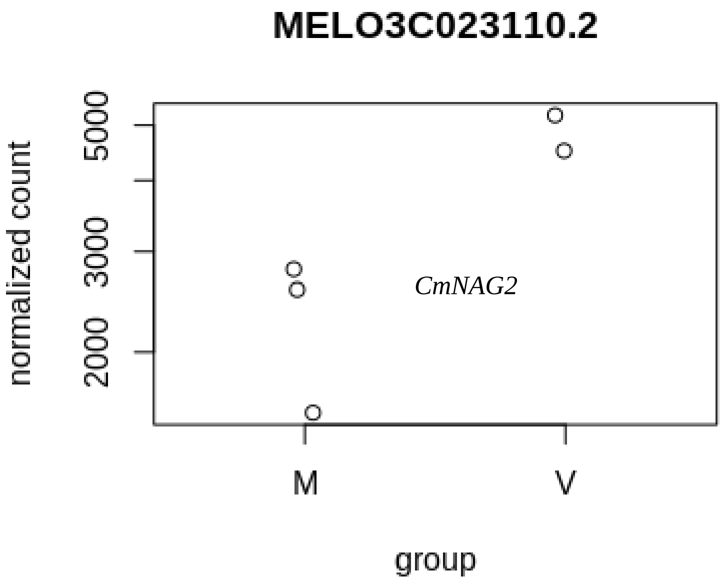

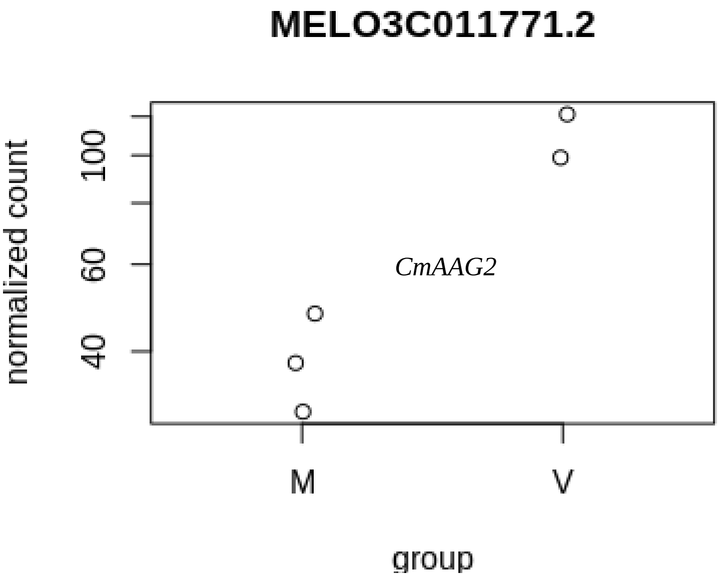

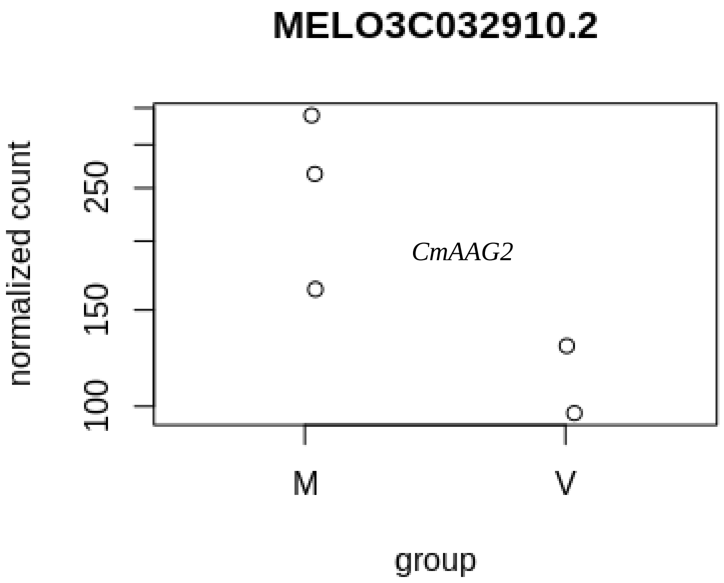

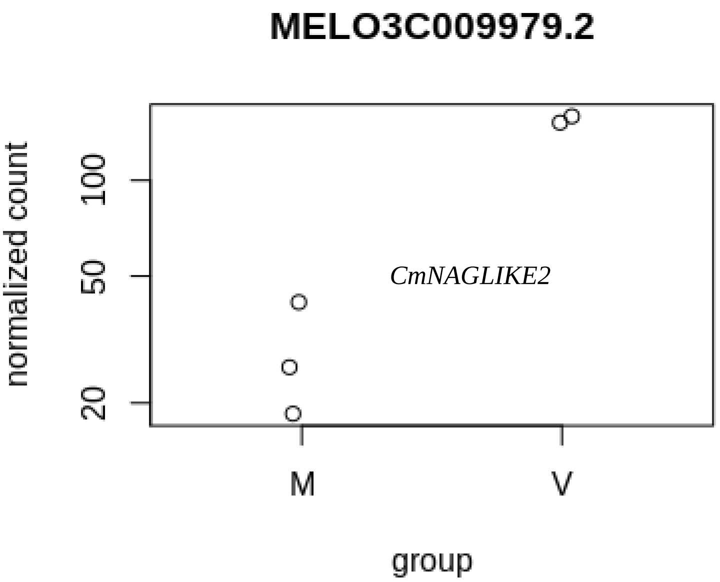

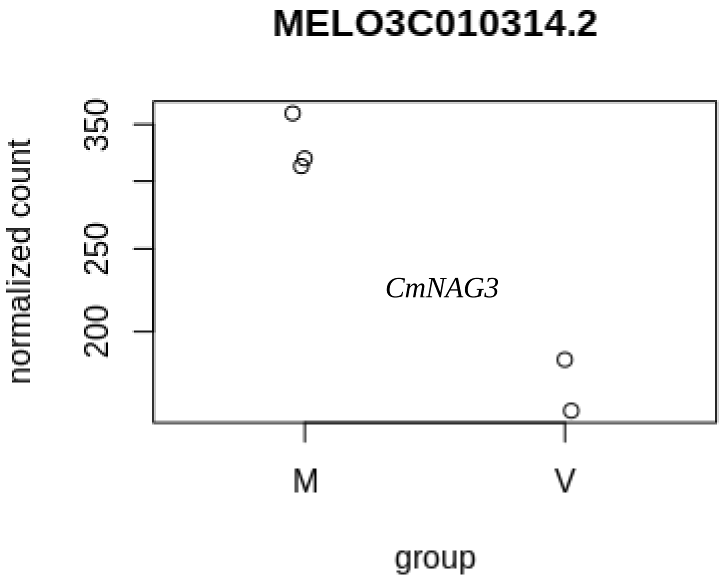

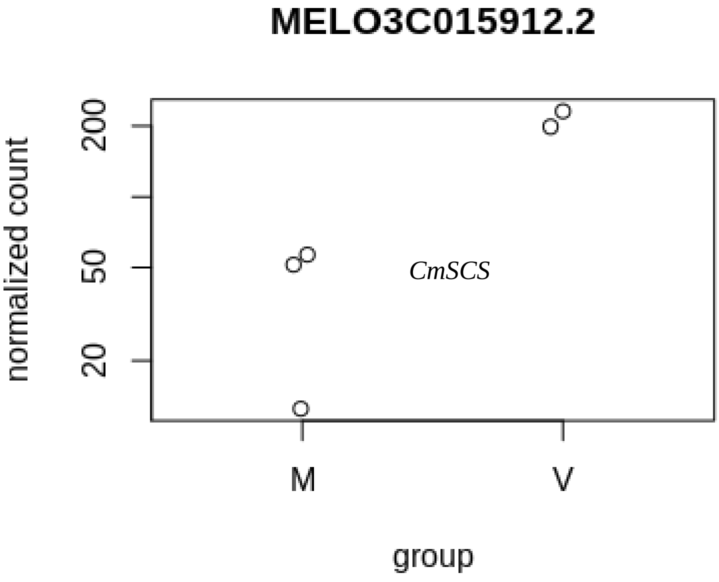

6) Galactose metabolism (cmo00052) and starch and sucrose metabolismo (cmo00500).

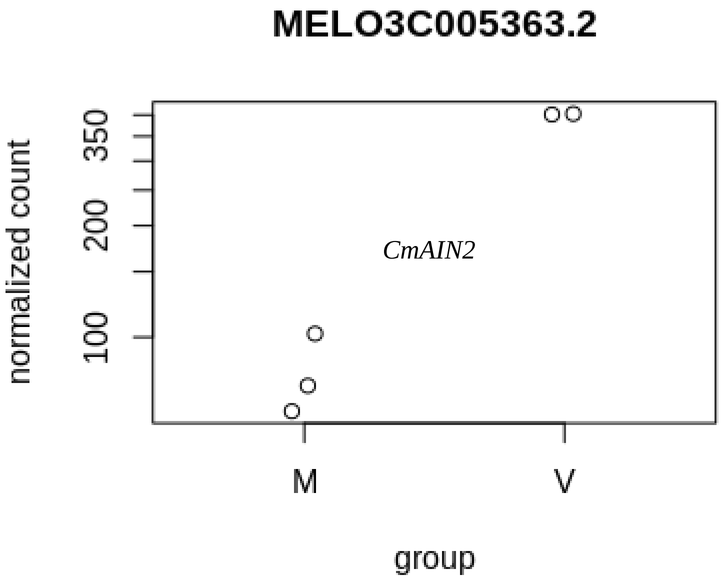

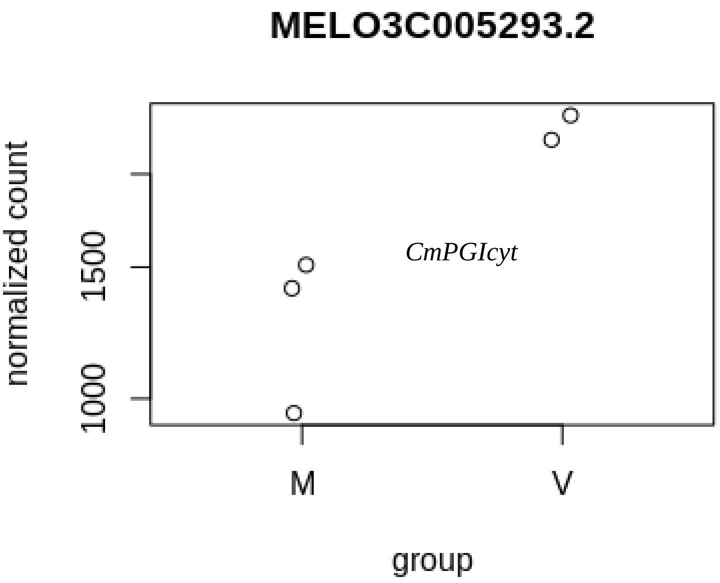

7) sucrose metabolism (cmo00500).

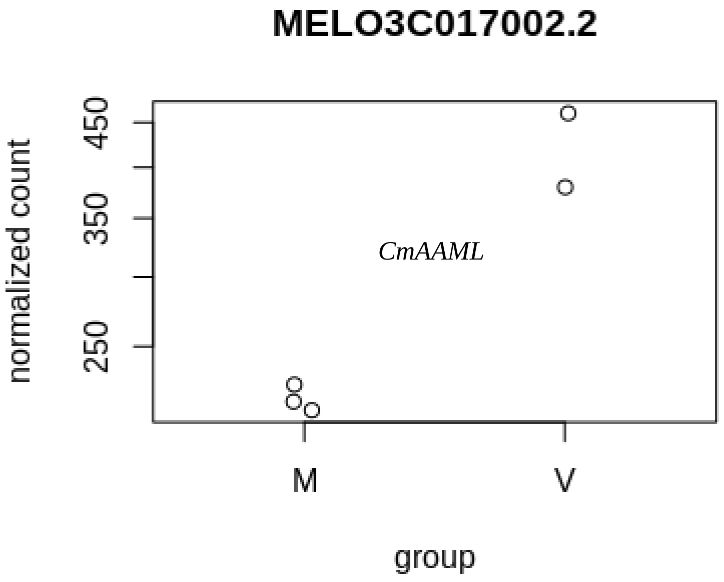

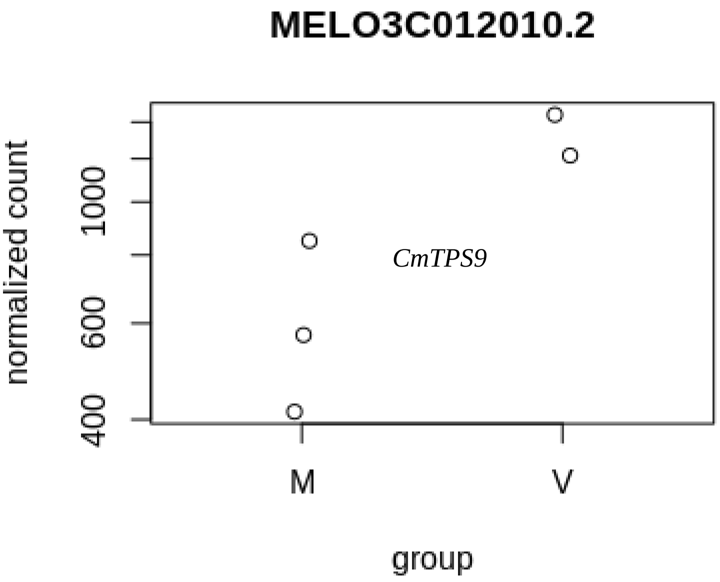

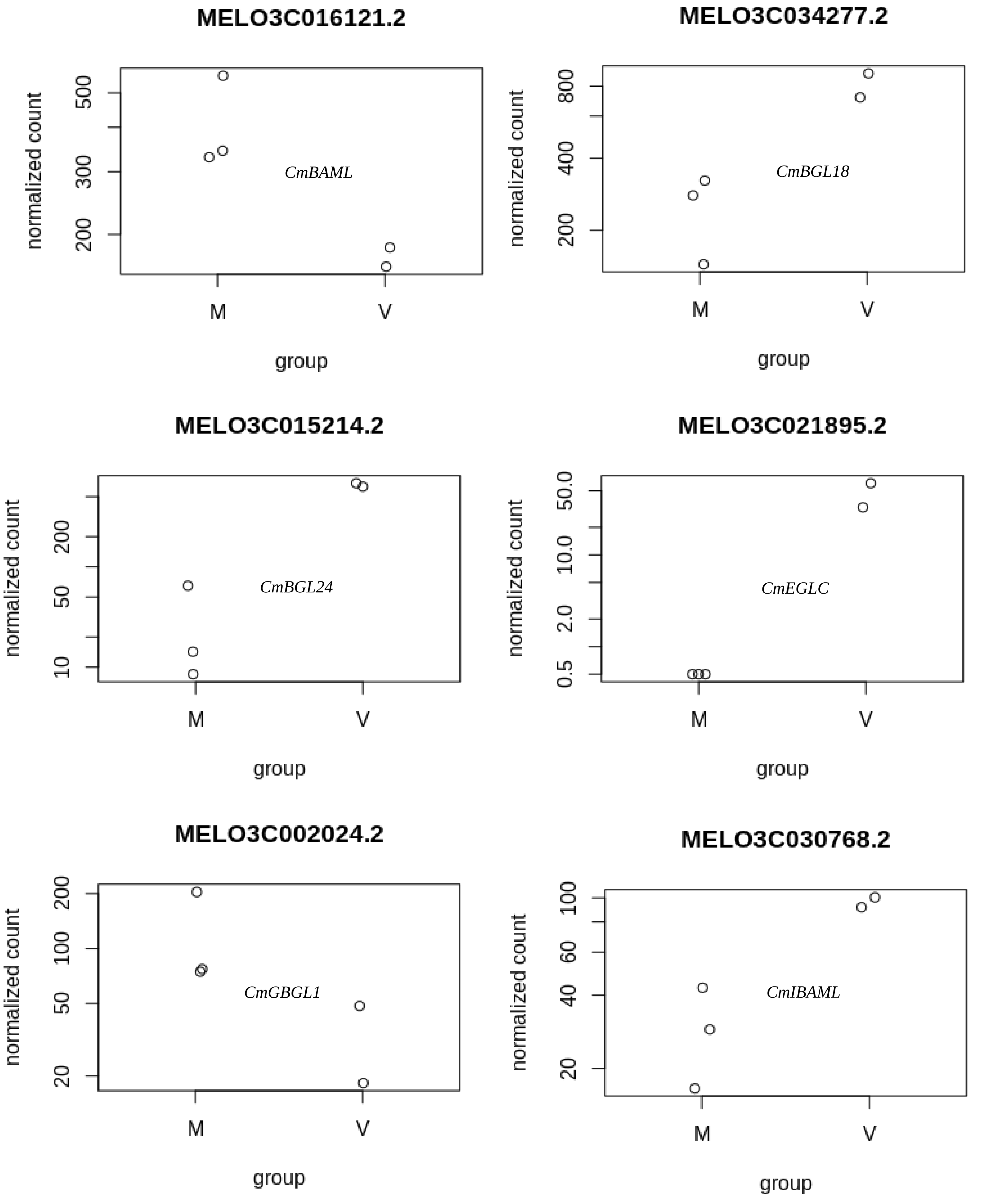
MELO3C016121.2
CmBAML
MELO3C034277.2
CmBGL18
MELO3C015214.2
CmBGL24
MELO3C021895.2
CmEGLC
MELO3C002024.2
CmGBGL1
MELO3C030768.2
CmIBAML
normalized count
group
M
V

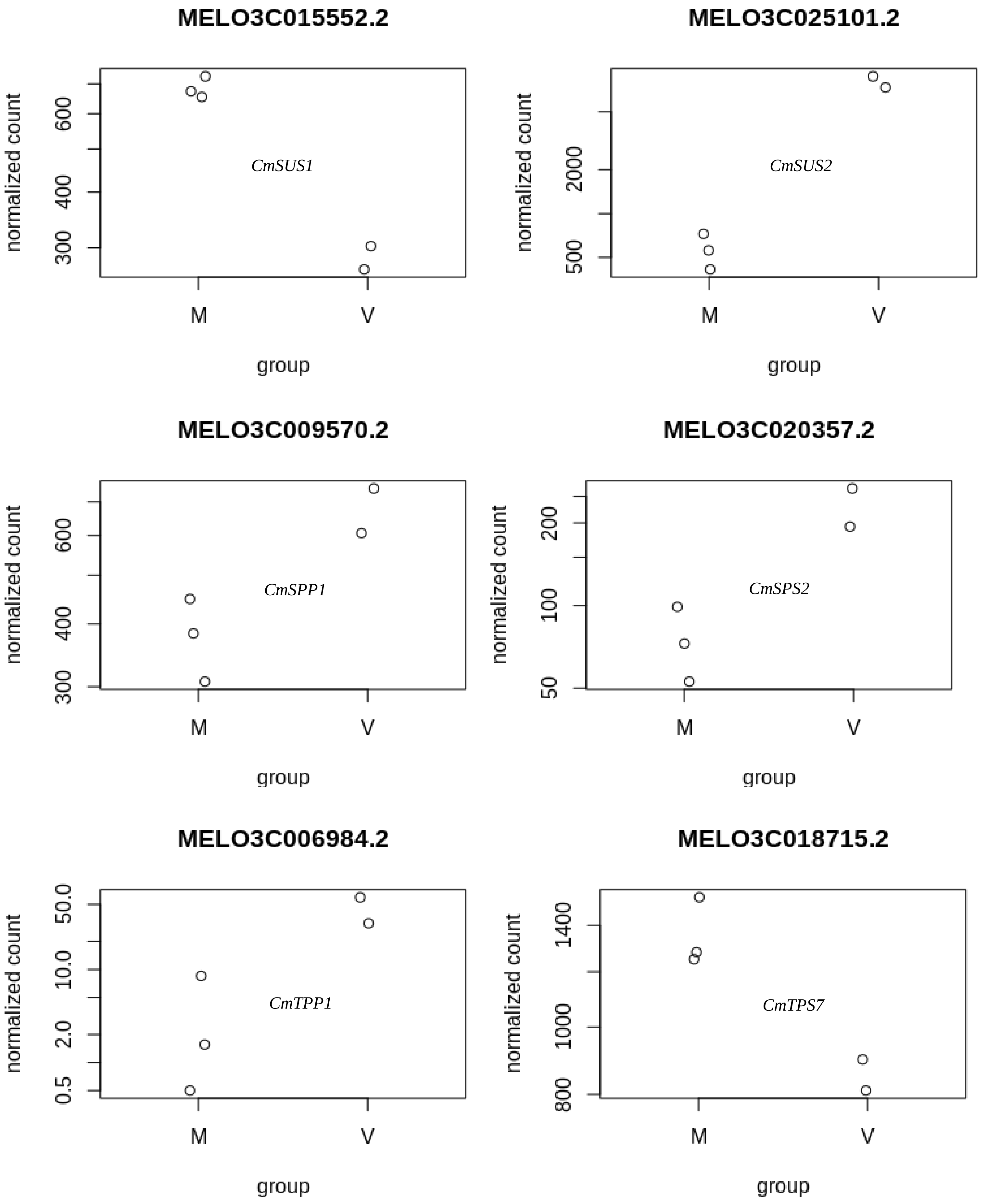
MELO3C015552.2
CmSUS1
normalized count
300
400
600
M
V
group
MELO3C025101.2
CmSUS2
normalized count
500
2000
M
V
group
MELO3C009570.2
CmSPP1
normalized count
300
400
600
M
V
group
MELO3C020357.2
CmSPS2
normalized count
50
100
200
M
V
group
MELO3C006984.2
CmTPP1
normalized count
0.5
2.0
10.0
50.0
M
V
group
MELO3C018715.2
CmTPS7
normalized count
800
1000
1400
M
V
group

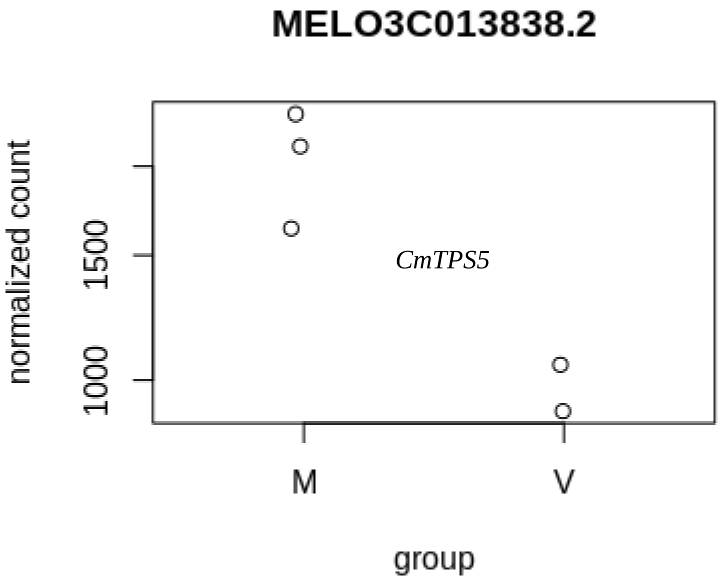

8) Amino sugar and nucleotide sugar metabolism (cmo00520).

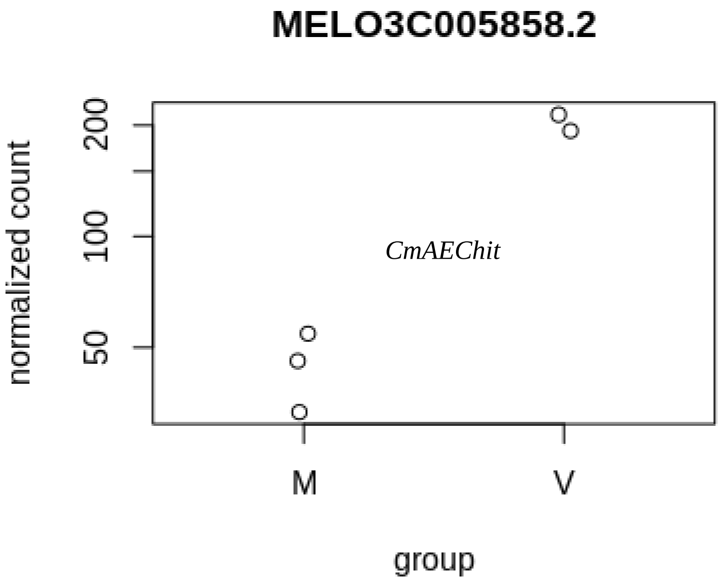

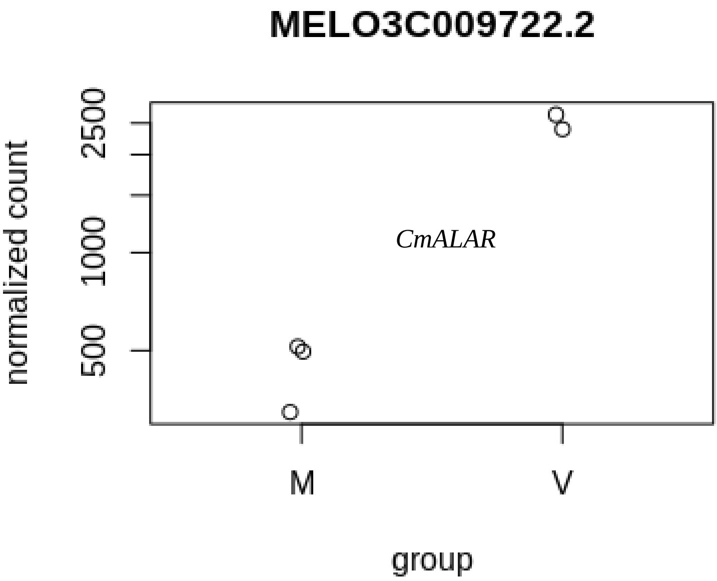

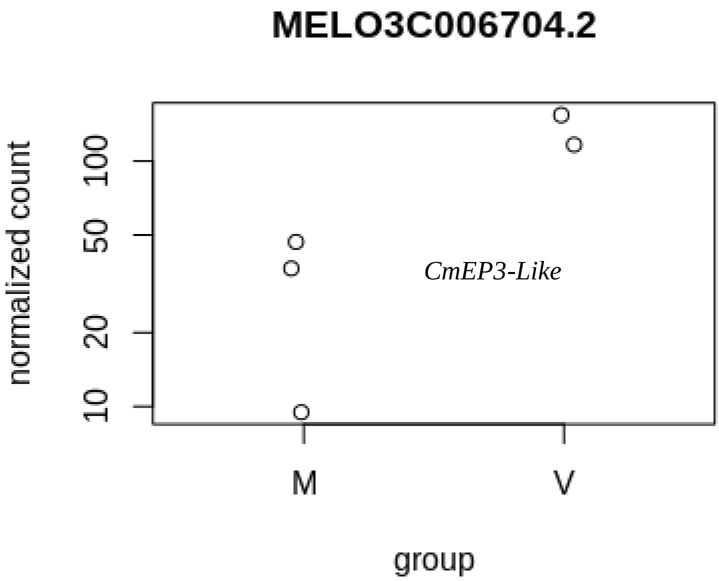

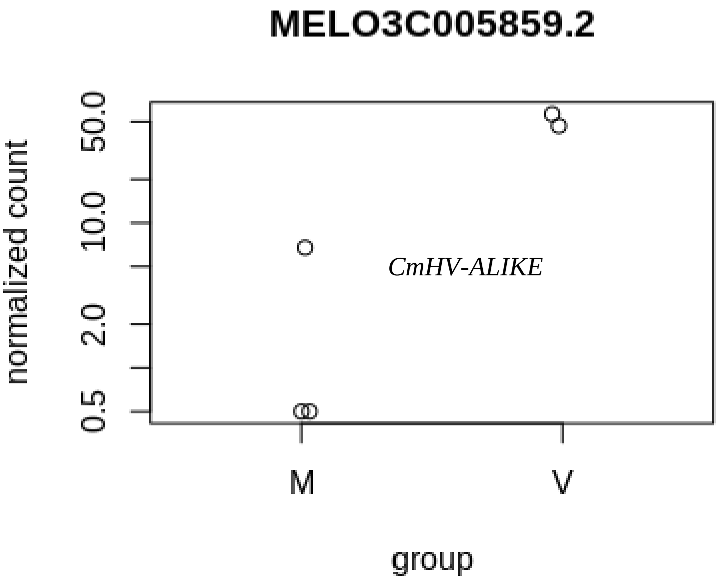

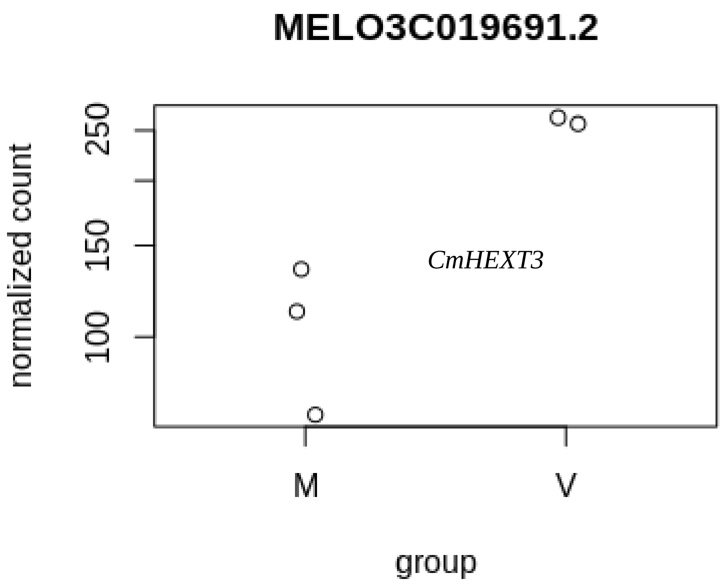

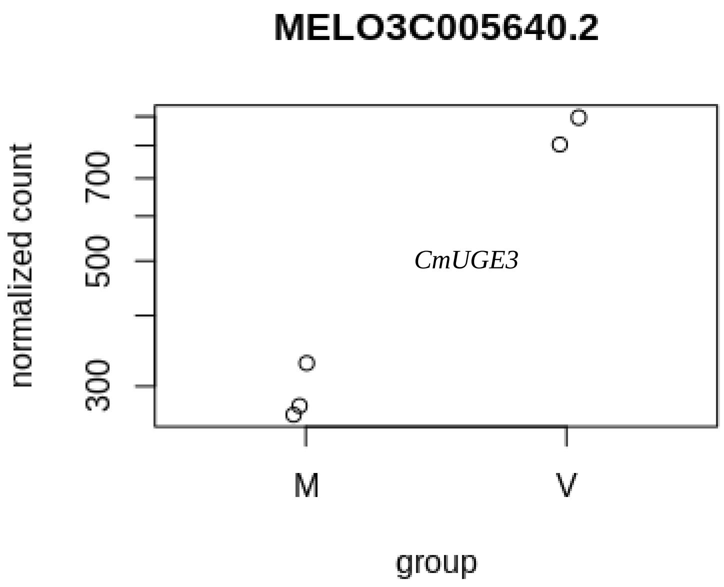

9) Plant hormone signal transduction (cmo04075).

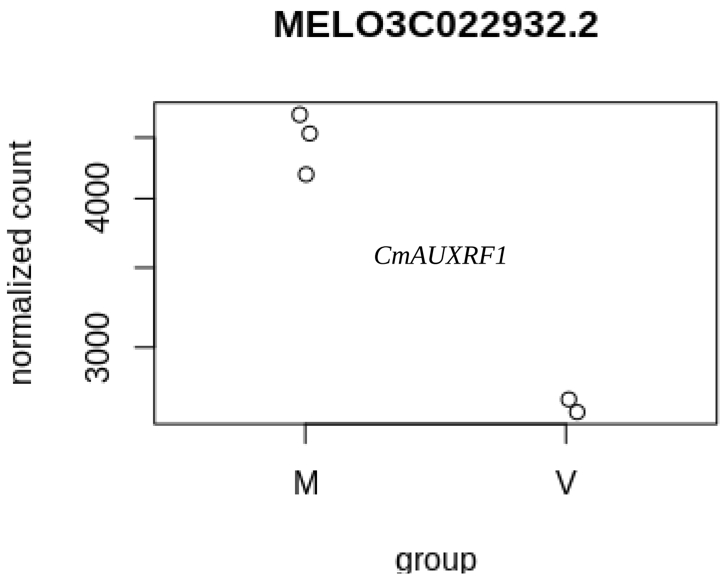

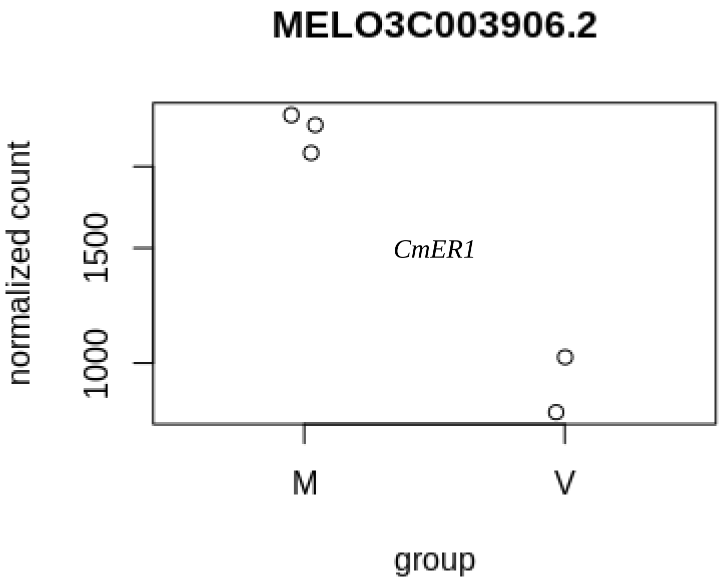

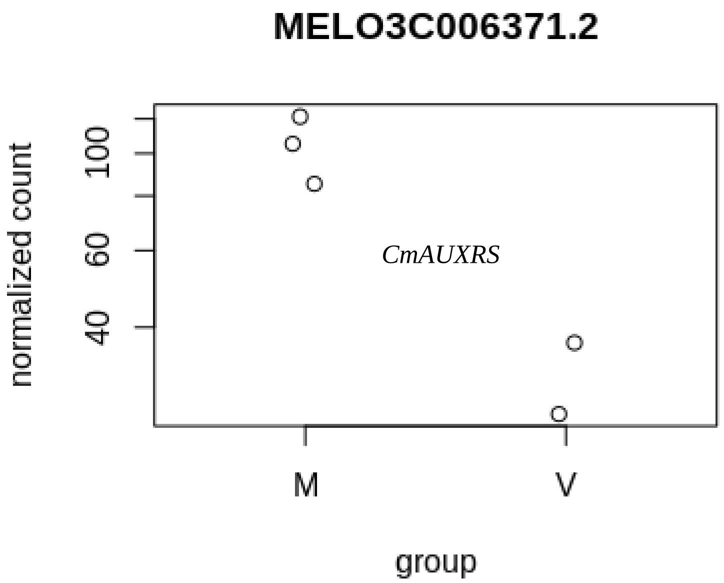

10) Protein processing in endoplasmic reticulum (cmo04141).

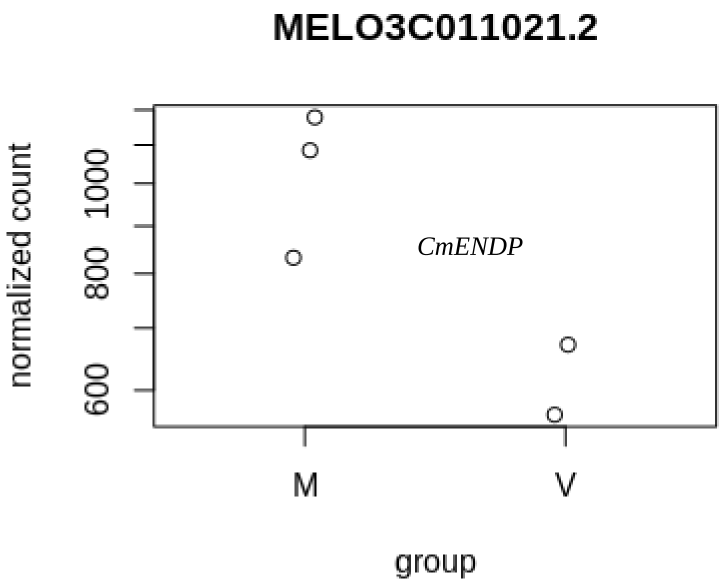

Supplement: Supplementary file 11 — Additional File 11: Figure S10. Graphics of the normalized gene counts obtained by RNA-seq results (plotCounts function of DESeq2 analysis – differential gene expression analysis based on negative binomial distribution). [file 12864_2020_6667_MOESM11_ESM.pdf]
