# Supplementary material for: Transcriptome profiling of non-climacteric ‘yellow’ melon during ripening: insights on sugar metabolism
Source: BMC Genomics. 2020 Mar 30;21:262. doi: 10.1186/s12864-020-6667-0 (PMC7106763; doi:10.1186/s12864-020-6667-0)
Supplement: Supplementary file 12 — Additional File 12: Figure S11. Alignment of protein isoforms related to sugar metabolism or associated pathways. The domains were detected by Pfam database (https://pfam.xfam.org/) and the amino acid sequences were aligned by the MUSCLE algorithm. [file 12864_2020_6667_MOESM12_ESM.pdf]

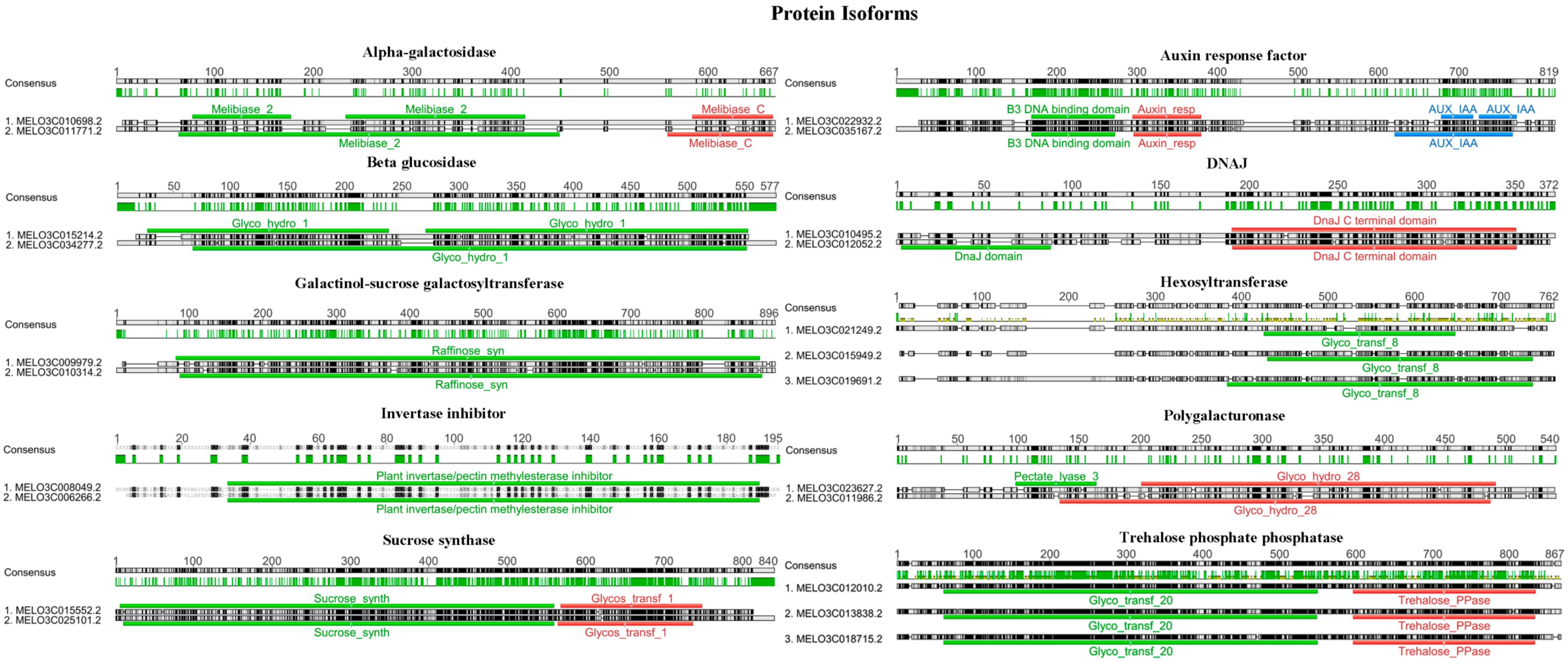

Figure S11 Alignment of proteins isoforms related with sugar metabolism or associated pathways. The domains were detected by Pfam database (https://pfam.xfam.org/) and the amino acid sequences were aligned by MUSCLE algorithm.

.

Protein isoform sequences alignment using MUSCLE algorithm and similarity percentage (http://imed.med.ucm.es/Tools/sias.html software). (*)as sequências idênticas na posição; (:) substituições conservativas; (.)substituições semi-conservativas.

1) Alpha galactosidase

```
MELO3C010698.2      ------MKNVCFSLFFSFGLLF------NWVSSQTGPERAAL------------------
MELO3C011771.2      MALSPSSPSVMVLLYFVLSWTFLLGGNGNGVAVAGASRSTALRFAAEFDSVSSRRVLLNN
                            .* . *:* :.  *      * *:   ..  :**
MELO3C010698.2      -----PPRGWNSYDSFSWIISEEEFLNNVE-IVANKLKSKGYEYVIVDYLWYRKKVPGAY
MELO3C011771.2      GLALTPPMGWNSWNHFQCNLNENLIKETADAMVSSGLAALGYEYINLDDCW-------AE
                         ** ****:: *.  :.*: : :..: :*:. * : ****: :*  *       *

MELO3C010698.2      TDSLGFDVIDEWGRMAPDPVRWPSSQGGKGFSEVAKKVHAMGLKFGIHVMRGISTQAVNA
MELO3C011771.2      LDR------DSKGNLVAKASTFPS-----GIKALADYVHRKGLKLGIY------------
                     *       *..*.:....  :**     *:. :*. **  ***:**:

MELO3C010698.2      NTPILDISKGDAYVESGKKWLASDIGIKSRACGWMHNGFMSVNVKSGAGKAFLRSLYQQY
MELO3C011771.2      ---------SDAGIQTCSKKMPGSLGNEEQDA-------------------------KTF
                             .** ::: .*.:...:* :.. .                         : :

MELO3C010698.2      ADWGVDFVKHD-CVFGDDLDLDEITFVSDVLKQLNSTIVYSLSPGTSATPAMAKAVSGLA
MELO3C011771.2      ASWGIDYLKYDNCENTGTSPKERYPKMTKALQQSGRPILFSLCEWGQEDPATWAVNVG--
                    *.**:*::*:* *   .    :  . ::..*:* . .*::**.   .  **   .  *

MELO3C010698.2      NMYRITGDDWDTWNDIVSHFDVTRDFATANMIGTAGLLGKSWPDLDMLPLGWLTDPGSNN
MELO3C011771.2      NSWRTTSDIQDNWISMTTIADQNDKWASYAKPG-------GWNDPDMLEVG--------N
                    * :* *.* .*.* .:.:  * . .:*:    *       .* * *** :*        *

MELO3C010698.2      GPHRTTNLNIDEQRTQMTLWSISKSPIMFGGDLRNIDNTTFSIITNPTLLEINSFSSNNM
MELO3C011771.2      G-----GMTTAEYRSHFSIWALAKAPLLIGCDIRSMDNITMKLLSNKEVIAVN-------
                    *     .:.  * *:::::*:::*:*:::* *:*.:** *:.:::*  :: :*

MELO3C010698.2      EFLKIASTNFRKRIVKWHSRGLEASASPILGLTKCAYSDTTGWITKSVDQGLEKICWKAN
MELO3C011771.2      --------------------------QDKLGV----------------------------
                                              .  **:

MELO3C010698.2      PEHEYQTPFCLYKRGSRVAIDKEAATHRDQVELLSFSTSSVEVCLDATPKRKHSSEAIMR
MELO3C011771.2      -------------QGKKV------------------------------------------
                                 .*..*

MELO3C010698.2      GSFFPCKRHENQKWDLYANGTLANHNSGHCAIVKYKQAKAIPTGVRSWVATGRGGEVYVA
MELO3C011771.2      ----------------YKYGDLE-----------------------VWAGPLSGKRVAVV
                                    *  * *                         *...  *  * *.

MELO3C010698.2      FFNLNNVKTVISAKISDLAQALPGKKLGPNSCKYREEWSGKDFGLVSDLIAAPVESHGSA
MELO3C011771.2      LWNRGLWRANITASWSDIGLC------SSTTVTARDLWQHSS-QVVQHHLTAQVDSHDCK
                    ::* .  .: *:*. **:. .      ...: . *: *. ..  :*.  ::* *:**..

MELO3C010698.2      LFIINCS
MELO3C011771.2      MFVLTPH
                    :*::.
```

Similarity results (http://imed.med.ucm.es/Tools/sias.html software)

```
MELO3C010698.2  100%
MELO3C011771.2  29.11%          100%
                MELO3C010698.2  MELO3C011771.2
```

2) Auxin response factor

```
MELO3C022932.2      ----------------------------MSFSAASNLPSGGPHSGPSSDALYRELWHACA
MELO3C035167.2      MEIDLNQTASEVGKNAYCYGNCEEGLCNCCLSSSTSSCSSNSSSTPVSSSTYLELWHACA
                                                 .:*:::.  *... * * *.: * *******

MELO3C022932.2      GPLVTLPRQDERVYYFPQGHMEQLEASMHQGLEQQMPSFNLPSKILCKVVNVVLRAESDT
MELO3C035167.2      GPLTSLPKKGNVVVYFPQGHLEQISSASPFS-PMEMRTFDLQPQILCRVINVHLLANKEN
                    ***.:**.:.: * ******:**:.::   .   :* :*:* .:***.*:** * *:.:.

MELO3C022932.2      DEVYAQITLLPESNQNEVTSPDPPLPE--------------PTRCNVHSFCKTLTASDTS
MELO3C035167.2      DEVYTQLTLRPLPELLGTGVAGKELEELALNGADGDGSGGSPTRSTPHMFCKTLTASDTS
                    ****:*:** * .:   .  ..  * *              ***.. * ***********

MELO3C022932.2      THGGFSVLRRHADDCLPPLDMSQQPPWQELVATDLHGSQWHFRHIFRGQPRRHLLTTGWS
```

```
MELO3C035167.2      THGGFSVPRRAAEDCFPPLDYTQLRPSQELIAKDLHGVEWRFKHIYRGQPRRHLLTTGWS
                    ******* ** *:**:**** :*  * ***:*.**** :*.*.**:**************

MELO3C022932.2      VFVSSKKLVAGDAFIFLRGENGELRVGVRRLMRQLNNMPSSVISSHSMHLGVLATASHAI
MELO3C035167.2      IFVSQKNLISGDAVLFLRGENGELRLGIRRAVRPRNGLPDSIVGNQNSCADDLARVVKAI
                    :***.*:*::***.:**********:*:** :*  *.:*.*::..:.   . ** . :**

MELO3C022932.2      STGTLFSVFYKPRTSRSTFLVSLNKYLEAQNHKLSVGMRFKMRFEGEEVPERSFSGTIVG
MELO3C035167.2      STKSTFDVFYNPRAYHAQFIISCQKYVKSINNPVSVGTRFKMRFEMDDSPERRFNGVVVG
                    ** : *.***:**: .: *::* :**::: *: :*** ******* :: *** *.*.:**

MELO3C022932.2      LGDNASPGWANSEWRSLKVQWDEPSSILRPDKVSAWELEPLVASNPLSSQPTQRNKRPRP
MELO3C035167.2      ISDMDPFRWPNSKWRCLTVRWDKDSD--HQERVSPWEIDPSVSLPPLSVQSSPRLKKLR-
                    :.*  .  *.**:**.*.*.**: *.  . :.**.**::* *:  *** *.: * *. *

MELO3C022932.2      TVLPSSSPDATV------------------------------------------------
MELO3C035167.2      TSLQAAPPNNSFTGRGGFMDFEDSVRSSKVLQGQENVGIVSPFYGCDTVKRSLEFDVRSS
                    * * ::.*: :.

MELO3C022932.2      --------------LGGWKPTVESSAFS-YTEPQR------ARDLYSSPKFSTAASNSLG
MELO3C035167.2      AQQNQVSGGVEKLNIGDYVKVHSNSSFTGFMESDRFLKVLQGQEICSLRPPTSKPEYSLG
                                  :*.:  . ..*:*: : *.:*      ..:: *    :: .. ***

MELO3C022932.2      FNANGAVSSNTYWCNTNRVENIMDTSSHGANREPVEKKQNSRNGCRLFGIQLLGNSNVDE
MELO3C035167.2      VWGKFNLSDNSFNTFQSPNSNFYHMASNSAQKMYFPRSEMHSTGQA--AVMLSNDSNFPR
                    ...:  :*.*::.   .  .*:   :*:.*:.  . ..:   .*    .: * .:**.

MELO3C022932.2      ASPVSTPKMVGE-------DRLVSPVDSE-FEQHSEP----SNIHRSDIPSISCDADKSC
MELO3C035167.2      ESALFNPSAVGANVTRTRMERTNRSLDRESLHLVSAPPTLGSNMRNSKGENVNDNATGCK
                     *.: .*. **        :*   .:* * :   * *    **:..*.  .:. :*  .

MELO3C022932.2      LI-------SPLESQSRQIRSCTKVHMQGIAVGRAVDLTRFNQYDDLLRKLEEMFDIEGE
MELO3C035167.2      LFGFSLTTETATNVESSGKRSCTKVHKQGSLVGRAIDLSRLNGYTDLLSELERLFSMEGL
                    *:       :. : :*   ******* **  ****:**:*:* * *** :** :*.:**

MELO3C022932.2      LCGSLKKWQVVYTDDEDDMMMVGDDPWNEFCSMVRKIFIYTTEEVKRLSPKI--------
MELO3C035167.2      LKDPDKGWRVLYTDNENDVMVVGDYPWHDFCDAVSKIHIYTQEEVEKMTNGVVSDDTQSC
                    * .. * *.*:***:*:*:*:*** **::**. * **.*** ***:.::  :

MELO3C022932.2      --KLPLGGEA----KLSKPDSDMIANHTEDQSSIVGSDC
MELO3C035167.2      LDQAPLCMEASKSSSVGQPDSPPTALKVGSFSAMNGVM-
                      : **  **    .:.:***   * :. . *:: *
```

Similarity results (http://imed.med.ucm.es/Tools/sias.html software)

```
MELO3C022932.2  100%
MELO3C035167.2  50.07%          100%
                MELO3C022932.2  MELO3C035167.2
```

3) Beta glucosidase

```
MELO3C015214.2      ----------------MDPTIMSHIKRSNFPKNF----------------------VVGC
MELO3C034277.2      HSNHFKLNKYSFGNWSPKSTTWPSSKPFNFSSSFSFPLKVLPKTKKSKDQIFQTISSLEL
                                     ..*  .  *  **...*                       :

MELO3C015214.2      ASSAYQYEGAAFKYGRGPSIWDTYTHQHPERIDDGSNAVVAVDQYHRYREDVAIMKGIGF
MELO3C034277.2      PHPPTRLKGGFVEDGRGKSNWDVFSHI-PGKIKDNDTGDVADDHYHRFTEDIELMSSMGM
                    . .. . :*. .: *** * **.::*  * .*.*.... ** *:***: **: :*..:*:

MELO3C015214.2      DAYRFSISWPRVLPTGKLSGGVNQEGIDYYNRLIDELISKGIKPYVTLFHWDVPQALEYT
MELO3C034277.2      NAYRFSISWTRILPKGRF-GEVNRRGIIFYNKIIDNLLLKGIEPFVTIHHHDLPDELDKR
                    :********.*:**.*.: * **. ** :**.:**:*: ***:*:**:.*.*:*: *:

MELO3C015214.2      YQGFLGQQIIDDFRDFAELCFKEFGDRVKHWITFNEQYIFAAYGYATGLFAPGRGSSQQA
MELO3C034277.2      YGSWMSSQMQEDFVYFARICFEEFGDRVKHWITINEPNLVTLMAYIRGVYPPAHCSPPFG
                    * .::..*: :**  ** :**:***********:**  :.:  .*  *::.*.. *.  .

MELO3C015214.2      DCVDGDTGTKKPNHIGLLPRRGCFLKSLRYLGGDPGSEPYIVGHHQILAHAKTVKLYKSK
MELO3C034277.2      NCSAGNSDI----------------------------EPLIVMHNMLLAHAKAVFLYRTH
                    :*  *::.                             ** ** *: :*****:* **.::

MELO3C015214.2      Y-KHQNGQIGVTLNTDWYVPYSNSEEDRKATSRALDFSLGWFLHPLVYGDYPASMRDLVK
MELO3C034277.2      FQKKQGGSIGITAVGHMYEPLTNHEFDIEAVDRALIFNFAWVYDPIVYGDYPKEMREVFG
```

```
                    : *:*.*.**:*     * * :* * * :*..*** *.:.*.  *:****** .**::.

MELO3C015214.2      KRLPKFKDDEIVLVKGSYDFLGINYYTANYAKNNPNVD-PNKPSQVTDAHADVSTDRDGV
MELO3C034277.2      SQLPSFSKTEKSIIKGSLDYICVNHYTTLYTKDCLHSPCSNGGDRPIKGFLDTTGYRDSV
                    ..**.*.. *  ::*** *:: :*:**: *:*:  :   .*  ..  ... *.:  **.*

MELO3C015214.2      SIGPKVRKDSWLAVYPQGLKDLMIHIKKYYRDPPIYITENGY--LDYDCPNVEKLIKDEG
MELO3C034277.2      SIGDPTGVDRFFVV-PRGLEKIINYIRQRYPNNPIVVTENGYSMPPSDGKTLEDIINDTK
                    ***  .  * ::.* *.**:.:: :*.: * : ** :*****     *  .:*.:*:*

MELO3C015214.2      RVKYHQQHLIKLHESIKAEVNIKGYFAWTLLDDFEWSRGYTMRFGITYVDFKHKTLERIP
MELO3C034277.2      RVNFHKNYLASLVRAMRNGADVRGYFVWSLMDNFEWIDGFTTRFGLWYVDF--QTLERRP
                    **::*:::* .*  ::.  .::.***.*:*:*:***  *:* ***: ****  :**** *

MELO3C015214.2      KHSARWFNHFLHT------------------------
MELO3C034277.2      KLSAHWFASFLGGNLHQLTKSSSIIDKKNTFRSLTYD
                    * **.**  **
```

Similarity results (http://imed.med.ucm.es/Tools/sias.html software)

```
MELO3C015214.2  100%
MELO3C034277.2  49.31%          100%
                MELO3C015214.2  MELO3C034277.2
```

4) DNAJ

```
MELO3C010495.2      -MVDLPQALGISFR----NFCKSYKSFFKKWHSKVKNSKKDTDNVVDTKHDDATSDAIHN
MELO3C012052.2      MGVDYYNVLKVNRNANEDDLKRSYKRLAMKWHPD--------KNPCNKKEAEA-------
                      **  :.* :. .    :: .*** :  ***..        .*  :.*  :*

MELO3C010495.2      HSPSKDHEEIKKKEGPRPTTTTRGVHSFRYGGRSLRENDTTSFRPRSYDSGYSTLSRNAS
MELO3C012052.2      -----KFKQISEAYDVLSDAKKRQIYDL-YGEEALKSADFVP--PPNSNPSFSYVPRDAD
                         ..::*.:  .  . :..* ::.: **  :*.. * ..  * . :..:* :.*:*.

MELO3C010495.2      RRGQNPASTSSSLFRSVSRRSNESMPSRVSSGRRSIDSISSSPVLSKSGSRRSTTPIMFS
MELO3C012052.2      -------DIFAEFFGGAG--------SGKSRGFRGEGLFKNGKAEAVKQTNR--------
                           .  :.:* ...        *  * * *. . :... . : . :.*

MELO3C010495.2      NSSGVLKAAPIEKQLECTLEELCFGCMKKIKIMDIIMDR--QAMEEEETLTIKVKPGWRK
MELO3C012052.2      ------KAPPIESKLLCSLEELYKGSRRKMRISRTVADEFGKPKTVDEVLKIDIKPGWKK
                          **.***.:* *:****  *. .*:.*   : *   :.   :*.*.*.:****.*

MELO3C010495.2      GTKITFEGGTGNERAGSYPADTSFVIAEKRHSYFKREGDDLELMVEIPLLKALTGCTIPV
MELO3C012052.2      GTKITFP-EKGNQEPGVAPADLIFIIDEKPHPVFERDGNDLVVNQKMSLLEALTGKTLNI
                    ******   .**: .*  ***  *:* ** *. *:*:*:** :  ::.**:**** *: :

MELO3C010495.2      PLLGGETMSLEIHEVVSPGYEKLIQGQGMPKLKDPNNRGNLIVKFFVHFPTQLTPQQRSD
MELO3C012052.2      TTLDGRDLT-TVTDIVKPGYEVVFQNEGMPISKEPNKKGNLRIKFDIVFPSKLTFEQKSD
                    . *.*  ::  : ::*.**** ::*.:***  *:**:.*** :** : **::** :*.**

MELO3C010495.2      VCRILEASHYPS
MELO3C012052.2      LRRALGGSD---
                    : * * .*
```

Similarity results (http://imed.med.ucm.es/Tools/sias.html software)

```
MELO3C010495.2  100%
MELO3C012052.2  45.39%          100%
                MELO3C010495.2  MELO3C012052.2
```

5) Galactinol-sucrose galactosyltransferase

```
MELO3C010314.2      MALNRWLFNSPVLTINTSRFFLSSSFPWSIVSDFNLVTTKKEHSVSDLRICTFTRSRHSS
MELO3C023110.2      ------------------------------------------------------------
MELO3C009979.2      ------------------------------------------------------------


MELO3C010314.2      VKAFKVNNLKGRELESELKAKEMTIKPAVRISDGKLIVKDRTILTGVPDNVIATSGSSSG
MELO3C023110.2      ----------------------MTVTPKISVNDGNLVVHGKTILTGVPDNIVLTPGSGLG
MELO3C009979.2      ----MAPSLKNGGSNVVSFDGLNDMSSPFAIDGSDFTVDGHLFLSDVPENIVASPSPYTS
                                            :.. . :....: *..: :*:.**:*:: :...  .
```

```
MELO3C010314.2      P-----VEGVFLGAVFEEEQSRQVVSLGTLRDVRFMACFRFKLWWMAQKMGDKGKEIPLE
MELO3C023110.2      L-----VAGAFIGATASNSKSLHVFPVGVLEGTRFLCCFRFKLWWMTQRMGTSGRDIPFE
MELO3C009979.2      IDKSPVSVGCFVGFDASEPDSRHVVSIGKLKDIRFMSIFRFKVWWTTHWVGRNGGDLESE
                            * *:*   .: .* :*..:* *.. **:. ****:** :: :* .* ::  *

MELO3C010314.2      TQFLLLETKDGSHLESDDGNEENQIIYTVFLPLIEGSFRACIQGNGQDELELCLESGDVD
MELO3C023110.2      TQFLLMESKGNDGEDPDNSS----TIYTVFLPLLEGQFRAALQGNEKNEMEICLESGDNT
MELO3C009979.2      TQIVILEKSDSGRP------------YVLLLPIVEGPFRTSIQPGDDDFVDVCVESGSSK
                    **::::*.....              *.::**::** **:.:* . .: :::*:***.

MELO3C010314.2      TKASSFTHSLFIHAGTDPFDAISDAMKAVKLHLNTFRLRHEKKLPAIVDYFGWCTWDAFY
MELO3C023110.2      VETNQGLSLVYMHAGTNPFEVITQAVKAVEKHTQTFLHREKKKLPSFLDWFGWCTWDAFY
MELO3C009979.2      VVDASFRSVLYLHAGDDPFALVKEAMKIVRTHLGTFRLLEEKTPPGIVDKFGWCTWDAFY
                    .   .    :::*** :**  :.:*:* *. *  **   .:*. *.::* **********

MELO3C010314.2      HEVTQDGVEAGLESLTAGGVPPKFVIIDDGWQSVGGDPQEEKEEGD----EKQPKQAPLL
MELO3C023110.2      TDVTAEGVVEGLKSLSEGGAPPKFLIIDDGWQQIEAKPKDADCVVQ----EGAQFASRLS
MELO3C009979.2      LTVHPQGVIEGVKHLVDGGCPPGLVLIDDGWQSIGHDSDPITKEGMNQTVAGEQMPCRLL
                      *  :**  *:: *  ** ** :::******.:  ...                 . *

MELO3C010314.2      RLTAIRENSKFQK-----EEDPTEGIKNIVNIAKNKYG-LKYVYVWHAITGYWGGVRTGV
MELO3C023110.2      GIKENHKFQKNGN-----NYDQVPGLKVVVDDAKKQHK-VKFVYAWHALAGYWGGVKPAS
MELO3C009979.2      KFQENYKFRDYVNPKATGSGDGQKGMKAFIDELKGEFKTVEYVYVWHALCGYWGGLRPQV
                     :    :  .  :     . *   *:* .::  * :.  :::**.***: *****::.

MELO3C010314.2      KDMEEYGSSMQYPKVSKGVFENEPIWKNDALALQGLGLMNPKNVYKFYNELHSYLASAGI
MELO3C023110.2      PGMEHYDSALAYPVQSPGMLGNQPDIVVDSLAVHGIGLVHPKKVFNFYNELHSYLASCGI
MELO3C009979.2      PGLPEAR--VIQPVLSPGLQMTMEDLAVDKIVLHKVGLVPPEKAEEMYEGLHSHLEKVGI
                     .: .    :  *  *:  .      * :.:: :**: *::. ::*: ***:* . **

MELO3C010314.2      DGVKVDAQSILETLGAGLGGRVELTRQYHQALDASVARNFPDNGIIACMSHHTDAVYC-A
MELO3C023110.2      DGVKVDVQNIIETLGAGHGGRVTLTRSYHQALEASIARNFSDNGCIACMCHNTDSLYS-A
MELO3C009979.2      DGVKIDVIHLLEMLCEDYGGRVDLAKAYYKAMTKSINKHFKGNGVIASMEHCNDFMFLGT
                    ****:*.  ::* *  . **** *:: *::*:  *: ::* .** **.* * .* ::  :

MELO3C010314.2      KQTAVVRASDDFYPRDPVS--------HTIHIAAVAYNTVFLGEIMLPDWDMFHSLHSAA
MELO3C023110.2      KQTAVVRASDDYYPRDPAS--------HTIHISSVAYNSLFLGEFMQPDWDMFHSLHPTA
MELO3C009979.2      EAISLGRVGDDFWCTDPSGDPNGTFWLQGCHMVHCAYNSLWMGNFIHPDWDMFQSTHPCA
                    :  :: *..**::  ** .        :  *:   ***:::*:::  ******:* *. *

MELO3C010314.2      EYHASARAISGGPVYVSDAPGKHNFELLRKLVLPDGSVLRATLPGRPTRDCLFSDPARDG
MELO3C023110.2      EYHGAARAIGGCAIYVSDKPGNHNFDLLKKLVLPDGSVLRAQLPGRPTRDSLFNDPARDG
MELO3C009979.2      AFHAASRAISGGPIYVSDSVGKHNFDLLKKLVLPDGSILRSEYYALPTRDCLFADPLHNG
                     :*.::***.* .:****  *:***:**:********:**:   . ****.** ** ::*

MELO3C010314.2      VSLLKIWNLNKFTGVVGIYNCQGAAWNSQERKNTFHDTNSDAITGYVKGRDVHAIS-EVA
MELO3C023110.2      TSLLKIWNMNKCSGVVGVFNCQGAGWCRITKKTRIHDESPGTLTTSVRAGDVDAIS-QVA
MELO3C009979.2      ETMLKIWNLNKFTGVIGAFNCQGGGWCRETRRNQCFSQYSKRVTSKTNPKDIEWHSGENP
                     ::*****:** :**:* :****..*    ::.  ..  .  :*  ..  *:.  * : .

MELO3C010314.2      ADPNWNGDCAFYRHRSGDLITLPYNSALPVSLKVLEFDIFTITPIKVLAPG-FSFAPLGL
MELO3C023110.2      G-ADWKGDTIVYAYRSGDLIRLPKGASVPVTLKVLEYDLFHISPLKDIASN-ISFAPIGL
MELO3C009979.2      ISIEGVKTFVLYLYQAKKLIISKPSQDLDIALDPFEFELITVSPVTTLTQTSLHFAPIGL
                       :      .* ::: .**    .  : ::*. :*:::: ::*:. ::   : ***:**

MELO3C010314.2      IDMYNSGGAIEGLKYEVKGGAELVEVDGTSEGTEAAGERAENRSSELVGIVHLEVKGCGK
MELO3C023110.2      LDMFNTGGAVEQVNVQVVEPIPEFDGEVASELTCSLPD-----DRPPTATITMKARGCGR
MELO3C009979.2      VNMLNTGGAIQSVDYDDD----------------------------LSSVEIGVKGCGE
                    ::* *:***:: :. :                                . : : .:***.

MELO3C010314.2      FGAYSSAKPRRCIVDSSVVEFGYDSESGLLTLGIDKLPEGDLKYHDIKIEL-
MELO3C023110.2      FGLYSSQRPLKCSVDKVDVDFVYDEVTGLVTFEIPIPTEEMYRWNIEIQV--
MELO3C009979.2      MRVFASKKPRACRIDGEDVGFKYDQDQ-MVVVQVPWPVDSSSGGISVIEYLF
                    :  ::* :*  * :*   * * **.   ::.. :    :
```

Similarity results (http://imed.med.ucm.es/Tools/sias.html software)

```
MELO3C010314.2  100%
MELO3C023110.2  53.23%  100%
MELO3C009979.2  37.24%  37.69%  100%
```

6) Hexosyltransferase

```
MELO3C021249.2      MAFFTRKQPLLNSLPQSHSPSSSSSSSSSSSSSSSSQNLFKFINKLRRSRSSSLILFFIF
MELO3C015949.2      ----MRRRPPDFRRPARRRLSNA--------------------------------FWWT
MELO3C019691.2      --MASKRGLLSATAAHRARAGGGSRIP---------------------------LLLVIF
                         ..       .     ...                                 :

MELO3C021249.2      LFFILLLCFLFLISFT-----LLSSNSNSNSTTITNS--LREIPTVIDSEFQETI-----
MELO3C015949.2      LCFVAVLLFVF----------ILTKGPQ-----------IESTRPVLARR----------
MELO3C019691.2      FFFLSPVLFFFGRGFRAADLEIISSGSGQQDVGWRERIALHQFKSLFSKEVIDVIAASTN
                    : *:  : *.*          :::...            : .  .::

MELO3C021249.2      KIGLINISTDDKDEIHDTLHLLGLIENRRSR-----------------------------
MELO3C015949.2      --------SYRHEGIMEGLNITEEMLNPSSI-----------------------------
MELO3C019691.2      DMGPYSLDHFRKNNFSASWKINGQEVTPDGIPERNRMVVDLGKEKSDSEEVKLMDDSSQS
                               :: :    ::     .  .

MELO3C021249.2      --------------------------------------------IETVTVDFDRIEKEFE
MELO3C015949.2      ------------------------------------------------------------
MELO3C019691.2      TDSPTKQARRLHVQQLREKXREKRAAQLLQQDDDILIKLENAAIERSKSVDTSVLGKYSI


MELO3C021249.2      W------------ERLFPEWIDEDEKYKSSPKCPNIPMPKTEDYSDLNIVVLRIPCKRGI
MELO3C015949.2      -------------ARQLSDQISLAKAFVVIAKESS----NLQFAWELSA---QIRNSQIL
MELO3C019691.2      WRKENENENTDATVRLMRDQMIMARAYLGIAKMKN----KLDLYHELQT---RLKESQRA
                                  * : :.:     :   .*  .    : :   :*.    .:  ..

MELO3C021249.2      LTSRDVFRLQANLAAARVAVASGWVEEEIYRTVYVVFLGECGAMREIFRCDDLVIKDDRT
MELO3C015949.2      LSSAATRRAPLQITEAETAIRD--MALLLYQAQQLHY--DSATM--IMRLKAKIQTLDEQ
MELO3C019691.2      LGEAST-DADLNRSAPDK-IKS--MGQILSKAKEQLY--DCKLV--TGKLRAMLQSADEE
                    * .  .     : : .   : .  :   : .:    :  :.  :    .    : . *

MELO3C021249.2      VENVWVYKPEIKRLKQKILLPFGSCQLAPVYARTGREVWRHFMLQKPPKTNSTTTHYHKP
MELO3C015949.2      MSSVSDKSSKYGQIAAEEVPKSLYC----LGVRLTTEWFKNLNLQKKLSEEKQIDMKLKD
MELO3C019691.2      VRGLKKQSTFLSQLAAKTIPNGIHC----LSLRLTID-YHLLPLEKRKFPRSE---NLEN
                    : .:   ..   .:  : :     *    :  *   : :. : *:*     .      :

MELO3C021249.2      KQAYVTILHSSEAYVCGAIALAQSLLQTNTSKDLILLADNSISPNSIQALKDAGWYVMRI
MELO3C015949.2      NDLYHFCVFSDNILATSVVVNSTALNSKNPDRIVFHLVTDEVNYAAMKA-----WFSM--
MELO3C019691.2      PNLYHYALFSDNVLAASVVVNSTIMNAEDPSKHVFHLVTDKLNFGAMNM-----WFLS--
                     : *   :.*.:  . ..:. :  :   :... :: *. :.:.  :::      *:

MELO3C021249.2      DRIRSPFSEKGSYNEWNY---------SKLRIWQLTMY--------------DKIVFIDA
MELO3C015949.2      NNFRRVTVDVQMFEDFSWLNASYVPVLKQLQDSDTQNYYFSGNGGDSR----TPIKFRNP
MELO3C019691.2      NPPGKATIHVENVDEFKWLNSSYCPVLRQLESAAMKEYYFKAGHPTTLSSGASNLKYRNP
                    :            :::.:          :*       *                : : :.

MELO3C021249.2      DLLVLKNIDQFFALPQLSAAANNKMRFNSGVMIVEPSACLFEELMEKSFELKSYNGGDQG
MELO3C015949.2      KYLSMLNHLRFY-IPEVFPALKKVVFLDDDVVVQRDVSGLFS---------IDLNGNVNG
MELO3C019691.2      KYLSMLNHLRFY-LPQVYPKLEKILFLDDDIVVQKDLTGLWD---------VDLHGKVNG
                    . * : *  .*: :*:: .  :: : ::..:::    : *:.          . :*  :*

MELO3C021249.2      FLNEVFTWWHRLPSRVNYLKIFLKENSENDSGTDPYAIHYLGLKPWMCYKDYDCNWDMED
MELO3C015949.2      AVETCMETFHRYHKYLNYSHPLIREHFDPDACGWAFGMNVFDLVEW---RKRNVT---GI
MELO3C019691.2      AVETCGESFHRFDKYLNFSNPHIARKFDPNACGWAYGMNMFDLKEW---KKRDIT---GI
                     ::     :**  . :*: :  :  : : ::   .:.:: :.*  *   .. : .

MELO3C021249.2      HQIFASDSAHAKWWQIYESMPTELQHFCGLTKKMDSRIRKWRRIARNNSTFADAH-----
MELO3C015949.2      YHYWQEKNVDRTLWKL-GTLPPGLLTFYGLTEPLD---PSWHVLGLGY-TNVDSQLIEKG
MELO3C019691.2      YHKWQNLNEERLLWKL-GTLPPGLITFYGLTHPLD---KSWHVLGLGYNPSIDKSEIENA
                    :: : . .     *::  ::*. *  * ***  :*    .*. :. .  .  *

MELO3C021249.2      -----------W-KIKITD--PRRHRLMDDHAP---------
MELO3C015949.2      AVLHFNGNSKPWLKIGIEKYKPLWEKYVDYTHPLLQSCNFH-
MELO3C019691.2      AVIHYNGNMKPWLELAMTKYRGYWTKYIKYNHPYLRQCKLNE
                               * :: : .    . . :.   *
```

Similarity results (http://imed.med.ucm.es/Tools/sias.html software)

```
MELO3C015949.2  100%
MELO3C019691.2  56.17%          100%
MELO3C021249.2  18.72%          18.72%          100%
                MELO3C015949.2  MELO3C019691.2  MELO3C021249.2
```

7) Invertase inhibitor

```
MELO3C008049.2      --MGMKNFSISLIFFAIPLIFFHKNGVSLASADQTLIQKTCTNTLYYKLCMSSLKSDPSS
MELO3C006266.2      MSFTYTHRYIALAFSFVVLLNFEPISANDIVSRTCETSAARDPNVRLDFCLRSLAAAPGS
                      :  .:  *:* *  : *: *.  ...   :     . :   .:  .:*: ** : *.*

MELO3C008049.2      LTADTKGLAIIMASIGAANATATSTYLSSQLPTSSSAATNANNNKTKLLRQCSEKYAFAA
MELO3C006266.2      DTADLYELGAISIRLIGRNATSTQRYIERLLKNEKKKSSSDSYIRPRLS-DCEELYSDAV
                     ***   *. *   : . ***:*. *:.  * .... ::. .  :.:*  :*.* *: *.

MELO3C008049.2      EALRESLKDLADETYDYAYMHVSAAADYANVCRDAFKGFPAVSYPAKLGRREEGLKRICR
MELO3C006266.2      ETVGEAAAEYGRKRYDEVNVKLSSVMDAVTTCEDGFKEMESRVSP--LTKRNGDVFELAA
                    *:: *:  : . : ** . :::*:. * ...*.*.** : :   *  * :*: .: .:.

MELO3C008049.2      VVLGILDLLGW
MELO3C006266.2      IALCILDLRP-
                    :.* ****
```

Similarity results (http://imed.med.ucm.es/Tools/sias.html software)

```
MELO3C008049.2  100%
MELO3C006266.2  32.08%          100%
                MELO3C008049.2  MELO3C006266.2
```

8) Polygalacturonase

```
MELO3C011986.2      MPTKMASLFSFINSNLFLSIIALIQITLVISSNNSTEMDKLFLLSLLGLLIIAYGVSGTF
MELO3C023627.2      MP--MEKLY---KNPIKAQVMKMVILGVIMAAALMDEVE---------------GRRGKV
                    **  * .*:   :. :  .:: :: : :::::    *::               *  *..

MELO3C011986.2      VSDSDIDMIEELESFDIEEEDDDVVYSYTVPAWRSERGSKILMNVDSFDAVGDGVTDDTM
MELO3C023627.2      KKGKKAKI---LEGNGIE---------YGAINCRAHTAS-----ITEFGGVGDGVTSNTK
                     .... .:   **. .**         * .   *:  .*     : .*..******.:*

MELO3C011986.2      AFRKAWETA---CSTSKSVLLVPKEKRYLVNATTFKGPCKDGMVIQIEGTIVAPDEPELW
MELO3C023627.2      AFQAAVERLGQYANDGGAQLYVPAG-RWLTG--SFNLTSHFTLFLHQDAVLLASQDPNEW
                    **. * *     .. . : * **   *:*..  :*: ..:  :.:: :..::*.::*: *

MELO3C011986.2      D-----PKFPR-------QWLEF---TKLNAATFQG-DGVIDGSGEKWWAESCKKNKSRP
MELO3C023627.2      PVVEPLPSYGHGRDTSGGRYISLIFGTNLTDVVITGNNGTIDGQGDRWW-QLFHQGKLKY
                          *.: .       .::.:   *:*. ..: * :*.***.*:.** :  ::.* .

MELO3C011986.2      CKGAPTAFTIDSSSNIRVKGLTIQNSQQMHFTIARSETVRITEVRVSAPGDSPNTDGIHI
MELO3C023627.2      TR--PYLIELMYSRDIQISSLTLLNSPAWNVHPVYSSNILIQGITIIAPVRSPNTDGINP
                     .  *  : :  * :*.:..**: **   :.  . *..: *  : : **  *******:

MELO3C011986.2      TQSTNVVVQNSKISTGDDCISIVNA--SSGIK---------MKGISCGPGHGISIGSLGK
MELO3C023627.2      DSCTNVRIEDCYIVSGDDCVAVKSGWDEYGIRFGLPTKQLVVRRLTCISPTSAVI-ALGS
                     ..*** :::. * :****::: ..  . **.         :. ::* .  .  * :**.

MELO3C011986.2      DNSTGIVTKVVLDTAFLRETTNGVRIKTWQGGSGYVRSVRFENVRMEDVENPIIIDQFYC
MELO3C023627.2      EMSGGI-QDVRAEDIVAINSESGVRIKTGIGRGGYVKDIYVRRMTMHTMKWAFWMTGDYG
                    : * **  .*  :  .  :: .******  * .***..: . .: *  ::..: :   *

MELO3C011986.2      DSPTTCETQTSAVKISQIMYRNISGTTTSKNAMKFACSDSVPCSNIILSNVNLEKTDGTV
MELO3C023627.2      SHADKNYDPHALPVIQGINYRDMV-VENATMAARLEGISGDTFTDICISNVTIGLAPKAK
                    . . .     :   *. * **::  . .:. * .:   .. . ::* :***.:  :  :

MELO3C011986.2      ET--YCHAAQGFGYGIVHPSADCLTSSDNNFAALDQ--TQVLELETVQLPTEQHIVHTEL
MELO3C023627.2      KQPWTCTDVEGITSQVTPPPCDLLPDQGEEKITTCKFPEDNLPIDAVQLKKCSYQFSHA-
                    :    *  .:*:   :. *..* *....::  :  :   : * :::*** . .: .
```

Similarity results (http://imed.med.ucm.es/Tools/sias.html software)

```
MELO3C011986.2  100%
MELO3C023627.2  26.97%          100%
                MELO3C011986.2  MELO3C023627.2
```

9) Sucrose synthase

```
MELO3C015552.2      MAERVLNRVH--SLRERLDETLIAQRNEILGLLSKIEAKGKGILQHHELIAEFETIPEE-
MELO3C025101.2      MASLVVNHHNGESIGDGIVEALKQNQNYMKTCFGKFVEKGNRSLKKKELMEEMELVIDDK
```

```
                    **. *:*. :  *: : : *:*  :.* :   :.*:  **:  *:::**: *:* : ::

MELO3C015552.2      -NRKKLADGAFGEVLRSTQESIVLPPWVALAVRPRPGVWEYIKVNVHALVVEELQVSEYL
MELO3C025101.2      IERNRVMEGILGHMLTSTQVAIVIPPYVAFAIRPEPGCWEYVKVSSLDLSLQSLTSTEFL
                     :*:.: :* :* :* *** :**:**:**:*:** ** ***:**.   * ::.*  :*:*

MELO3C015552.2      RFKEELVDGS-SNGNFVLELDFEPFNASFPRPTLSKSIGNGVEFLNRHLSAKLFHGKESM
MELO3C025101.2      KLKEMIYDEEWANDENALEVDFGAIEFTTPHLSLPSSIGDGLSYTTKFLTSKLSGKLENL
                    .:** : * . :*.: .**:** .:: : *. :*..***:*:.: ...*::**    *.:

MELO3C015552.2      QPLLDFLRVHCYKGKTMMLNDRIQTLNAFQHVLRKAEEYLVTLAPDTPYSDFANKFQEIG
MELO3C025101.2      QPLVDYLLSLDYQGEKLMINETLNTPSKLQMALILADIFLSVLPPHTPYDDFHLKLKQWG
                    ***:*:*    *:*:.:*:*: ::* . :* .*  *: :* .*.* ***.**  *::: *

MELO3C015552.2      LERGWGDTAERVLEMIQLLLDLLEAPDPCTFEKFLGRIPMVFNVVILSPHGYFAQDNVLG
MELO3C025101.2      FERGWGDCAGRVKETMRCLSEIFQAYDPIQMEKFFSRLPTTFNVVILSPHGYFGQAGVLG
                    :****** * ** * :. * ::::* **  :***:.*:* .***********.* .***

MELO3C015552.2      YPDTGGQVVYILDQVRALEHEMLQRIKQQGLDITPRILIIITRLLPDAVGTTCTQRLEKVF
MELO3C025101.2      LPDTGGQVVYILDQVKAMEEELLLRIKQQGLNFKPQIIIIITRLIPDAKGTKCNQEIEPVI
                     *************.*:* *:* *******::.*.*:*****:*** **.*.* :* *:

MELO3C015552.2      GTEHSHILRVPFRNEKGIVRKWISRFEVWPYLETYTEDVAQELTKELQGKPDLIIGNYSD
MELO3C025101.2      GTNYSKIVRVPFKTENGILHRWVSRFDIYPYLEKFAQDASDKILELMEAKPDLIIGNYTD
                    **::*:*:****..*:**:..*:***::****.:::*.:::: : ::.********:*

MELO3C015552.2      GNIVASLLAHKLGVTQCTIAHALEKTKYPDSDIYWKRFDEKYHFSSQFTADLIAMNHTDF
MELO3C025101.2      GNLVASLMASRLGVTQGTIAHALEKTKYEDSDLKWKELDSKYHFSCQFTADILAMNATDF
                    **:****:* .***** *********** ***: ** :*.*****.*****::*** ***

MELO3C015552.2      IITSTFQEIAGSKDTVGQYESHTAFTLPGLYRVVHGIDVFDPKFNIVSPGADMSIYFPYT
MELO3C025101.2      VIASTFQEIAGSKEKPGQYESHEAFTLPGLCRVVSGINVFDPKFNIAAPGADQSVYFPYT
                    :*:**********:. ****** ******* *** **:********.:**** *:*****

MELO3C015552.2      ETEKRLTSFHPEIEELLYSEVENEEHLCVLKDRSKPIIFTMARLDRVKNITGLVEWYGKN
MELO3C025101.2      AKELRFASFQPAIEELLFSKVENNEHIGYLADRKKPIIFSMARLDVVKNITGLVEWFGKN
                     .* *::**:* *****:*:***:**:  * **.*****:***** **********:***

MELO3C015552.2      NRLRELVNLVVVAG--DRRKESKDNEEKAEMEKMYNLIKTYNLNGQFRWISAQMNRVRNG
MELO3C025101.2      EKLRNLVNLVVVGGFFDPSK-SKDREEMAEIRKMHELIDKYQLKGQIRWIAAQTDRRRNG
                    :.**:*******.*  *  * ***.** **: **::**..*:*:**:***:** :* ***

MELO3C015552.2      ELYRCIADTKGAFVQPAVYEAFGLTVVEAMTCGLPTFATCNGGPAEIIVDGKSGFHIDPY
MELO3C025101.2      ELYRCIADTKGAFVQPALYEAFGLTVIEAMNCGLPTFATNQGGPAEIIVDGVSGFQIDPN
                    ****************:********:***.******** :********** ***:***

MELO3C015552.2      RGDRAAEILVDFFEKSKEDPTHWDKISQAGLKRIYEKYTWQIYSERLLTLTGVYGFWKHV
MELO3C025101.2      NGNESSQKIANFFEKCKNDPTYWNEISNHGLQRINECYTWKIYAKKVLNMGSTYTFWKQV
                    .*: ::: :.:****.*:***:*::**: **:** * ***:**::.:*.: ..* ***:*

MELO3C015552.2      SNLDRLESRRYLEMFYALKYRKLADSVPPAVDE---------------------------
MELO3C025101.2      NKNQKQAKDRYIQMFYNLLFKNLVKTVPIVVDENPHPGNPQLPQVSKRTQSRIQKFFGAP
                    .: :.  . **::*** * :.:*..:** .***
```

Similarity results (http://imed.med.ucm.es/Tools/sias.html software)

```
MELO3C015552.2  100%
MELO3C025101.2  65.5%           100%
                MELO3C015552.2  MELO3C025101.2
```

10) Trehalose phosphate synthase

```
MELO3C013838.2      MVSRSYSNLLELASGGCSPTFGLGRERKRLPRVATVAGVLSELDDDSCNSTGSDAPSSVA
MELO3C018715.2      MISRSYTNLLDLASG---NFPAMEVKKKRFPRVMTVPGNVAELDDDQAYSVTSENPSSIS
MELO3C012010.2      MASRSTANFLDLASG---DLLDVPRTPKSLPRVMTAPGIISELDDYGSNDGDSES-SSVY
                    * *** :*:*:****       :    * :*** *..* ::****  . .  *:  **:

MELO3C013838.2      QDRMIIVGNQLPIRANRNEN-GDWEFSMDEDSLLLQLKDGLGEDVEVIYIGCLREEVDPR
MELO3C018715.2      SDRMIIVANQLPLRAKRREDDNSWSFSWNEDSLLLQLKDGLPEDMEILYVGSLKVDIQPE
MELO3C012010.2      RERKIIVANMLPLHAKKDGQSGKWCFSLDEDSILLQLKNGFSPEMEVIYIGSLKVEIDPS
                     :* ***.* **::*::  : ..* ** :***:*****:*:  ::*::*:*.*: :::*

MELO3C013838.2      EQDDVAQTLLDRFKCVPTFLPPELFSKFYHGFCKQHLWPLFHYMLPLSPDLGGRFDRSLW
```

```
MELO3C018715.2      EQDEVSQILLERFRCIPAFLPPHILEKFYDGFCKRHLWPLFHYMLPYSADQKGRFDRSMW
MELO3C012010.2      EQEEVAQKLFDNFNCVPTFLPHDLQKNFYIGFCKQQLWPLFHYMLPMCPEHGDRFDRQLW
                    **::*:* *::.*.*:*:*** .: .:** ****::********** ..:  .****.:*

MELO3C013838.2      QAYLSVNKIFADKVMEVISPDDDFVWVHDYHLMVLPTFLRKRFNRVKLGFFLHSPFPSSE
MELO3C018715.2      EAYVSANKLFFQKVVEVINPDDDYIWIHDYHLMVLPTFLRRRFNRVKMGFFLHSPFPSSE
MELO3C012010.2      QAYVSANKLFADKVMEIINPEEDSVWVHDYHLMVLPTFLRKRYNRVKLGFFLHSPFPSSE
                    :**:*.**:* :**:*:*.*::* :*:************:*:****:************

MELO3C013838.2      IYRTLPVRDELLRALLNSDLIGFHTFDYARHFLSCCSRMLGLSYQSKRGYIGLEYYGRTV
MELO3C018715.2      IYRTLPVREEILKALLNSDIIGFHTFDYARHFLSCCSRMLGLEYQSKRGYLGLEYYGRTI
MELO3C012010.2      IYRTLPVRDEILRGLLNCDLLGFHTFDYARHFLSCCSRMLGLDYESKRGHIGLDYYGRTV
                    ********:*:*:.***.*::*********************.*:****::**:*****:

MELO3C013838.2      SIKILPVGIHIGQLQNVLNLPETVSKVAELQDRFKGQTVLLGVDDMDIFKGISLKLLAFE
MELO3C018715.2      GIKIMPVGIHMSRIESVMKLADEDAKTRELTQQFAGKTVLLGVDDLDIFKGINLKLLAME
MELO3C012010.2      YIKILPVGIHMGRLESVMNLPSTFAKVKEIRDQFMGKKLILGIDDMDIFKGISLKLLAVE
                     ***:*****:.:::.*::*..  :*. *: ::* *:.::**:**:******.*****.*

MELO3C013838.2      QLLRQHPERWGKAVLVQIANPARGRGKDVQEVVAETTATVDRINTTFRRPGYEPVVLINT
MELO3C018715.2      QVLKQHPKWIGKAVLIQIVNPARGKGRDLQEIEDEIKESCRRINKEYGHPGYEPIVLLDR
MELO3C012010.2      QLLRQHPALRGKIVLVQIVNPARGSGKDVHEAQKEAYLAAERINEAYGSSNYKPVILIDR
                    *:*:***   ** **:**.***** *:*::*   *   :  ***  :  ..*:*::*::

MELO3C013838.2      PLQFYERIAYYAIAECCLVTAVRDGMNLIPYEYIICRQGNEKLDDVLGLNPSAAKKSMLV
MELO3C018715.2      HVPITERVAYYSIAECVAVTAVRDGMNLTPYEYVVCRXGNIHLPKMFELQR--TKKSMLV
MELO3C012010.2      PVPRFEKTAYYALAECCIVSAVRDGMNLVPYKYIVCRQSTPLMDEALGLQIGSPRTSMLV
                     :   *: ***::***  *:******** **:*::** ..  : . : *:   .:.****

MELO3C013838.2      LSEFIGCSPSLSGAIRVNPWNIEAVTEAMDSALVIPEAEKQLRHEKHYRYVSTHDVAYWA
MELO3C018715.2      ISEFIGCSPSLSGAFRVNPWNVEKTAEALNEAISMADSEKQLRHEKHYRYVATHDVAYWS
MELO3C012010.2      VSEFIGCSPSLSGAIRVNPWDIDAVADALELAITMQESEKQLRHEKHYRYVSSHDIGYWS
                    :*************:*****::: .::*:: *: : ::*************::**:.**:

MELO3C013838.2      RSFLQDLGRACRDHSMRRCWGIGFGLGFRVIALDPDFRKLSVDHIVSVYKRTGYRAILLD
MELO3C018715.2      RSFLQDMERTCSDHFRRRCWGIGFSFGFRVVALDPNFRKLSVDAIVSSYSRAESRAILLD
MELO3C012010.2      RSFMQDLDRACQDHYSKRCWGIGLGLRFRVVSLSPGFRKLTIDHIVSAYKRTHRRAIFLD
                    ***:**: *:* **  :******:.: ***::*.*.****::* *** *.*:  ***:**

MELO3C013838.2      YDGIMTLPGSISMNPTSEALGILNNLCKDPKNVVFLVSGKDRKTLTEWFSPCEKLGLAAE
MELO3C018715.2      YDGTVMPQNSINKSPSAQVISILNTLCADAKNVVFIVSGRGRDSLSKWFCPCKKLGIAAE
MELO3C012010.2      YDGTIISQSSIIKTPSPEVISVLTTLCNDPCNTVFIVSGRGRSSLGQWFVSCEMLGIAAE
                    *** :   .**  .*:.:.:.:*..** *. *.**:***:.*.:* :** .*: **:***

MELO3C013838.2      HGFYLRPNQTADWETCVAVTDFDWKQIAEPVMQLYTETTDGSTIETKESALVWNYLYADP
MELO3C018715.2      HGYFLRQSQNEEWKICGQSSDFGWMHIAEPVMKLYTEATDGSSIERKESALVWQYRDADP
MELO3C012010.2      HGYFIRWGGTSEWETSPLSSDLDWIKIVEPVMRLYTEATDGSCIEQKESALVWHHQDADS
                    **:::* . . :*: .    :*:.* :*.****:****:**** ** *******::  **.

MELO3C013838.2      DFGSCQAKELLDHLESVLANEPVSVKSGQHIVEVKPQGVNKGIVAEYLLQTMKEKGMLPD
MELO3C018715.2      GFGSSQAKEMLDHLESVLANEPVAVKSGQFIVEVKPQGVTKGLVAEKIFSSMAEDGKLAD
MELO3C012010.2      DFGSCQAKELLDHLENVLANEPAVVKRGQHIVEVKPQGVSKGLVAEKVLSRMVDSGRPPD
                    .***.****:*****.******. ** **.*********.**:*** ::. * :.*  .*

MELO3C013838.2      FVLCIGDDRSDEDMFEVIMNAKAS--LSPGAEVFGCTVGQKPSKARYYLEDTHEILRMLQ
MELO3C018715.2      FVLCIGDDRSDEDMFEIIGNALSSNILSPTTSVFACTVGQKPSKAKYYLDDTAEVISMLE
MELO3C012010.2      FVMCIGDDRSDEDMFESILSTVSSPSLPSAPEIFACTVGRKPSKAKFYLDDTSDVVKLLQ
                    **:************* * .: :*  *.. ..:*.****:*****::**:** ::: :*:

MELO3C013838.2      GLTHASEHATRAAPQTTARRVVIAESK
MELO3C018715.2      YLAEASSPLPSSDDEEGENAS------
MELO3C012010.2      CLASSSRPRPRQLPQMRVSFESVF---
                     *: :*   .    :
```

Similarity results (http://imed.med.ucm.es/Tools/sias.html software)

```
MELO3C013838.2  100%
MELO3C018715.2  73.94%          100%
MELO3C012010.2  72.55%          70.67%          100%
                MELO3C013838.2  MELO3C018715.2  MELO3C012010.2
```
